# Theoretical Study of Cu Carbenoids in C–H Activation Reactions: The Interplay between Metal Back-Donation and Electrophilicity of the Carbon

**DOI:** 10.1021/acs.jpca.5c02784

**Published:** 2025-06-10

**Authors:** Sasha Gazzari-Jara, Barbara Herrera

**Affiliations:** QTC, Escuela de Química, Facultad de Química y de Farmacia, 28033 Pontificia Universidad Católica de Chile, Av. Vicuña Mackenna 4860, Macul, Santiago 7820436, Chile

## Abstract

Our study is centered on the reactivity of copper­(I) carbenoids in C–H activation reactions, particularly the role of the carbenoid carbon. The selective activation of inert C–H bonds, leading to the direct transformation of simple hydrocarbons into functionalized molecules, is a key area of organic chemistry research. Our theoretical density functional theory (DFT) study at the M06–2X/cc-PVTZ/LANL2DZ level provides insights into the catalytic C–H activation and alkyl insertion mechanisms using these copper­(I) carbenoids. We analyze the effects of electron-donating (EDG: OH, CH_3_, and NH_2_) and electron-withdrawing groups (EWG: Cl, COOH, CN) on the reactivity, selectivity, and stability of copper carbenoids. Our systematic analysis using conceptual DFT (c-DFT), natural bond orbital (NBO), reaction force (RF), activation strain model (ASM), and energy decomposition analysis (EDA) reveals that the electronic nature of substituents significantly modulates the electrophilicity of the carbenoid carbon, thereby affecting the strength of the Cu–Cα bond, reaction barriers, and the type of mechanism. We identify carbenoids substituted with balanced EDG/EWG pairs as optimal candidates, providing kinetically and thermodynamically favorable pathways for the selective C–H activation via electrophilic substitution involving a σ-complex intermediate.

## Graphical abstract

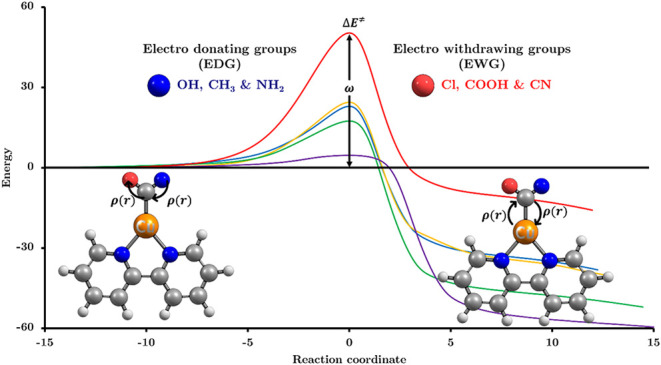

## 1 Introduction

The activation of C–H bonds synthetically is of growing importance because it can be considered a potential site to add functional groups into an organic moiety, building larger and more complex molecules.^1–3^ This process allows for the modification of ubiquitous C–H bonds in organic compounds rather than relying on pre-existing functional groups.^1–3^ The direct conversion of alkanes into functionalized molecules offers the chance to transform inert alkanes from natural sources into versatile building blocks for synthesizing complex molecules.^4^ Furthermore, it adds atom economy by minimizing waste generation and maximizing the utilization of starting materials, which is in alignment with sustainable production and green chemistry principles.^5^

There are various synthetic strategies for activating C–H bonds and their subsequent functionalization. Most of these methodologies involve using metals in catalytic reactions, such as oxidative addition, σ bond metathesis, and electrophilic substitution, among other strategies.^6^ In this context, C–H activation using metal carbenoids has gained significant attention in organic synthesis, offering a more versatile and efficient approach to selective C–H functionalization^7–10^ and unlocking access to a wide range of chemical transformations and expanding the synthetic toolbox available to chemists.^11^

Carbenoids are synthesis intermediaries generated in situ from diazo compounds and a metal complex, either thermally or photolytically,^12,13^ where a metal intermediary is covalently bonded to the electron pair of the C coming from the azide group^14^ (see Scheme 1). Although their nature has been discussed because the original term was coined for *sp* ^3^ carbon atoms,^15^ some research still must be done to address their electronic nature and reactivity. Nevertheless, these systems are highly selective experimentally and react in mild conditions, ensuring high reaction yields in contrast to classic carbenes.^12,13^ The majority of research in C–H activations has been done in rhodium (Rh) and copper (Cu) carbenoids^16–19^ exhibiting the aforementioned reactivity and high turnovers.^20,21^

**Scheme 1:**
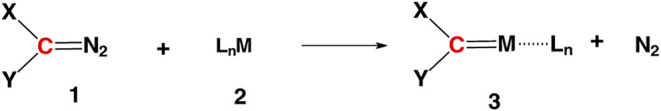
Reaction of a Diazo Compound (**1**) and a Metal Catalyst (**2**) to Form the Carbenoid (**3**)­^a^

a The reacting carbenoid carbon is colored red, while X and Y are its substituents.

Due to this particular carbon–metal bonding, carbenoids present dual reactivities which can be classified based on their reactivity with Fischer carbenoids^22^ (electrophilic) and Schrock carbenoids^23^ (nucleophilic), providing a dual character to their reactivity depending on the combination of the metal and the groups located at the α-position to the carbenoid Cα atom^24^ [see Scheme 2(a)]. These groups can be either electron-withdrawing groups (EWG) or electron-donating groups (EDG), allowing the modulation of the carbenoid properties depending on the target synthesis.^25,26^ If the combination of the EWG and EDG makes the carbenoid too electrophilic, it will exhibit poor regio- and stereocontrol and be susceptible to other competing reaction pathways.^25^ Conversely, a carbenoid with insufficient electrophilicity will lack the reactivity to activate the C–H bond [Scheme 2(b)].

**Scheme 2:**
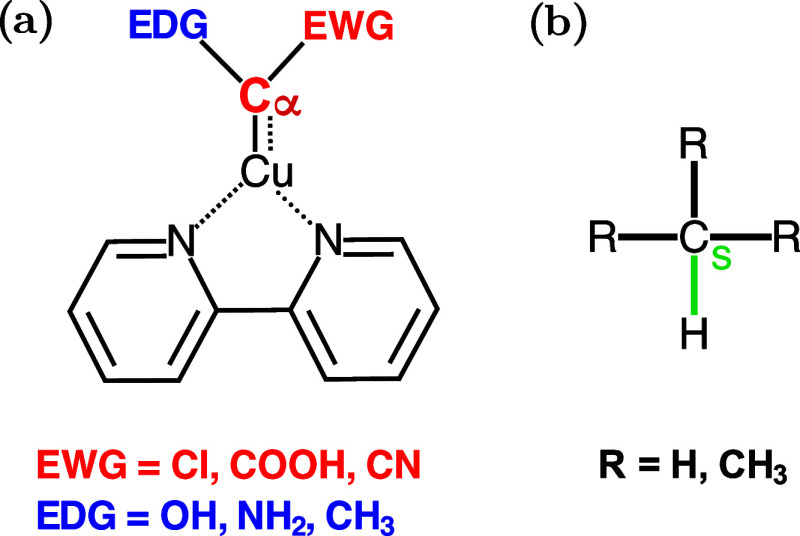
(a) Carbenoids of This Study, EDG (Blue), and EWG (Red), are the Groups Bonded to the Carbenoid Carbon (Cα in Red); (b) Alkyl Substrates and Their Activated Cs–H Bond (Green)

The proposed mechanisms for carbenoid insertion vary, depending on the nature of the carbenoid metal and the substrate. Depending on the charge transfer involved in the transition state, their insertion reactions might be oxidative addition (OA), carbometalation deprotonation (CMD), or electrophilic substitutions (ES).^10^ In Shrock carbenoids, particularly those with early transition metals, the C–H activations occur in the absence of electron transfer between the metal and the substrate,^27^ narrowing the mechanism classically to ES or CMD. Regarding these mechanisms, the nature of their transition states (TS) allows a different classification, which might be concerted [see Scheme 3(a,b)], with ES via an agostic interaction and a σ-complex or a two-step CMD [see Scheme 3(c)]. As the mechanisms of C–H activations depend on many factors and the fact that their TS are difficult to locate experimentally, their mechanisms are still in constant research.^4,28^

**Scheme 3:**
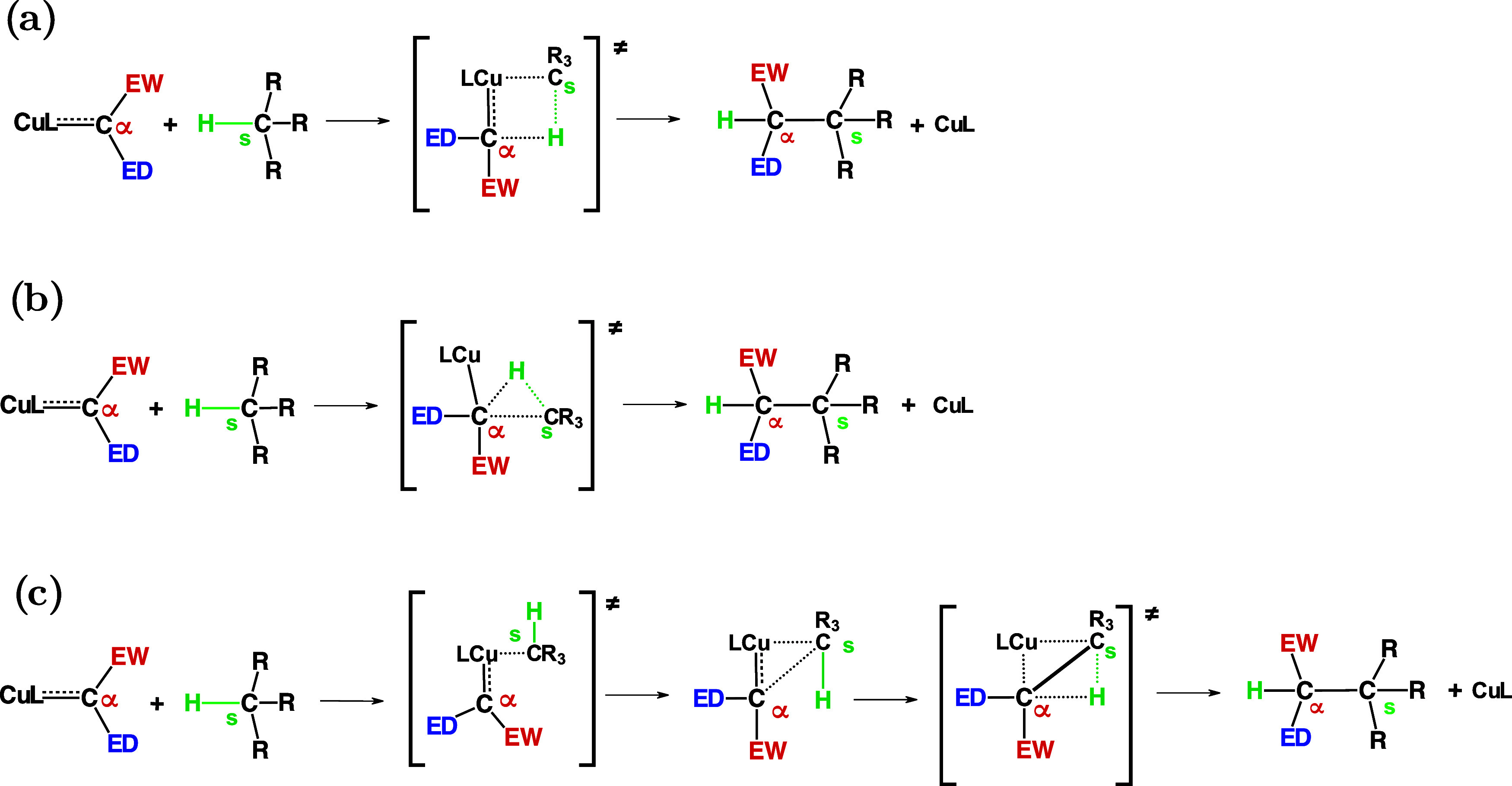
Insertion Stage is Achieved Through (a) ES via an Agostic Complex, (b) ES via a σ-Complex, and (c) a Two-Step CMD, Where L is Its Ligand, Cα Determines the Position of the Reactive Carbon Atom, EWG and EDG are Substituents, and TS Represents the Transition States

In this paper, we will analyze density functional theory (DFT) M06–2X/cc-PVTZ/LANL2DZ calculations on model carbenoid C–H activation and the alkyl insertion into methane **(me)**, ethane **(et)**, propane **(pr)**, and isobutane **(ib)** with Cu­(I) carbenoids substituted with EDG groups OH, CH_3_, or NH_2_, and EWG groups Cl, COOH, and CN as Scheme 3 shows. It is worth noting that Cu is selected based on its low oxidation state, which makes it a suitable electron acceptor, forming highly polar complexes that favor the formation of covalent bonds^29^ which has been used over the years experimentally and theoretically.^25,26,30,31^ The ligand selected in our model is a bidentate 2,2-bipyridine, which is selected to give stability through a chelating effect on the carbenoid Cu. The main goal of this study is to identify the reaction mechanism by the nature of their TS, classifying them as one-step electrophilic substitutions or two-step carbometalation. This procedure will allow us to evaluate the combined effect of these groups on energy barriers and the preference for stepwise or concerted insertion mechanisms, ultimately proposing the most suitable system for C–H activation that is energetically viable and kinetically and thermodynamically more favorable.

This task can be achieved by the analysis of the reaction energies and barriers of each proposed reaction using the reaction force (RF) formalism,^32–34^ activation strain model (ASM),^35–37^ and the energy decomposition analysis (EDA).^38^ To perform the reactivity and selectivity analysis of the carbenoid and substrate, we will use reactivity and selectivity indexes coming from conceptual density functional theory (c-DFT),^39^ such as chemical potential (μ), hardness (η), electrophilicity (ω), and the dual descriptor, [Δ*f*(*r*)]. Along with the electronic properties coming from the natural bond orbitals (NBO)^40,41^ analysis, it is necessary to fully describe the electronic nature of the carbenoids and their influence on the reaction mechanism.

## 2 Theoretical Background

### 2.1 Conceptual DFT (c-DFT)

The reactivity and selectivity analysis was carried out using c-DFT, a branch of DFT^42^ that describes response functions to perturbations either in the number of particles (*N*) or the external potential [*v*(*r*)].^43^ Within this framework, a wide variety of local and global electronic properties describe the ability of molecular systems to undergo chemical changes or modifications in their electron density.^39^

The chemical potential (μ) is defined as the first response of the energy to a perturbation in the number of electrons at a constant external potential. It indicates the escaping tendency of electrons and is associated with the negative of the electronegativity defined by Mulliken.^43^ The molecular hardness (η) is defined as the second perturbation of the energy to the number of electrons and indicates the tendency of an electronic cloud to modify its distribution.^44^

1 $$\mu ={\left(\frac{\partial E}{\partial N}\right)}_{v\left(r\right)},\eta ={\left(\frac{\partial^{2}E}{{\partial N}^{2}}\right)}_{v\left(r\right)}={\left(\frac{\partial \mu }{\partial N}\right)}_{v\left(r\right)}$$

As the energy is discontinuous with *N*, the operational definitions of these indexes are given by the finite difference approximation method, where μ and η can be approximated to ionization potentials (IP) and electron affinities (EA),^45^ and the Koopmans theorem to the energies of the HOMO (ε_H_) and LUMO (ε_L_) orbitals.^46^

2 $$\mu \approx -\frac{\mathrm{IP}+\mathrm{EA}}{2}\approx \frac{\varepsilon_{L}+\varepsilon_{H}}{2},\eta \approx \frac{\mathrm{IP}-\mathrm{EA}}{2}\approx \frac{\varepsilon_{L}-\varepsilon_{H}}{2}$$

Parr et al.^47^ defined the electrophilicity index as the tendency of a system to get saturated with electrons when inserted into a free-electron sea of zero chemical potential, associating this index with the electrophilic nature of a molecular system. It can be calculated from the values of μ and η as follows

3 $$\omega =\frac{\mu^{2}}{2\eta }$$

On the other hand, the local Fukui function^48^ provides a practical way to analyze reactivity at specific atomic sites to study the selectivity of molecular systems. It is defined as the density changes to a perturbation in the external potential

4 $$f\left(r\right)={\left(\frac{\partial^{2}E}{{\partial v\left(r\right)}^{2}}\right)}_{N}={\left(\frac{\partial \rho \left(r\right)}{\partial v\left(r\right)}\right)}_{N}$$

Within the finite difference approximation, the nucleophilic Fukui function *f* ^–^(*r*) and the electrophilic Fukui function *f* ^+^(*r*) can be defined by the left and right derivatives,^49^ which predict nucleophilic and electrophilic sites in a molecular system. It is possible to associate *f* ^–^(*r*) and *f* ^+^(*r*) of an *N*-electron species to the density of the HOMO (ρ_H_(*r*)) and LUMO (ρ_L_(*r*)), by using the frozen core approximation, allowing their evaluation by the valence electrons of a chemical system.^50^

5 $$f^{+}\left(r\right)\approx \rho_{L}\left(r\right),f^{-}\left(r\right)\approx \rho_{H}\left(r\right)$$

To condense this index into atomic domains, the Yang and Mortier approximation^51^ provides an operational definition of *f*_*k*_^–^ and *f*_*k*_^+^ in terms of individual atomic charges as

6 $$f_{k}^{-}\approx q_{k}^{\left(N_{0}\right)}-q_{k}^{\left(N_{0}-1\right)},f_{k}^{+}\approx q_{k}^{\left(N_{0}+1\right)}-q_{k}^{\left(N_{0}\right)}$$

where *q*_*k*_^(*N*_0_)^, *q*_*k*_^(*N*_0_+1)^, and *q*_*k*_^(*N*_0_–1)^ are the atomic charges in the neutral, anionic, and cationic states of the system, respectively.

The local electrophilicity (ω_*k*_),^51^ derived from the condensed Fukui function and the global electrophilicity ω, quantifies the susceptibility of specific sites to electron acceptance. It is given by

7 $$\omega_{k}=\omega f_{k}^{+}$$

Additionally, the dual descriptor Δ*f*(*r*)^52^ allows for the simultaneous identification of nucleophilic and electrophilic regions based on the difference of *f* ^+^(*r*) and *f* ^*–*^(*r*). The operational definition of this index is given by

8 $$\Delta f\left(r\right)=\left[f^{+}\left(r\right)-f^{-}\left(r\right)\right]\approx \left[\rho_{L}\left(r\right)-\rho_{H}\left(r\right)\right]$$

This dual descriptor aids in identifying simultaneously both nucleophilic and electrophilic regions in the molecule, providing valuable information about the molecular sites that are more susceptible to nucleophilic or electrophilic attacks.^52^ This formulation provides an operational framework for predicting the local reactivity and selectivity in chemical reactions. By combining *f*_*k*_^–^, *f*_*k*_^+^, ω and Δ*f*(*r*), it becomes possible to characterize and quantify both nucleophilic and electrophilic regions of a molecule, understanding its local reactivity patterns.^51^

### 2.2 Natural Bond Orbitals (NBO) and Intrinsic Bond Orbitals (IBO) Analysis

The study of electronic structure employs the NBO methodology, which allows for the recovery of the orbital structure responsible for bonding patterns and the electronic distribution of the system based on valence bond theory (VB)^53^ and classical Lewis concepts. NBO represents optimally variational bonds and lone pairs characterized by maximum occupation, where the highest percentage of the total electronic density is in the main NBOs or “Lewis-type” bonds. These Lewis-type NBOs determine the representation of the localized Lewis natural structure wave function. The remaining “non-Lewis” NBOs describe residual ″delocalization effects”, representing deviations from a single localized Lewis structure. Within the NBO methodology, Wiberg bond orders (BO) and populations can be obtained. These quantities, characterized by the average number of shared electron pairs between two atoms, are fundamental for understanding the electronic distribution and bonding strength within the molecule.^54^ In addition, the IBO is employed to characterize the bonding interactions, providing a localized description of bonding by defining the intrinsic orbitals that directly contribute to the bonding and antibonding interactions between atoms. This analysis was used to track the evolution of bond formation, offering insights into the electron density redistribution as bonds are formed or broken throughout the reaction pathway.^55,56^

### 2.3 Reaction Force

To characterize the reaction mechanism, we have defined the reaction force that corresponds to the first negative derivative of the potential energy along the reaction coordinate (ξ)^32–34^

9 $$F\left(\xi \right)=-\left(\frac{dE\left(\xi \right)}{d\xi }\right)$$

This formalism allows us to find critical points that perform a rational partition of the reaction coordinate, where it is possible to quantify the energies associated with different chemical phenomena that are taking part in a chemical process. In a single-step reaction, the reaction force presents two points, a minimum (ξ_min_) and a maximum (ξ_max_), which delimit three regions:^57–59^ The reactants region is defined by *R* → ξ, where most of the structural activations take place, transition state (ξ_min_ → TS → ξ_max_), mostly characterized by electronic rearrangements and the product region (ξ_max_ → *P*) where structural relaxation is present in order to obtain the products of the reaction.

By integration of the reaction force in each region, it is possible to quantify the works (*W*) associated with each reaction zone. *W*_1_ is the activation work associated with the reactant zone, and *W*_2_ is the activation work from ξ_min_ to the TS. The relaxation work is *W*_3_ defined by the TS to ξ_max_, and *W*_4_ is at the products zone. From this partition, we can recover both Δ*E* ^≠^ and Δ*E*° from the contributions of each zone

10 $${\Delta E}^{{}^{\circ}}=W_{1}+W_{2}+W_{3}+W_{4},{\Delta E}^{\neq }=W_{1}+W_{2}$$

Based on the above, *F*(ξ) allows the identification of works associated with both structural and electronic rearrangements in the regions of reactants, transition states, and products and both reaction energies and energy barriers in terms of structural and electronic contributions.^60^

### 2.4 Activation Strain Model (ASM)

This model, also known as the distortion/interaction model, is a fragment-based approach to understanding chemical reactivity in terms of the properties of the original reactants (e.g., steric, rigidity, bonding capability) and the characteristics of reaction mechanisms (e.g., the extent of distortion reactants must undergo).^35–37^ This model can be used to decompose the potential energy along the reaction coordinate, Δ*E*(ξ), and consequently, the reaction barrier, into two key components: the strain energy [Δ*E*_strain_(ξ)] and the interaction energy [Δ*E*_int_(ξ)] between the reactions. This approach can be useful for analyzing and comparing transition state structures, for which the energy barrier is defined as

11 $${\Delta E}^{\neq }={\Delta E}_{\mathrm{strain}}^{\neq }+{\Delta E}_{\mathrm{int}}^{\neq }$$

The strain energy, Δ*E*_strain_^≠^, is the penalty that must be paid to deform individual reactants from their equilibrium structure to the geometry of the transition state, thus, related to the rigidity of the reactants; it is a destabilizing energy (Δ*E*_strain_^≠^ > 0) which contributes to the height of the barrier (Δ*E* ^≠^). Furthermore, Δ*E*_strain_^≠^ can be decomposed into the strain energies of the reacting fragments i.e., A and B into Δ*E*_strain,A_^≠^ and Δ*E*_strain,B_^≠^ at the geometry of the transition state

12 $${\Delta E}_{\mathrm{strain}}^{\neq }={\Delta E}_{\mathrm{strain},A}^{\neq }+{\Delta E}_{\mathrm{strain},B}^{\neq }$$

The interaction energy (Δ*E*_int_^≠^) accounts for the chemical interactions that arise when the two reactants are brought together from infinity to the transition state geometry. This term is directly related to the bonding capabilities and interactions between the deformed reactants at the TS being a stabilizing energy (Δ*E*_int_^≠^ > 0) that counteracts Δ*E*_strain_^≠^.

### 2.5 Energy Decomposition Analysis

The Δ*E*_int_^≠^ at the transition state can be further analyzed using Energy Decomposition Analysis (EDA), which allows for a detailed partitioning of Δ*E*_int_^≠^ into its physical contributions.^38^ In this context, the interaction energy at the TS is decomposed as

13 $${\Delta E}_{\mathrm{int}}^{\neq }={\Delta E}_{\mathrm{elec}}^{\neq }+{\Delta E}_{\mathrm{Pauli}}^{\neq }+{\Delta E}_{\mathrm{oi}}^{\neq }+{\Delta E}_{\mathrm{disp}}^{\neq }$$

where Δ*E*_elec_^≠^ accounts for the electrostatic interactions between the charge distributions of the deformed reactants. This term captures the long-range interactions between the positively and negatively charged regions of the fragments. Δ*E*_Pauli_^≠^ represents the Pauli repulsion, arising from the antisymmetrization of the wave functions of the reactants. It is a short-range repulsive force that arises due to the Pauli exclusion principle, which prevents the occupation of the same quantum state by Fermions. Δ*E*_oi_^≠^ represents orbital interactions that arise from the overlap of molecular orbitals. Finally, Δ*E*_disp_^≠^ accounts for long-range attractive dispersion interactions between the deformed fragments being due to instantaneous dipole–induced dipole interactions.

## 3 Computational Details

A systematic study was carried out at the DFT theory level using the M06–2X functional,^61^ combined with a cc-pVTZ basis set^62^ and a LANL2DZ quasi-relativistic pseudopotential for the Cu atom^63^ to fully optimize the stationary states. The dispersion correction method GD3 was included for the self-consistent field energies and gradients to avoid short-range atomic repulsion interactions. To confirm all our stationary states and to obtain the reaction Gibbs free energies (Δ*G*°) and the barriers (Δ*G* ^≠^), we performed frequency calculations employing analytical second derivatives, including zero-point energy (ZPE) corrections and thermal contributions. The calculations were conducted at 298 K and 1 atm.

We obtained the energies at the reaction coordinate using the intrinsic reaction coordinate methodology (IRC),^64^ considering at least 600 points and a stepsize along the reaction path of 0.03 amu^1/2^Bohr (*Stepsize = 3*), at M06–2X/cc-pVTZ and LanL2DZ. We extracted the geometries of each point at the reaction coordinate and performed single point calculations at the same level of theory, adding GD3 dispersion correction to obtain geometric and electronic properties in our systems.

All quantum mechanical calculations, energies, reaction coordinates, and structural and energetic properties of the molecular systems were performed using the *Gaussian 16 B.01* package.^65^ The analyses related to the wave function, such as the dual descriptor,^52^ were conducted based on the wave function obtained from *Gaussian 16* and analyzed through the *Multiwfn 3.8* program.^66,67^ Additionally, information about populations in the NBO^68,69^ scheme was obtained using the *NBO 7.0* program,^40,41^ available in *Gaussian 16*.^65^ The energy analysis within the context of ASM^35–37^ and EDA^38^ was carried out using the *ADF* program^70^ using the M06–2X/DZP with D3zero dispersion correction.

## 4 Results and Discussion

To ensure the successful activation of the Cs–H bond when it interacts with the carbenoids, it is important to characterize the proposed systems before analyzing the reaction mechanism. In this context, Figure 1(a,b) displays the ten carbenoids considered in this work (**a** to **j**), which will be analyzed in terms of their geometries, intrinsic reactivity from c-DFT and electronic properties to establish a reactivity order in terms of the substitution with EDG and EWG. We have included an unsubstituted system as a reference in the upcoming analysis.

**Figure 1:**
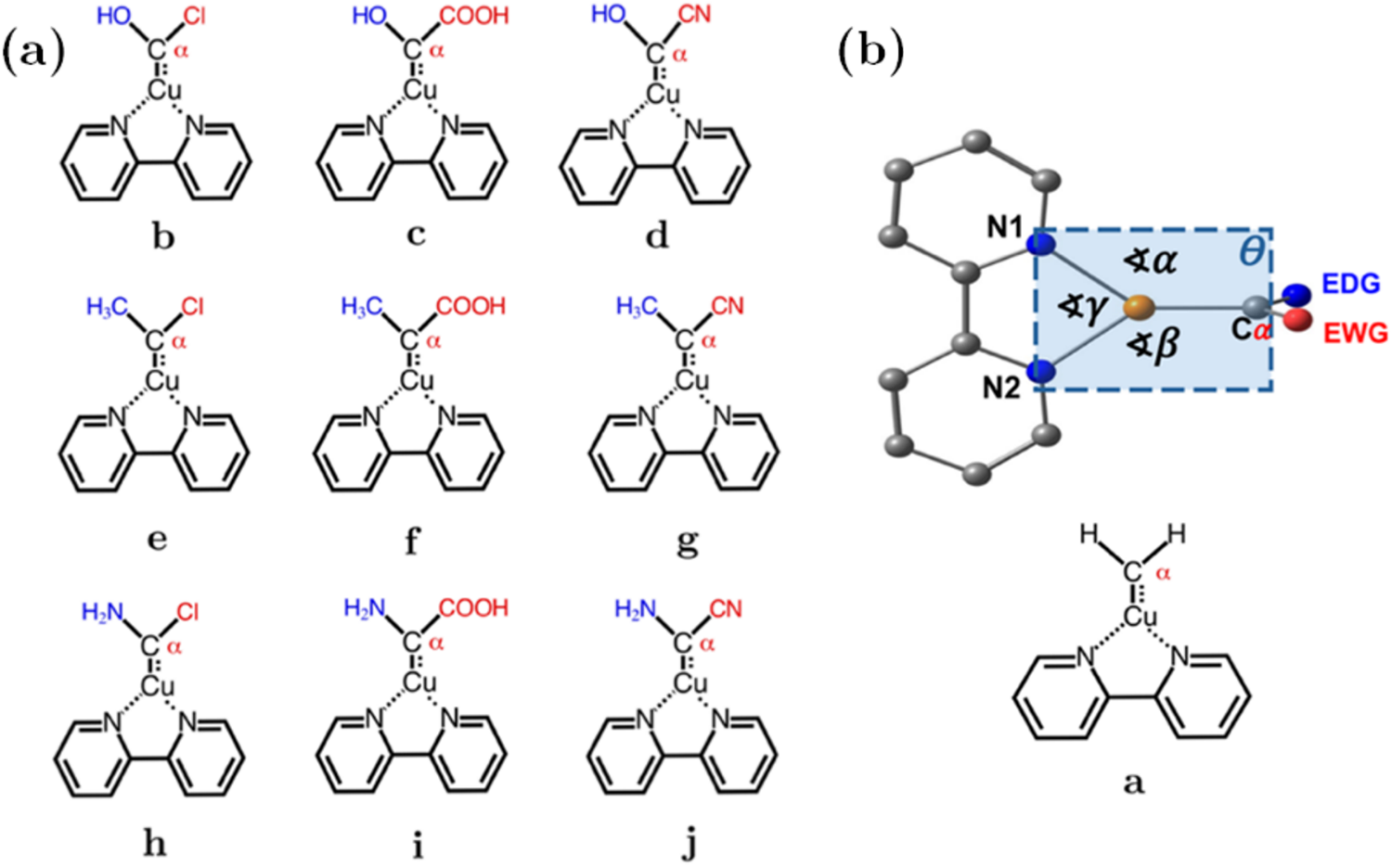
(a) Carbenoids **b** to **j** considered in this study. (b) Scheme of the geometrical parameters discussed in this section, ∠ and θ are the bond and the dihedral angles analyzed and system **a**, considered as reference. Ca is the carbenoid carbon, EDG are represented in blue, and EWG are in red.

### 4.1 Geometrical Parameters

Figure 1(b) displays a scheme representing the geometrical parameters that will be compared between substituted carbenoid, the Cu–N1 (*d*_1_), Cu–N2 (*d*_2_), Cu–Cα (*d*_3_) bonds, the angles formed with Cα, Cu, and the N atoms of the ligand Cα–Cu–N1­(∠α), Cα–Cu–N2 (∠β), N1–Cu–N2 (∠γ), and the dihedral angle Cα–Cu–N1–N2 (θ) located in the plane of the figure. The system **a** exhibits bond distances *d*_1_, *d*_2_, and *d*_3_ of 2.07, 2.07, and 1.89 Å, respectively. It also presents bond angles ∠α, ∠β, and ∠γ of 139.9, 139.9, and 80.2° respectively, and θ = 0°. This system agrees with the trigonal planar geometry, coming from the rigid bpy ligand and the *d* ^10^ electronic configuration of the metal, stabilized by the bpy ligand, as shown in similar studies.^71^ When the carbenoid is substituted with one EDG and one EWG, the bonds *d*_1_, *d*_2_, and *d*_3_ increase to 2.00, 2.11, and 2.11 Å, respectively. Meanwhile, the ∠α, ∠β, and ∠γ values lie within the range of 126.4–140.6, 139.9–153.7, and 78.7–80.1° respectively, with a θ difference not exceeding 3.78°, preserving the planar geometry as shown by Table S1 in the Supporting Information.

### 4.2 NBO Analysis

To understand the nature of the Cu–Cα interaction and how the substitution affects it, we used the Wiberg bond order (BO) to assess the bonds formed by the donor–acceptor interactions. The Cu–Cα BO for **a** was 0.72, which revealed a slightly polarized covalent bond. Among substitutions, the BO decreases, presenting values of **b** = 0.36, **c** = 0.42, **d**=0.40, **e** = 0.42, **f** = 0.45, **g** = 0.47, **h** = 0.33, **i** = 0.36, and **j** = 0.33. These changes can be assessed by second-order perturbation energies [*E*(2)] that quantify the Cu–Cα σ­(*sp* ^2^ → *s*) and π­(*d* → *p*) stabilizing interactions shown in Figure 2.

**Figure 2:**
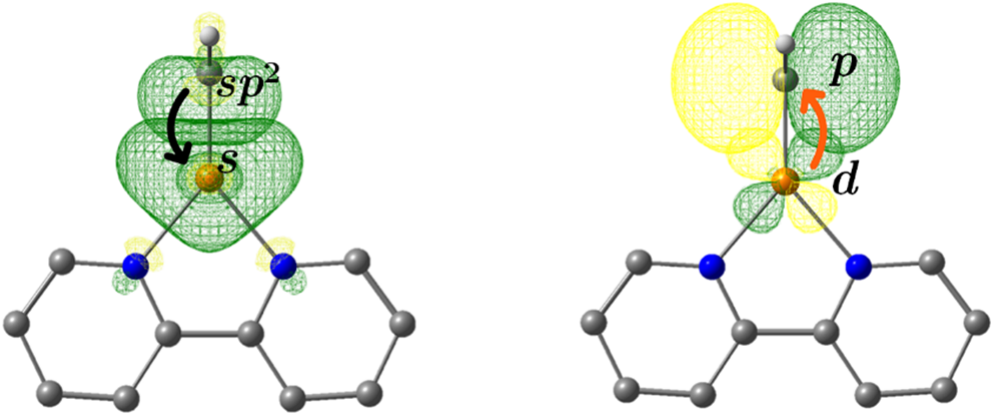
Molecular orbitals given by the NBO analysis for the donor–acceptor interactions between Cu and Cα atoms in carbenoid **a**. Each arrow indicates the direction of the electron charge transfer. All other carbenoids are presented in the supporting material in Figure S1.

Our results shown in Figure 3(a) indicated that carbenoids from **c** to **g** present the same interactions as **a**, they do not have a second *BD* with any of their EDG or EWG, allowing the π­(*d* → *p*) back-donation increasing their *E*(2) and strengthen the Cu–Cα bond. On the contrary **h**, **i**, and **j** carbenoids present the lowest *E*(2) values 74.9, 87.7, and 81.4 kcal/mol, respectively [Figure 3(b)]; these systems are substituted by –NH_2_ an EDG substituent that forms two *BD* with Cα, a σ bond (*sp* ^3^ → *sp*) and π bond (*p* → *p*), not allowing the back-donation observed in **a** [Figure 3(c)]. In the case of **b** (OH/Cl), there is no back-donation from Cu to Cα. It does not form a π bond either because of the interaction with the −OH group, which has a positive mesomeric effect (+M), and the −Cl, with a negative inductive effect (−I), causes their LP to partially occupy the vacant orbital LV of the Cα atom.

**Figure 3:**
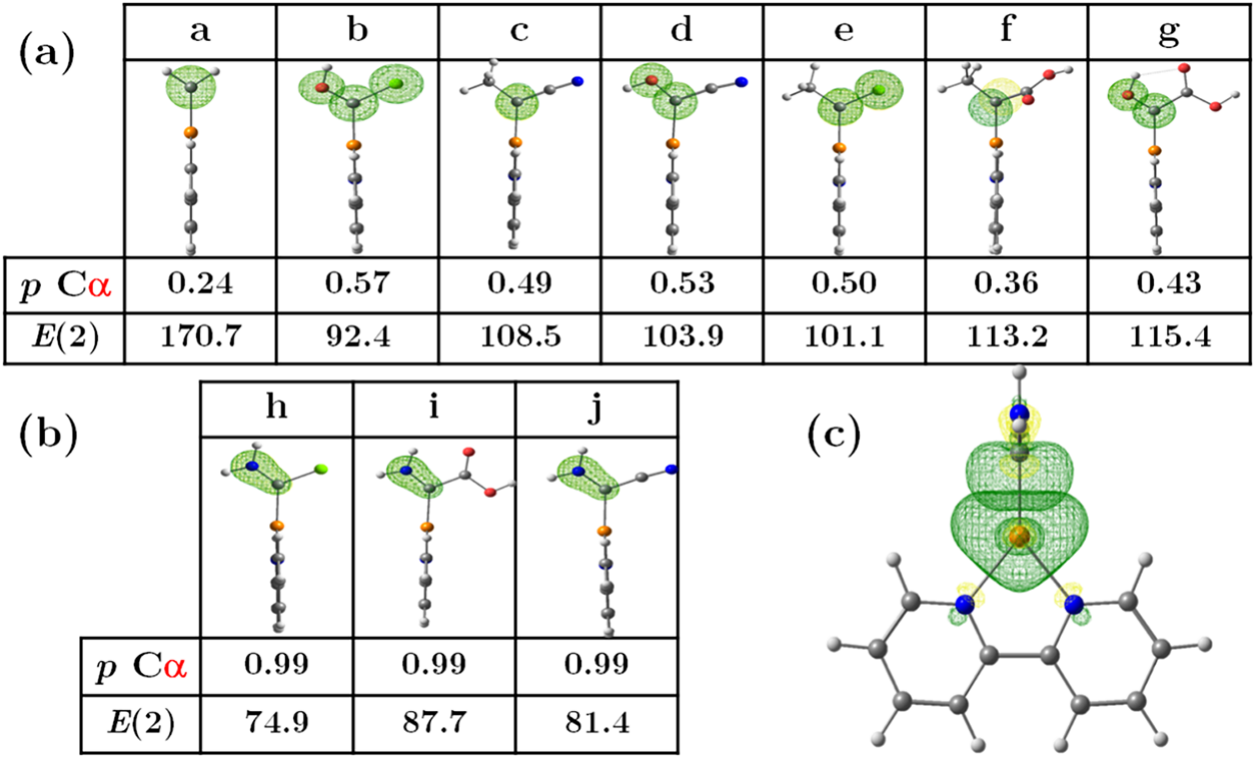
(a) Cα *p* orbitals in carbenoids **a** to **g**, electron occupancies (in |ė|) and stabilization energy values [*E*(2)] of Cu–Cα (kcal/mol) affected by back-donation and donor–acceptor interactions. (b) Cα *p* orbitals in carbenoids **h** to **j**, electron occupancies (in |ė|) and stabilization energy values [*E*(2)] of Cu–Cα (kcal/mol), affected only by EDG interactions. (c) Orbitals of **h** to **j** involved in the Cu–Cα bond and the absence of back-donation.

Overall, our results highlight the critical role of substituent effects in modulating the Cu–Cα bond strength. The presence of EDGs that engage in BD interactions with Cα significantly weakens the Cu–Cα interaction by suppressing back-donation, while EWGs that do not interfere with back-donation enhance bond stability. All of these effects achieve differences in the Cα *p* orbital occupancies, as shown in Figure 3(a,b). Those values range from **a** = 0.24, **b** = 0.57, **c** = 0.49, **d**=0.53, **e** = 0.50, **f** = 0.36, **g** = 0.43, indicating that all of those carbenoids have an available *p* orbital that will be filled in an electrophilic reaction. However, in the case of **h** = 0.99, **i** = 0.99, and **j** = 0.99, the *p* orbital is not available because it is engaged in a π bond with the lone pair of –NH_2_, as previously stated.

### 4.3 c-DFT Intrinsic Reactivity

By analyzing the values of η, ω, and ω_*Cα*_, we can gain insights into how the substituents also affect the intrinsic reactivity of the carbenoid species. According to the *Maximum Hardness Principle* (MHP), which states that chemical systems rearranges themselves to exhibit high hardness, thus lower values of η indicate more reactive systems,^72^ and the *Minimum Electrophilicity Principle* (MEP) were molecules tend to decrease their electrophilicity values, lowering their reactivity,^73^ so high values of ω indicate more reactive systems. For our alkyl insertion reactions, η and ω must lie at intermediate values, ensuring reactivity, control of the reaction, and selectivity of the systems.

In Figure 4, we display η, ω, and ω_Cα_ of the substituted carbenoids (**a**-**j**), where it is observed that these carbenoids follow both MHP^72^ and MEP^74^ principles; when comparing their η and ω values, the hardest system (**h**) shows the lowest electrophilic behavior, and vice versa, the system with the highest electrophilicity (**g**) presents the lowest value of η. Carbenoids **g** and **h** show η values of 118 and 162 kcal/mol respectively and will not be the best suited to activating C–H bonds either highly reactive (**g**) or less reactive (**h**). Also, to ensure the selectivity of each system, it is necessary to explore their electrophilicity. It is well-known that highly electrophilic carbenoids lose selectivity, and these systems are also not the best candidates for an alkyl insertion needing mild electrophilicities. System **g** presents the highest ω of 192 kcal/mol and **h** presents the lowest ω (105 kcal/mol). Consequently, those systems are not well suited for activation.

**Figure 4:**
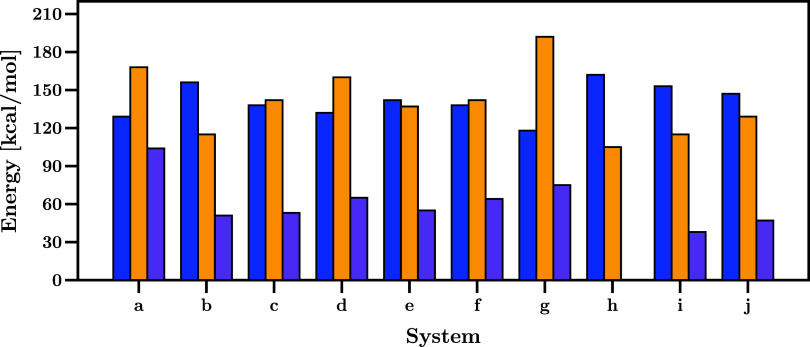
Chemical hardness (η, blue), global electrophilicity (ω, yellow), and local electrophilicity (ω_*Cα*_, purple) of carbenoids **a** to **j**. The graphics information can be found in Table S2.

Analyzing the local electrophilicity at the Cα atom, represented by ω_Cα_, provides further insight into how substituents influence the reactivity of the carbenoids. As is predictable, carbenoid **h** presents no electrophilicity (0 kcal/mol). In contrast, carbenoids such as **a** (104 kcal/mol) and **g** (75 kcal/mol) exhibit a higher local electrophilicity, in agreement with the results of the occupancy of the Cα p orbital. Confirming our previous observations allows us to discard those substitutions from the pool of possible candidates to activate a C–H bond. Given these results, the best candidates for a substitution are those carbenoids with intermediate values such as **c**, **d**, **e**, and **f**. It is also important to remark that these results are consistent with the stability of the previously discussed Cα–Cu bond.

The dual descriptor [Δ*f*(*r*)] was also determined to predict the local reactivity of the carbenoids under study. This index allows us to distinguish areas where electron-donation (nucleophilic) and electron-accepting areas (electrophilic) are located in a chemical system. In Figure 5(a), this descriptor reveals that the electrophilic region is primarily localized at Cα in most carbenoids, except carbenoid **h**. When the alkane molecules are added to the analysis in Figure 5(b), it is observed that the nucleophilic regions are localized at the C–H bonds of each system, indicating a favorable interaction with the electrophilic Cα, ensuring the viability of the reaction.

**Figure 5:**
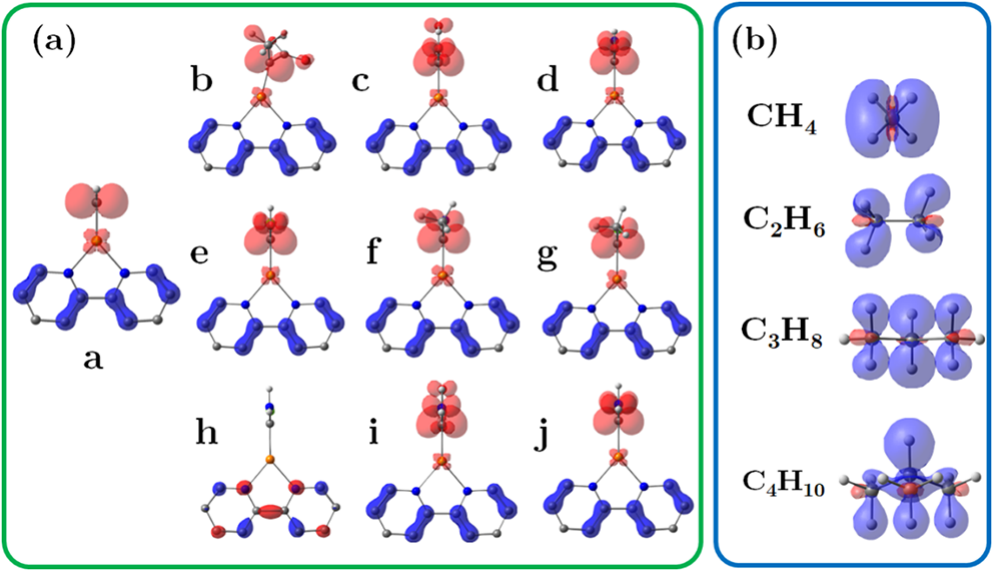
Dual descriptor for (a) the carbenoids and (b) alkane substrates. The blue surface shows the nucleophilic zone, and the red surface represents the electrophilic zone of the molecules.

### 4.4 Reaction Mechanism Analysis

In Scheme 4, we present the mechanism of the insertion reaction of carbenoids with alkyl substrates. According to our results, all reactions proceed by the exact same mechanism, consisting of five stationary states and a single kinetic step that shows the concerted insertion of the carbenoid in the C–H moiety by a σ-complex, which in the scheme is illustrated for simplicity in **a** and **me**. This reaction starts from the R, with both carbenoid and substrate approaching each other to form a reactant complex (RC), then reaching a TS that is characterized by the formation of a σ-complex (Cα–H–Cs) where the electrophilic Cα gets electron density from the Cs–H bond without the participation of the metal. This TS leads to an insertion product (I) and the subsequent recovery of the reactivated CuL catalyst (P). All of the reactions are exergonic with Δ*G*° values ranging from −24.8 to −78.0 kcal/mol.

**Scheme 4:**
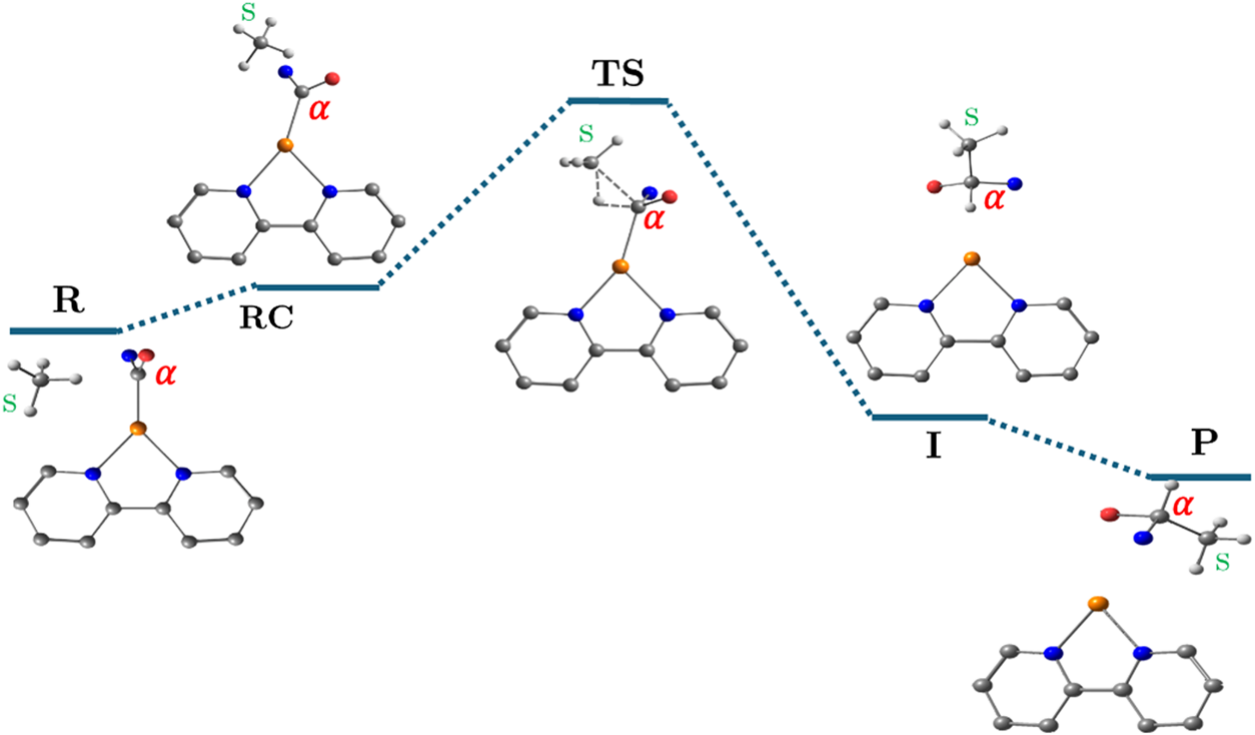
Steps of the Concerted Insertion Reaction via σ-Complex, Involving the Reactants (R), Reactant Complex (RC), Transition State (TS), Intermediate (I), and Products (P)

To understand the feasibility of the reactions, we will analyze the barrier heights of the ten carbenoids with the four considered substrates to establish a reactivity and selectivity order of the combined effects of the reactants. In Figure 6, we present the computed Δ*G* ^≠^ of the insertion reactions of **a** to **j** with methane (**me**), ethane (**et**), propane (**pr**), and isobutane (**ib**), contrasted with ω_Cα_. According to these results, the Δ*G* ^≠^ values can be ordered in three different ranges: low barriers (**a, f, g**), intermediate barriers (**c, d, e)**, and high barriers (**b, i, j, h)**, in good agreement to the Cα–Cu bond orders, and ω_Cα_ with the exception of **d**, due to the lower occupation of the *p* Cα orbital, in contrast to **c** and **e**, augmenting its electrophilicity.

**Figure 6:**
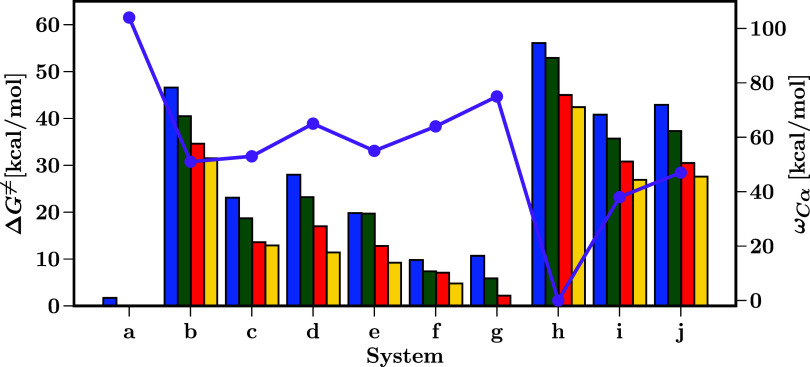
Activation barriers (Δ*G* ^≠^) for the insertion reactions (blue: **me**, green: **et**, red: **pr**, and yellow: **ib**) and the local electrophilicity of Cα (ω_Cα_, purple). The graphic information can be found in Table S3.

Another key relationship was identified between the molecular orbital availability of Cα in isolated carbenoids and their reaction activation barriers. As shown in Figure 3(a,b), the electron occupancy of the unoccupied Cα *p* orbital varies across different systems, influenced by Cu back-donation and the electron-donating (EDG) or electron-withdrawing (EWG) effects as previously discussed in Section 4.2.

#### 4.4.1 IRC and Reaction Force

Following the discussion of the reaction mechanisms is that we will study the energies along the IRC in the kinetic step of the insertion reaction that is from RC, TS, and I, as shown in Figure 7(a). As in carbenoid chemistry, it is important to ensure reactivity and selectivity. In this sense, according to the barriers, all our reactions follow the same trends: **me** > **et** > **pr** > **ib**, where only the substitutions in the Cα are responsible for the reactivity changes among all our systems. To narrow down the discussion, we opted to analyze the carbenoids reacting with **me** that have intermediate barriers (**c**, **d**, and **e**), contrasting their behavior with carbenoids with the lowest (**f**) and highest (**h**) barrier heights.

**Figure 7:**
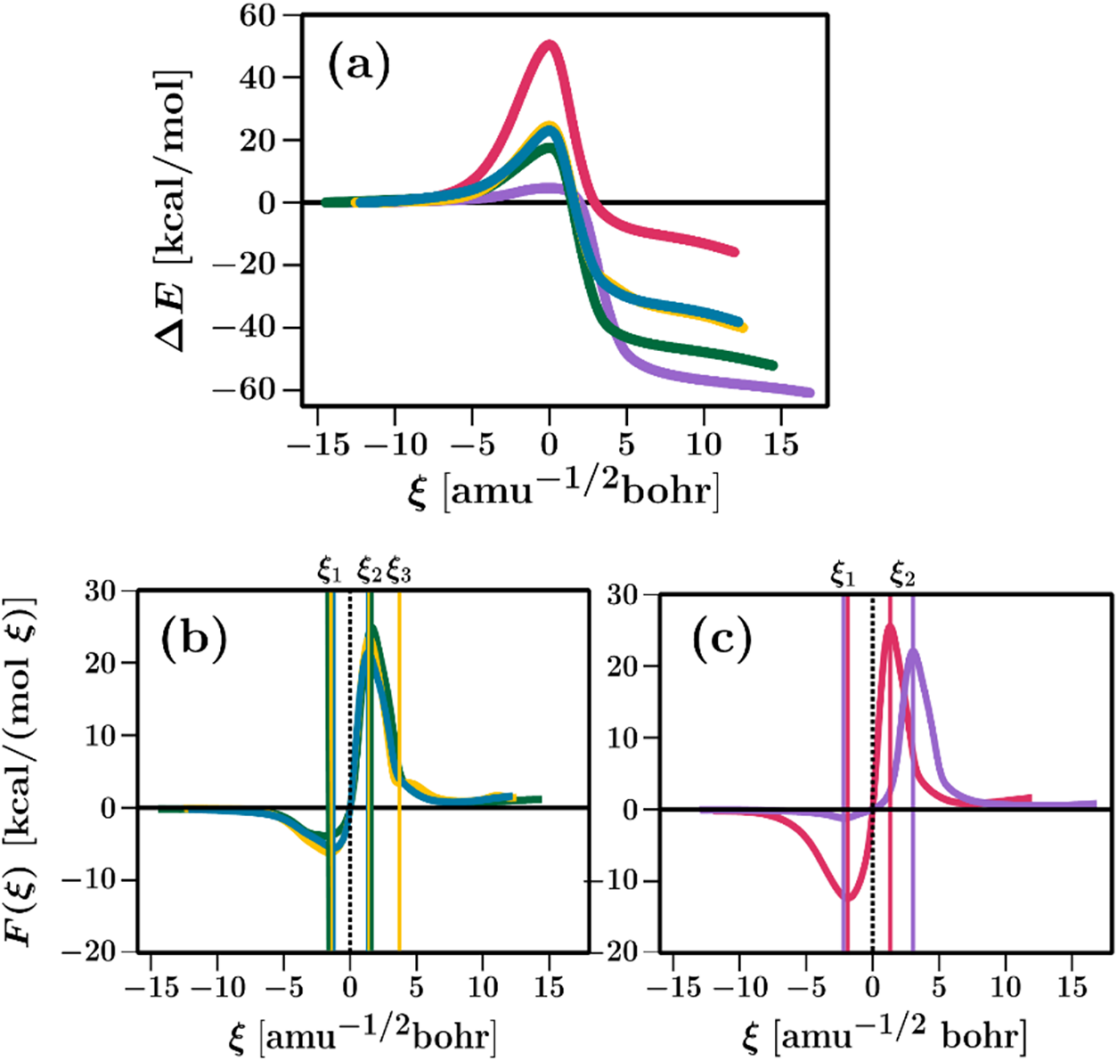
(a) Potential energy profile along the IRC considering the kinetic step of the reaction. (b) Reaction force profiles for the intermediate barriers of carbenoid-**me** reactions, where **e:** green, **d**: yellow, and **c**: cyan. (c) Reaction Force profiles for the lowest and highest barriers where **f:** are purple and **h:** red.

Figure 7(a) shows the profiles obtained along with the IRC in the kinetic step of the reaction for the selected carbenoid systems and their energies in Table 1. They follow the same trends obtained in the Δ*G* ^≠^ values of the overall reactions. From these profiles, we calculated the *F*(ξ) to partition the reaction mechanisms, the key to identifying the regions where chemical properties can activate/deactivate in a reaction path.

**Table 1:** Total Energy (Δ*E*°), Activation Energy (Δ*E* ^≠^), and Obtained Reaction Works (*W*) at the Selected Carbenoid Reactions with **me** ^a^

carbenoid	Δ*E*°	Δ*E* ^≠^	*W*_1_ (%)	*W*_2_ (%)	*W*_3_	*W*_4_
**h** (NH_2_/Cl)	–15.9	50.4	33.8 (67)	16.6 (33)	–20.5	–45.7
**c** (OH/Cl)	–38.1	22.9	18.0 (78)	4.96 (22)	–17.4	–43.7
**d** (OH/COOH)	–39.9	24.5	17.4 (71)	7.10 (29)	–19.5	–44.9
**e** (OH/CN)	–52.0	17.5	12.5 (72)	4.96 (28)	–22.4	–47.1
**f** (CH_3_/Cl)	–60.8	4.63	3.17 (68)	1.47 (32)	–23.1	–42.3

a All reported energies are expressed in kcal/mol.

Figure 7(b) shows *F*(ξ) along the IRC for the intermediate barriers and Figure 7(c) the *F*(ξ) of the lowest and highest barrier reactions. Those figures show two critical points, ξ_1_ and ξ_2_, which defines three regions along the reaction coordinate, where the region (RC → ξ_1_) indicates where the carbenoid and methane are getting close together, consisting mainly in structural rearrangements, and the product zone (ξ_2_ → I) represents the formation of the insertion product and the detachment of Cα–Cs bond from the CuL. In the TS region (ξ_1_ → ξ_2_), most of the electronic activity occurs, such as forming the σ-complex. In the case of **d**, we found an additional critical point (ξ_3_), which although small, corresponds to the rotation of the OH group before the formation of I.

Table 1 shows that the activation (*W*_1_, *W*_2_) and relaxation (*W*_3_, *W*_4_) works along the reaction coordinate. The results show that the energy barriers are dominated mainly by *W*_1_, by 67–78% of the Δ*E* ^≠^ corresponding to structural work. For Δ*E*°, it was found that the highest contribution is attributed to the structural relaxation work *W*_4_.

#### 4.4.2 NBO and IBO Analysis

The Wiberg bond order (BO) analysis was carried out to analyze the electronic evolution of the bonds involved in the TS and the σ-complex formation combined with the partition of the reaction coordinate given by the *F*(ξ). Analyzing the simultaneous bond-breaking and bond-forming events allows us to shed light on the nature of these processes as well as the role of the CuL catalyst.

Figure 8 displays the BO of the species involved in the formation of the σ-complex leading to the formation of the TS in **c** (OH/COOH), **d** (OH/CN), **e** (CH_3_/Cl), **f** (CH_3_/COOH), and **h** (NH_2_/Cl) reactions. These results indicate that the electronic rearrangement occurs mainly in the TS zone, given by the critical points of *F*(ξ). The electronic rearrangement at the TS zone was found to be asynchronous. In **c**, **d**, **e**, and **h**, it starts with the breaking of the Cu–Cα bond in ξ_1_, and this event triggers the formation of Cα–H, Cα–Cs, leading to the σ-complex formation characterized by the crossing of Cα–H, Cα–Cs, and Cs–H BO at the TS. Meanwhile, the electronic rearrangement in **f** is shifted to positive values; the Cu–Cα bond breaks at the TS, a consequence of the back-donation in the Cα–Cu bond that makes the bond to start breaking later in the reaction, thus the formation of the σ-complex at ξ_2_ by the same asynchronous mechanism.

**Figure 8:**
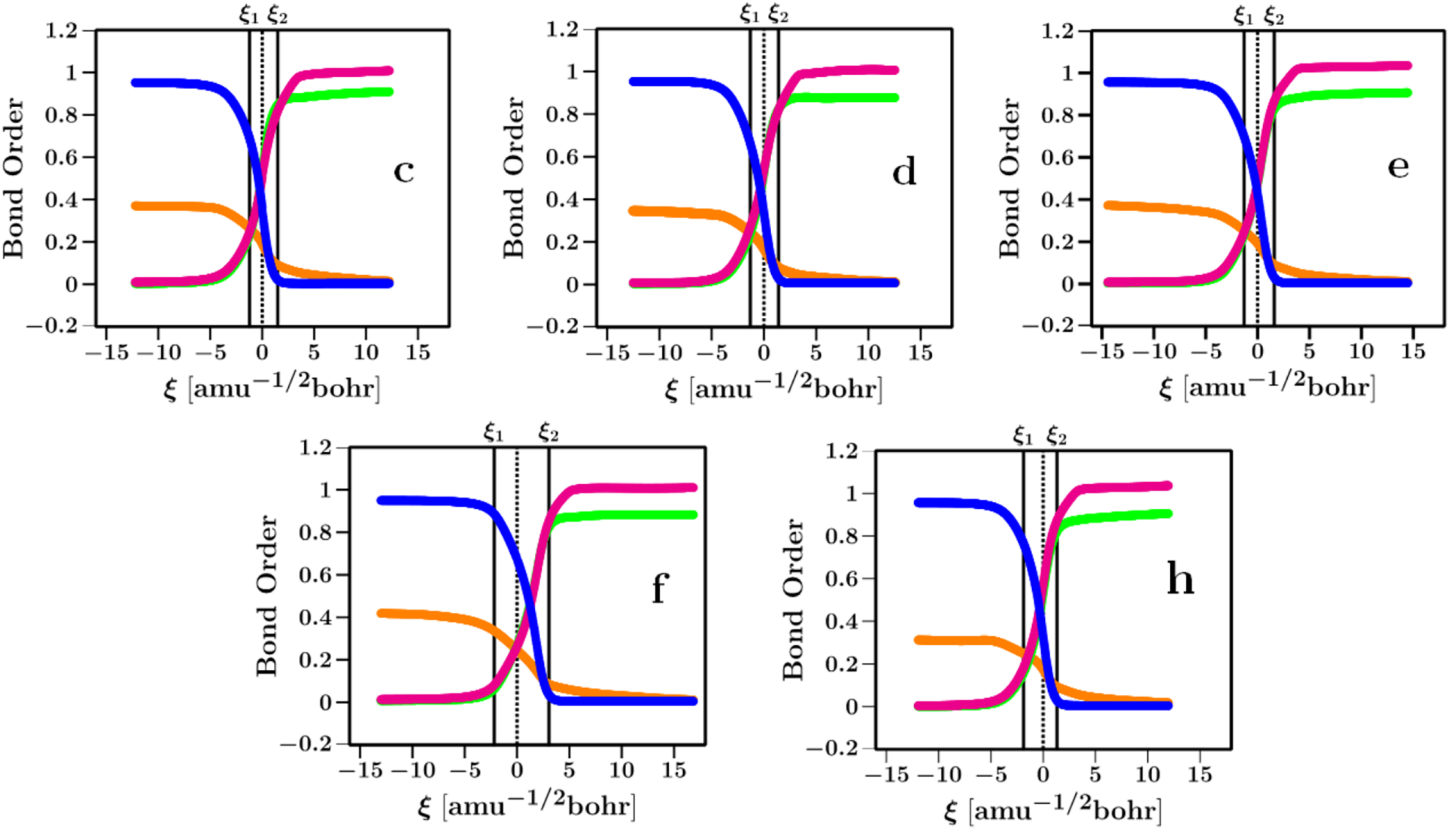
Bond orders of the Cα–Cs (pink), Cα–H (green) bond formations, and Cs–H (blue) and Cu–Cα (orange) bond dissociations. A solid line marks the critical points (ξ_1_ and ξ_2_), and a dotted line marks the TS.

To complement the BO analysis and to understand the electronic factors involved, we present an NBO-IBO analysis considering the stationary states (RC, TS, and I) of the reaction as well as the critical points (ξ_1_ and ξ_2_) coming from the *F*(ξ) in three selected reactions: **d**, **f**, and **h**, with thee last two systems chosen as a comparison of lowest and highest barriers, as shown in Figure 9. As previously discussed, the Cα → Cu bond of the RC originates from the electron donation of a *sp* ^2^ LP of the Cα to an *s* LV orbital of Cu. In ξ_1_, the partially occupied *p* orbital of Cα begins interacting with the *sp* ^3^ Cs–H bond. The extent of the back-donations and the unoccupied-partially occupied *p* orbital are key to determine the reactivity of their systems, even though the carbenoid structure keeps the *sp* ^2^ geometry, the carbon atom must not fully form a double bond with the metal, as it starts behaving like a carbene as seen for **a**. This list of carbenoid reactions have donor and acceptor groups that allow the available *p* orbital of the Cα to act as an acceptor, behaving more like a *sp* ^3^ atom as classic carbenoids.^15^ In the case of **h**, the interaction is from the *sp* ^3^ Cs–H to the *p*-orbital of Cα-NH_2_, which is the reason for its lowest electrophilicity and the high barrier of the reaction. At the TS, the σ-complex is formed with the density of the *sp* ^3^ Cs–H bond and the *sp* ^2^ bond of Cα–Cu.

**Figure 9:**
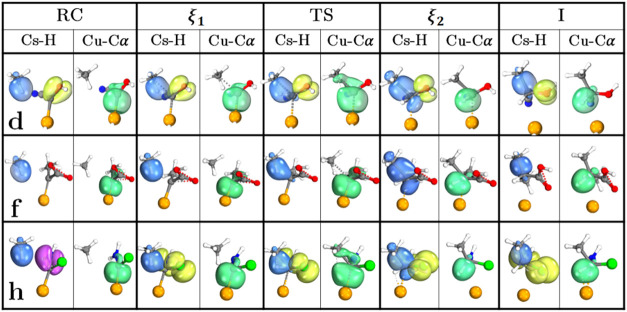
NBO and IBO analysis of key donor–acceptor interactions and bond evolution in systems **d** (OH/CN), **f** (CH_3_/COOH), and **h** (NH_2_/Cl). Blue: Cs–H σ bond transforms into the Cα–Cs σ bond. Yellow: lone pairs on O, N, and Cl. Purple: N–Cα π bond, Green: Cα → Cu donor–acceptor bond transforms into the Cα–H σ bond.

As mentioned before, the extent of the back-donation determines the strength of the Cα-Cu bond, which, in the case of **f** (lowest barrier), causes the formation of the σ-complex to be later than **d** and **h**. At ξ_2_, the Cα–Cu density is used to form the new *sp* ^3^ Cα-H σ bond and *sp* ^3^ Cα-Cs bond, changing the Cα geometry from planar to tetrahedral. The insertion product is fully formed at the zone I, and the Cu catalyst starts leaving the reacting site.

#### 4.4.3 ASM and EDA Analysis

To better understand and unveil the differences in Δ*E* ^≠^ of the reactions selected in Section 4.4.1.; we have calculated the decomposition of the barriers into their contributions using the ASM values displayed in Figure 10(a). This analysis revealed that the strain energy (Δ*E*_strain_^≠^), associated with the deformation of the molecules required to reach the TS, is the primary factor contributing to Δ*E* ^≠^. Whereas the interaction energy (Δ*E*_int_^≠^) counteracts this effect, stabilizing the barriers, the intermediate barriers had larger interaction energies that follow the barrier tendencies. Whereas for **h** (high barrier) and **f** (low barrier), their Δ*E*_int_^≠^ is identical (5.7 kcal/mol).

**Figure 10:**
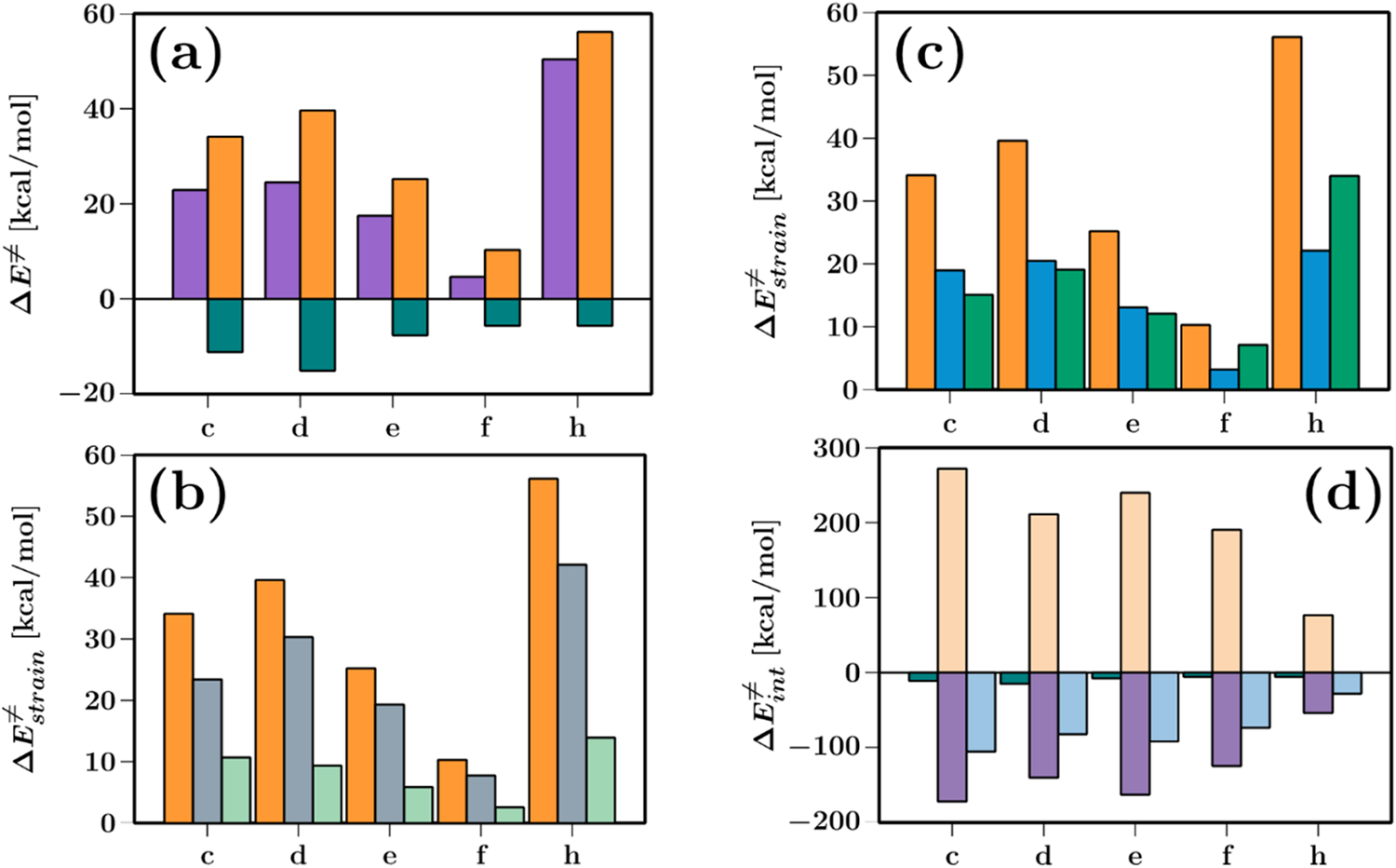
(a) Energy decomposition of Δ*E* ^≠^ (purple) into Δ*E*_strain_^≠^ (orange) and Δ*E*_int_^≠^ (teal). (b) Δ*E*_strain_^≠^ (orange) decomposition into their strain works according to the reaction force critical points, *W*_1,str_ (light green) and *W*_2,str_ (gray). (c) Δ*E*_strain_^≠^ (orange) decomposition into the reacting molecules Δ*E*_str,me_^≠^ (**me**, blue) and Δ*E*_str,carb_^≠^ (carbenoid, green). (d) Δ*E*_int_^≠^ (teal) decomposed into Δ*E*_Pauli_^≠^ (white), Δ*E*_oi_^≠^ (light purple) and Δ*E*_elec_^≠^ (light blue). All values listed are in kcal/mol. All of the graphics information can be found in Table S4.

In order to deepen the analysis, we calculated the *W*_1_ and *W*_2_ contributions to Δ*E*_strain_^≠^ and plotted them in Figure 10(b). *W*_1,str_ corresponds to the strain work related to the approach of the reactant species until reaching ξ_1_. In all reactions, this work is around 35% of the strain energy of Δ*E* ^≠^ because the orbitals involved at the reaction are not close enough to present repulsions between them (see Figure 9). However, *W*_2,str_ presents the highest contribution to Δ*E*_strain_^≠^ because the reacting orbital repulsion strains the systems to rearrange the electron densities after ξ_1_; thus, the breaking of Cα-Cu triggers the formation of the σ-complex, which forms the I after the ξ_2_.

In Figure 10(c), we show the decomposition of Δ*E*_strain_^≠^ into the contributions of the interacting molecules carbenoid (Δ*E*_str,carb_^≠^) and methyl (Δ*E*_str,me_^≠^). The figure shows that the reactions with an intermediate barrier (**c**, **d** and **e**) presented an even degree of deformation of both Δ*E*_str,me_^≠^ and Δ*E*_str,carb_^≠^ contributing uniformly to the strain energy, being Δ*E*_str,me_^≠^ slightly higher. Meanwhile, in reactions **f** and **h**, the carbenoid (Δ*E*_str,carb_^≠^) contributed around 69 and 61%, respectively. This behavior is attributed in **c**, **d**, and **e** to the availability of the Cα *p* orbital to react with the Cs–H of me, lowering the impact of the approaching of this system to start immediately breaking the Cα-Cu bond. In the case of **f**, the **me** has to trigger the Cα–Cu bond breaking, which has more electronic density coming from Cu → Cα back-donation, adding more destabilizing energy to Δ*E*_strain_^≠^. For **h**, the behavior is quite similar. In this case, the nature of the strain comes from breaking the π bond between Cα-NH_2_, which, as mentioned earlier, has an occupied *p* Cα orbital, responsible for the highest barriers.

To understand the physical nature of the Δ*E* ^≠^, we can complement the study using the EDA analysis to determine the nature of the Δ*E*_int_^≠^, and its contributions from Pauli repulsion (Δ*E*_Pauli_^≠^), orbital interaction (Δ*E*_oi_^≠^), and electrostatic potential energy (Δ*E*_elec_^≠^). This decomposition helps to elucidate the relation of steric, orbital, and electrostatic interactions into reaction barriers. This analysis makes it possible to recognize the driving forces coming from the reacting fragments.

Figure 10(d) presents the total values for these terms; the dominant contribution comes mostly from Δ*E*_Pauli_^≠^, attributed to the steric hindrance of the orbitals at the TS as the molecules approach one another in agreement with the IBO analysis, representing a destabilizing term. Δ*E*_oi_^≠^ is the stabilizing term that follows contributing mostly of the Δ*E*_int_^≠^ due to the reorganizing of the orbitals in order to form the σ-complex. Additionally, Δ*E*_elec_^≠^ contributed less stabilizing energy, stemming from classic Coulombic interactions coming from electron transfer and the formation of new bonds. Note that the dispersion contribution will not be accounted for since it is negligible.

From Figure 10(a), **f** and **h** presented identical values of Δ*E*_int_^≠^, and the decomposition given by EDA indicated that they differ in their Δ*E*_Pauli_^≠^, Δ*E*_oi_^≠^, and Δ*E*_elec_^≠^, with **h** being **h** the system that presented the lowest energy associated with steric, orbital, and electrostatic interactions. The occupancy of the *p* Cα makes the reaction mainly driven by strain energy associated with the deformation of the system, opposing to **f** that presents Δ*E*_Pauli_^≠^, Δ*E*_oi_^≠^ and Δ*E*_elec_^≠^ contributions similar to **d**. In the case of the intermediate systems, these present their decomposition values in the same proportion as their barrier energies. Nevertheless, this analysis reveals that all reactions present the same intrinsic interplay of physical terms regardless of Δ*E* ^≠^.

## 5 Conclusions

This theoretical DFT M06–2X/cc-PVTZ/LANL2DZ study on C–H activation reactions used model Cu­(I) carbenoids substituted with EDG (OH, CH_3_, and NH_2_) and EWG (Cl, COOH, and CN). In particular, we calculated their insertion reactions into methane (**me**), ethane (**et**), propane (**pr**), and isobutane (**ib**) to get insights into the catalytic reaction mechanism of the activation of alkane C–H bonds by copper­(I) carbenoids. We found that the carbenoids substituted with the OH/COOH, OH/CN, and CH_3_/Cl showed optimal local electrophilicity, hardness, and intermediate barrier heights, making them highly suitable for selective alkyl insertions. The C–H bonds of the substrates were found to be their nucleophilic counterparts, participating in achieving the activation and insertion of the carbenoid.

All reactions correspond to electrophilic substitutions, with five stationary states and a single barrier characterized by the formation of a σ-complex between the carbenoid carbon and the C–H-activated bond coming from the substrate. The interplay of Cu back-donation and occupation of the *p* Cα LV orbital was identified as a key factor, directly affecting the electrophilicity and reaction barriers. To achieve an intermediate barrier, the *p* Cα orbital needs to be partially filled and accept the electron of the substrate; the presence of EDGs that engage in BD interactions with Cα significantly weakens the Cu–Cα interaction by suppressing back-donation, while EWGs that do not interfere with back-donation enhance bond stability. Strong EDGs such as –NH_2_ severely decreased electrophilicity and increased activation barriers, due to the complete occupation of the Cα *p* orbital with the electron pair of the N.

The analysis employing c-DFT, *F*(ξ) formalism, and NBO along the reaction mechanism, the formation of the σ-complex at the TS zone defined by the critical points of *F*(ξ), the electronic rearrangement of the partially occupied *p* orbital of Cα, the *sp* ^3^ Cs–H, *sp* ^2^ *-s* Cu–Cα, and *sp* ^3^ Cα-H σ bonds is asynchronous in nature. The approaching *sp* ^3^ Cs–H weakens the Cu–Cα that breaks in ξ_1_, triggering the formation of *sp* ^3^ Cα-H and *sp* ^3^ Cα–Cs, leading to the σ-complex formation at the position of the TS. The Cu–Cα back-donation, while widening the TS zone, delays the breaking of the bond, and the complex is formed at the relaxation of the system, between the TS position and ξ_2_.

ASM and EDA indicated that the most important contribution to the barrier is given by the Δ*E*_strain_^≠^, specifically, the *W*_2,str_ associated with the approaching of Cs–H that starts dissociating Cu–Cα as a consequence. In addition, the contributions of Δ*E*_strain_^≠^ from the carbenoid and the substrates are higher in the systems with higher barriers (high *p* Cα occupancy) and lower barriers (high back-donation) that is confirmed by the values of Δ*E*_Pauli_^≠^, the destabilizing term that contributes primarily to Δ*E*_int_^≠^, the only stabilizing energy that is implied to the reorganization of the orbitals in order to form the σ-complex is Δ*E*_oi_^≠^, finding that these energies have the highest influence in the systems of lower and intermediate barriers.

## Supplementary Material



## Notes

Geometrical parameters and natural charges (Table S1); molecular orbitals from NBO analysis (Figure S1); global and local reactivity indexes (Table S2); Gibbs free energy barriers for carbenoid insertions (Table S3); decomposition of activation energies into strain and interaction contributions (Table S4); and Cartesian coordinates of reactants, transition states, products and substrates (Table S5) (PDF)
