# Supplementary material for: Theoretical Study of Cu Carbenoids in C–H Activation Reactions: The Interplay between Metal Back-Donation and Electrophilicity of the Carbon
Source: J Phys Chem A. 2025 Jun 10;129(24):5313–25. doi: 10.1021/acs.jpca.5c02784 (PMC12186614; doi:10.1021/acs.jpca.5c02784)
Supplement: Supplementary file 1 [file jp5c02784_si_001.pdf]

Theoretical Study of Cu Carbenoids in C-H Activation Reactions: The Interplay between the Metal Back-Donation and the Electrophilicity of the Carbon.

Sasha Gazzari-Jara[a] and Barbara Herrera[a]*

a. QTC, Escuela de Química, Facultad de Química y de Farmacia, Pontificia Universidad Católica de Chile, Av. Vicuña Mackenna 4860, Macul, Santiago, Chile. 7820436.

*bherrera@uc.cl

Contents in this document

Figure S1: Molecular orbitals from NBO analysis.

Table S1: Geometrical parameters and natural charges.

Table S2: Global and local reactivity indexes.

Table S3: Gibbs free energy barriers for carbenoid insertions.

Table S4: Decomposition of activation energies into strain and interaction contributions.

Table S5: Cartesian coordinates of reactants, transition states, products and substrates

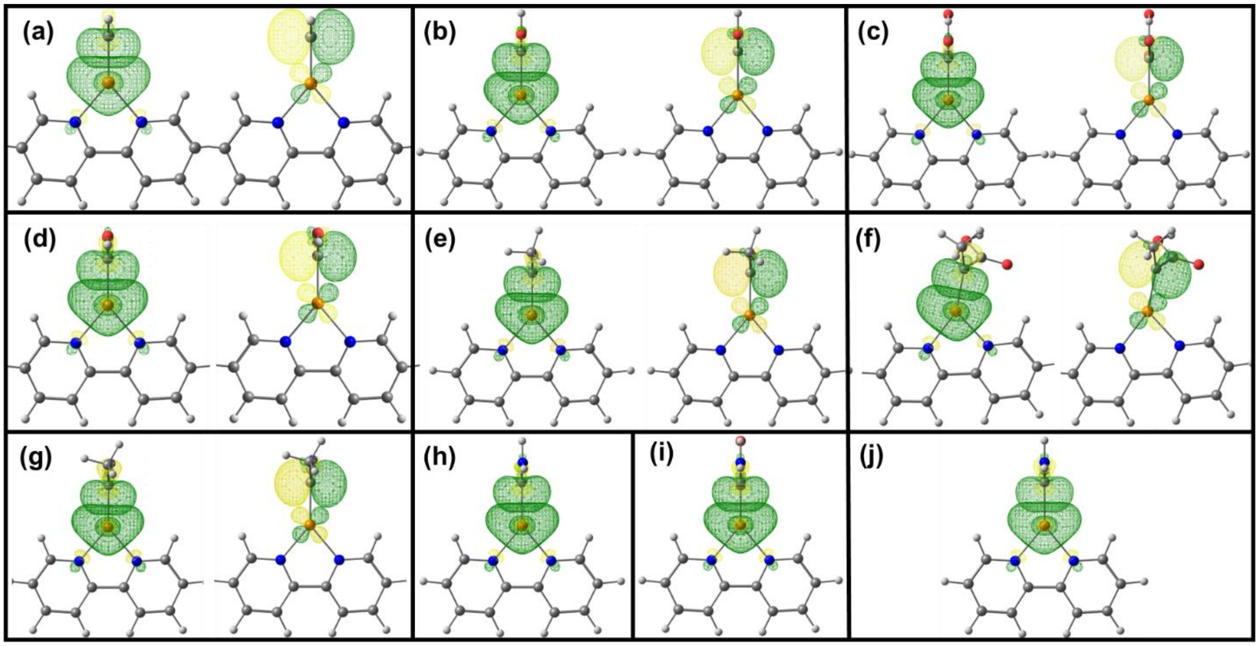

Figure S1. Molecular orbitals given by the NBO analysis for the donor-acceptor interactions between Cu and Cα atoms in all the carbenoids.

Table S1. Geometrical parameters and natural charges for all carbenoids.

Systems	d_1	d_2	d_3	$\angle\alpha$	$\angle\beta$	$\angle\gamma$	θ
**a(H/H)**	2.07	2.07	1.89	139.9	139.9	80.2	0.01
**b(OH/Cl)**	2.09	2.09	1.99	140.2	140.2	79.6	1.01
**c(OH/COOH)**	2.10	2.10	1.97	140.3	140.3	79.3	0.65
**d(OH/CN)**	2.09	2.09	1.97	140.0	140.0	79.9	1.61
**e(CH_3/Cl)**	2.10	2.09	1.96	139.5	141.2	79.3	0.53
**f(CH_3/COOH)**	2.09	2.07	1.94	126.4	153.7	79.9	1.00
**g(CH_3/CN)**	2.08	2.08	1.94	139.0	140.9	80.1	1.25
**h(NH_2/Cl)**	2.11	2.11	2.00	140.5	140.5	78.9	3.11
**i(NH_2/COOH)**	2.11	2.11	1.99	140.6	140.7	78.7	1.61
**j(NH_2/CN)**	2.10	2.10	2.00	140.3	140.3	79.3	3.78

Table S2. Global reactivity indexes (chemical hardness and global electrophilicity) and the local reactivity index (local electrophilicity) for all carbenoids.

Systems	η	ω	$\omega C\alpha$
**a(H/H)**	129	168	104
**b(OH/Cl)**	156	115	51
**c(OH/COOH)**	138	142	53
**d(OH/CN)**	132	160	65
**e(CH_3/Cl)**	142	137	55
**f(CH_3/COOH)**	138	142	64
**g(CH_3/CN)**	118	192	75
**h(NH_2/Cl)**	162	105	0
**i(NH_2/COOH)**	153	115	38
**j(NH_2/CN)**	147	129	47

Table S3. Gibbs free energy barrier ($\Delta G^{\neq}$) for the insertion of the carbenoids in the selected alkyl molecules. $\Delta G^{\neq}$ values are in kcal/mol.

Systems	$\Delta G^{\neq}$***me***	$\Delta G^{\neq}$***et***	$\Delta G^{\neq}$***pr***	$\Delta G^{\neq}$***ib***
**a(H/H)**	1.71	0.00	0.00	0.00
**b(OH/Cl)**	46.6	40.5	34.6	31.5
**c(OH/COOH)**	23.1	18.7	13.6	12.9
**d(OH/CN)**	28.0	23.2	17.0	11.4
**e(CH_3/Cl)**	19.8	19.7	12.8	9.20
**f(CH_3/COOH)**	9.80	7.42	7.10	4.82
**g(CH_3/CN)**	10.7	5.90	2.19	0.00
**h(NH_2/Cl)**	56.1	52.9	45.2	42.4
**i(NH_2/COOH)**	40.8	35.6	30.8	26.9
**j(NH_2/CN)**	42.9	37.3	30.5	27.6

Table S4. $\Delta E^{\neq}$ decomposition into strain and interaction contributions with their corresponding decompositions: $\Delta E^{\neq}_{strain}$ into its corresponding works ($W_{1,str}$ and W_{2str}). and fragment contributions ($\Delta E^{\neq}_{str,me}$ and $\Delta E^{\neq}_{str,carb}$). $\Delta E^{\neq}_{int}$ into their physical contributions ($\Delta E^{\neq}_{Pauli}$, $\Delta E^{\neq}_{oi}$ and $\Delta E^{\neq}_{elec}$). All values are in kcal/mol.

System	$\Delta E^{\neq}_{strain}$	$\Delta E^{\neq}_{int}$	$W_{1,str}$	$W_{2,str}$	$\Delta E^{\neq}_{str,me}$	$\Delta E^{\neq}_{str,carb}$	$\Delta E^{\neq}_{Pauli}$	$\Delta E^{\neq}_{oi}$	$\Delta E^{\neq}_{elec}$
**c(OH/COOH)**	34.1	-11.2	10.7	23.4	19.0	15.1	211.3	-140.2	-82.3
**d(OH/CN)**	39.6	-15.1	9.37	30.3	20.5	19.1	239.8	-162.9	-91.9
**e(CH_3/Cl)**	25.2	-7.67	5.86	19.3	13.1	12.1	190.5	-124.6	-73.6
**f(CH_3/COOH)**	10.3	-5.67	2.56	7.74	3.19	7.11	76.5	-53.9	-28.2
**h(NH_2/Cl)**	56.1	-5.66	13.9	42.1	22.1	34.0	272.1	-172.1	-105.6

Table S5. Cartesian coordinates (XYZ) for all studied structures.

Carbenoid a			
29	0.000225000	1.744029000	-0.000112000
6	0.000149000	3.631753000	-0.000403000
1	0.000454000	4.282104000	-0.879077000
1	-0.000342000	4.280587000	0.879431000
6	-3.479484000	-0.867218000	-0.000043000
6	0.744343000	-1.043730000	0.000076000
6	1.499502000	-2.207432000	-0.000732000
6	2.882290000	-2.116021000	-0.000689000
6	3.479324000	-0.867614000	0.000156000
6	2.660662000	0.248501000	0.000836000
7	1.331355000	0.163350000	0.000771000
1	1.025497000	-3.176003000	-0.001514000
1	3.484307000	-3.013871000	-0.001335000
1	4.552605000	-0.752244000	0.000266000
1	3.078301000	1.245923000	0.001469000
6	-0.744550000	-1.043606000	0.000118000
6	-1.499828000	-2.207238000	0.000421000
6	-2.882595000	-2.115706000	0.000325000
6	-2.660713000	0.248802000	-0.000240000
7	-1.331402000	0.163576000	-0.000168000
1	-1.025865000	-3.175830000	0.000834000
1	-3.484727000	-3.013478000	0.000555000
1	-4.552752000	-0.751727000	-0.000144000
1	-3.078264000	1.246264000	-0.000433000
Carbenoid b			
29	-0.969536000	-0.000045000	0.196931000
6	-2.949863000	-0.000073000	0.337945000
6	1.676591000	3.482427000	-0.020189000
6	1.832662000	-0.745796000	-0.034145000
6	2.998010000	-1.494387000	-0.129912000
6	2.917023000	-2.877400000	-0.122772000
6	1.676754000	-3.482375000	-0.020268000
6	0.560390000	-2.669209000	0.071391000
7	0.634355000	-1.339654000	0.064607000
1	3.960786000	-1.015635000	-0.209222000
1	3.816179000	-3.472912000	-0.196652000
1	1.568460000	-4.556391000	-0.010819000
1	-0.430999000	-3.094078000	0.153182000
6	1.832626000	0.745856000	-0.034137000
6	2.997924000	1.494503000	-0.130072000
6	2.916873000	2.877512000	-0.122894000
6	0.560278000	2.669206000	0.071606000
7	0.634304000	1.339655000	0.064784000
1	3.960708000	1.015798000	-0.209566000
1	3.815989000	3.473068000	-0.196907000
1	1.568248000	4.556437000	-0.010699000
1	-0.431120000	3.094027000	0.153546000
8	-3.615560000	-0.000148000	1.429620000
1	-4.587312000	-0.000290000	1.320575000
17	-3.952713000	0.000052000	-1.061556000
Carbenoid c			
29	0.775299000	-0.000220000	-0.307746000
6	2.730813000	-0.000335000	-0.539455000
6	-1.859850000	3.481459000	0.016421000
6	-2.018223000	-0.745418000	0.048100000
6	-3.177519000	-1.495226000	0.194290000
6	-3.095601000	-2.878072000	0.178459000
6	-1.860676000	-3.481213000	0.016810000
6	-0.750233000	-2.666527000	-0.121965000
7	-0.824957000	-1.337179000	-0.106045000
1	-4.136077000	-1.017331000	0.318183000
1	-3.989883000	-3.474971000	0.290468000
1	-1.752244000	-4.555082000	-0.003082000
1	0.236651000	-3.089751000	-0.252063000
6	-2.018044000	0.745705000	0.048041000
6	-3.177093000	1.495807000	0.194686000
6	-3.094847000	2.878632000	0.178683000
6	-0.749658000	2.666491000	-0.122716000
7	-0.824696000	1.337163000	-0.106625000
1	-4.135700000	1.018156000	0.319137000
1	-3.988932000	3.475757000	0.291053000
1	-1.751169000	4.555300000	-0.003636000
1	0.237274000	3.089463000	-0.253265000
8	3.379347000	-0.000668000	-1.630856000
1	4.357453000	-0.000492000	-1.454037000
6	3.759941000	0.000022000	0.617426000
8	4.935514000	-0.000139000	0.381535000
8	3.184264000	0.000417000	1.800587000
1	3.859460000	0.000606000	2.497751000

Carbenoid d			
29	1.066129000	-0.000197000	-0.209124000
6	3.033502000	-0.000335000	-0.345759000
6	-1.564794000	3.482406000	0.029952000
6	-1.724064000	-0.745423000	0.043030000
6	-2.887460000	-1.494864000	0.150511000
6	-2.805247000	-2.877908000	0.143914000
6	-1.565592000	-3.482156000	0.030263000
6	-0.450602000	-2.668732000	-0.072952000
7	-0.525986000	-1.338950000	-0.066985000
1	-3.849694000	-1.016589000	0.238937000
1	-3.703120000	-3.474100000	0.227213000
1	-1.456514000	-4.556085000	0.021433000
1	0.540054000	-3.093295000	-0.163318000
6	-1.723892000	0.745712000	0.042982000
6	-2.887079000	1.495428000	0.150810000
6	-2.804549000	2.878453000	0.144076000
6	-0.450023000	2.668718000	-0.073541000
7	-0.525711000	1.338954000	-0.067438000
1	-3.849387000	1.017381000	0.239666000
1	-3.702255000	3.474858000	0.227648000
1	-1.455475000	4.556310000	0.020991000
1	0.540702000	3.093045000	-0.164260000
8	3.688968000	-0.000720000	-1.453015000
1	4.660652000	-0.000658000	-1.367283000
6	3.859922000	0.000032000	0.839005000
7	4.432386000	0.000371000	1.836415000

Carbenoid e			
29	0.981571000	0.017613000	0.168254000
6	2.940735000	0.008797000	0.288216000
6	-1.631957000	-3.491204000	-0.003605000
6	-1.835566000	0.733520000	-0.022873000
6	-3.007783000	1.472443000	-0.107760000
6	-2.936907000	2.856032000	-0.110738000
6	-1.699728000	3.470728000	-0.029560000
6	-0.576122000	2.666509000	0.052701000
7	-0.640396000	1.336462000	0.056729000
1	-3.967644000	0.985476000	-0.172014000
1	-3.841221000	3.444614000	-0.176819000
1	-1.599322000	4.545558000	-0.030030000
1	0.413368000	3.098902000	0.116419000
6	-1.821293000	-0.757321000	-0.017181000
6	-2.979140000	-1.519474000	-0.093345000
6	-2.881092000	-2.901442000	-0.086128000
6	-0.524012000	-2.664624000	0.070019000
7	-0.614545000	-1.336146000	0.064250000
1	-3.948572000	-1.051898000	-0.158067000
1	-3.773873000	-3.508096000	-0.145129000
1	-1.510638000	-4.563854000	0.003761000
1	0.473910000	-3.077017000	0.135010000
6	3.780955000	0.060046000	1.490966000
1	3.792888000	1.130943000	1.753297000
1	3.288110000	-0.434626000	2.327153000
1	4.810546000	-0.268620000	1.360559000
17	3.836055000	-0.024613000	-1.136743000

Carbenoid f			
29	0.718391000	0.520488000	0.243314000
6	2.641707000	0.675698000	0.463476000
6	-0.694862000	-3.615227000	0.120168000
6	-2.151397000	0.351267000	-0.062491000
6	-3.488141000	0.707481000	-0.174132000
6	-3.828923000	2.048347000	-0.246190000
6	-2.829166000	3.004358000	-0.205754000
6	-1.519088000	2.573849000	-0.089165000
7	-1.187473000	1.285361000	-0.018362000
1	-4.260476000	-0.044469000	-0.202298000
1	-4.866330000	2.339688000	-0.332997000
1	-3.050535000	4.059424000	-0.261137000
1	-0.702123000	3.281578000	-0.051871000
6	-1.699869000	-1.068267000	0.008925000
6	-2.570031000	-2.144568000	-0.089657000
6	-2.058220000	-3.431629000	-0.029670000
6	0.111071000	-2.492182000	0.210415000
7	-0.382224000	-1.256883000	0.161872000
1	-3.630224000	-1.994488000	-0.217740000
1	-2.722456000	-4.281196000	-0.104960000
1	-0.257800000	-4.601347000	0.163676000
1	1.185392000	-2.574098000	0.316216000
6	3.445015000	0.061372000	-0.603562000
8	3.449854000	-1.140928000	-0.645262000
6	3.428062000	1.062629000	1.628340000
1	3.789751000	2.056963000	1.290245000
1	2.855910000	1.217462000	2.536591000
1	4.329479000	0.468536000	1.793262000
8	4.076542000	0.890850000	-1.415481000
1	4.555841000	0.380320000	-2.087940000

Carbenoid g			
29	-1.072123000	0.023429000	-0.185635000
6	-3.009342000	0.019556000	-0.302986000
6	1.498341000	-3.495282000	0.005274000
6	1.724126000	0.728085000	0.028741000
6	2.896600000	1.463636000	0.128539000
6	2.828904000	2.847593000	0.136205000
6	1.594440000	3.466244000	0.044915000
6	0.469125000	2.666404000	-0.052432000
7	0.531099000	1.335927000	-0.061581000
1	3.854308000	0.973647000	0.201563000
1	3.733982000	3.433499000	0.214396000
1	1.496854000	4.541294000	0.049623000
1	-0.518290000	3.101878000	-0.123768000
6	1.703794000	-0.761877000	0.020064000
6	2.855772000	-1.530533000	0.107888000
6	2.749657000	-2.912142000	0.099905000
6	0.395241000	-2.663643000	-0.079977000
7	0.494335000	-1.335390000	-0.074219000
1	3.826946000	-1.068121000	0.182980000
1	3.638365000	-3.523743000	0.168377000
1	1.371107000	-4.567216000	-0.001994000
1	-0.604008000	-3.070733000	-0.153908000
6	-3.704676000	-0.017597000	0.921949000
7	-4.204039000	-0.059701000	1.963229000
6	-3.863312000	0.082426000	-1.491680000
1	-4.132187000	1.155881000	-1.536383000
1	-3.343540000	-0.154184000	-2.413867000
1	-4.813739000	-0.444723000	-1.394044000

Carbenoid h			
29	-0.984517000	-0.003337000	0.232509000
6	-2.981809000	-0.005827000	0.341424000
6	1.667205000	3.483895000	-0.020694000
6	1.833279000	-0.743572000	-0.024453000
6	2.999080000	-1.490048000	-0.134657000
6	2.919720000	-2.873079000	-0.134645000
6	1.680776000	-3.479830000	-0.025624000
6	0.564651000	-2.667934000	0.081398000
7	0.637007000	-1.338500000	0.083421000
1	3.960467000	-1.009618000	-0.220956000
1	3.818726000	-3.467248000	-0.220394000
1	1.573574000	-4.554024000	-0.023760000
1	-0.426166000	-3.093797000	0.166998000
6	1.830389000	0.748253000	-0.023438000
6	2.993089000	1.499417000	-0.134511000
6	2.908322000	2.882133000	-0.132519000
6	0.554417000	2.667502000	0.086878000
7	0.631973000	1.338365000	0.086967000
1	3.956179000	1.022859000	-0.223276000
1	3.804857000	3.479918000	-0.218984000
1	1.555822000	4.557660000	-0.017211000
1	-0.437929000	3.089365000	0.174545000
17	-3.834895000	0.004131000	-1.156748000
7	-3.791694000	-0.013020000	1.355227000
1	-4.803415000	-0.012694000	1.258466000
1	-3.413630000	-0.019252000	2.292284000

Carbenoid i			
29	-0.801557000	-0.000325000	0.341535000
6	-2.781214000	-0.001147000	0.570469000
6	1.847948000	3.481454000	-0.020305000
6	2.006552000	-0.745596000	-0.036262000
6	3.165135000	-1.494607000	-0.195504000
6	3.083284000	-2.877413000	-0.187757000
6	1.849136000	-3.481137000	-0.022017000
6	0.740351000	-2.666413000	0.130987000
7	0.814936000	-1.337309000	0.125087000
1	4.122757000	-1.016259000	-0.324915000
1	3.976513000	-3.473752000	-0.310700000
1	1.740210000	-4.555119000	-0.010839000
1	-0.246404000	-3.089860000	0.262967000
6	2.006287000	0.745968000	-0.035914000
6	3.164543000	1.495454000	-0.195299000
6	3.082229000	2.878229000	-0.186854000
6	0.739499000	2.666281000	0.132727000
7	0.814525000	1.337203000	0.126163000
1	4.122260000	1.017492000	-0.325426000
1	3.975203000	3.474926000	-0.309905000
1	1.738662000	4.555393000	-0.008556000
1	-0.247347000	3.089333000	0.265290000
7	-3.545155000	-0.003049000	1.606691000
1	-4.562566000	-0.003123000	1.493819000
1	-3.169911000	-0.004455000	2.544642000
6	-3.678795000	0.000628000	-0.674784000
8	-4.875117000	0.000134000	-0.630172000
8	-2.950065000	0.002735000	-1.783825000
1	-3.542637000	0.003781000	-2.551403000

Carbenoid j			
29	1.082797000	-0.000912000	-0.239333000
6	3.081770000	-0.001957000	-0.342250000
6	-1.564058000	3.482985000	0.030111000
6	-1.723235000	-0.745217000	0.028194000
6	-2.887123000	-1.492965000	0.147304000
6	-2.806441000	-2.875968000	0.148746000
6	-1.567787000	-3.481836000	0.032348000
6	-0.453224000	-2.669314000	-0.083617000
7	-0.526911000	-1.339676000	-0.087575000
1	-3.848347000	-1.013521000	0.240228000
1	-3.704272000	-3.470773000	0.241930000
1	-1.459385000	-4.555897000	0.032242000
1	0.537066000	-3.095097000	-0.174512000
6	-1.722434000	0.746534000	0.027738000
6	-2.885455000	1.495598000	0.146999000
6	-2.803295000	2.878518000	0.147535000
6	-0.450418000	2.669200000	-0.085872000
7	-0.525524000	1.339640000	-0.088957000
1	-3.847131000	1.017236000	0.240820000
1	-3.700441000	3.474338000	0.240836000
1	-1.454505000	4.556929000	0.029266000
1	0.540279000	3.093869000	-0.177525000
7	3.883480000	-0.004970000	-1.361620000
1	4.896325000	-0.005143000	-1.279182000
1	3.510918000	-0.007346000	-2.301215000
6	3.759410000	0.001106000	0.924449000
7	4.206398000	0.003828000	1.983530000

Methane			
6	0.000000000	0.000000000	0.000000000
1	0.627552000	0.627552000	0.627552000
1	-0.627552000	-0.627552000	0.627552000
1	0.627552000	-0.627552000	-0.627552000
1	-0.627552000	0.627552000	-0.627552000

Ethane			
6	0.000000000	0.000000000	0.761770000
1	-1.013397000	-0.066277000	1.156428000
1	0.449301000	0.910766000	1.156428000
1	0.564096000	-0.844489000	1.156428000
6	0.000000000	0.000000000	-0.761799000
1	-0.454604000	-0.908196000	-1.156371000
1	-0.559218000	0.847797000	-1.156371000
1	1.013823000	0.060399000	-1.156371000

Propane			
6	-0.000014000	0.590992000	-0.000006000
1	0.000078000	1.245376000	0.873883000
1	0.000051000	1.245560000	-0.873762000
6	-1.263599000	-0.260367000	-0.000004000
1	-1.295631000	-0.904622000	0.880095000
1	-2.164313000	0.352782000	0.000203000
1	-1.295831000	-0.904310000	-0.880316000
6	1.263623000	-0.260414000	-0.000007000
1	2.164206000	0.352914000	0.000024000
1	1.295666000	-0.904490000	0.880205000
1	1.295715000	-0.904472000	-0.880233000

Isobutane			
6	0.000003000	0.000027000	0.380939000
1	0.000005000	0.000039000	1.474650000
6	0.123036000	-1.443353000	-0.096202000
1	0.126002000	-1.480399000	-1.188292000
1	1.048542000	-1.900340000	0.255873000
1	-0.711507000	-2.050546000	0.256244000
6	1.188447000	0.828210000	-0.096202000
1	2.131593000	0.409307000	0.256499000
1	1.219279000	0.849009000	-1.188297000
1	1.121379000	1.858329000	0.255565000
6	-1.311497000	0.615110000	-0.096212000
1	-1.345096000	0.631029000	-1.188294000
1	-2.170015000	0.042136000	0.255955000
1	-1.420114000	1.641472000	0.256156000

Reaction with methane

R

a			
29	0.000733000	1.609048000	-0.404882000
6	0.002047000	3.482580000	-0.668419000
1	0.002194000	4.241563000	0.119056000
1	0.002914000	4.012409000	-1.624652000
6	3.478857000	-0.994890000	-0.122112000
6	-0.745658000	-1.165853000	-0.108348000
6	-1.501065000	-2.321420000	0.030933000
6	-2.883827000	-2.230225000	0.024421000
6	-3.480690000	-0.990224000	-0.120565000
6	-2.661643000	0.117672000	-0.254705000
7	-1.332741000	0.032727000	-0.248749000
1	-1.027175000	-3.283493000	0.143331000
1	-3.485685000	-3.121717000	0.131903000

1	-4.553970000	-0.875179000	-0.130670000
1	-3.078985000	1.108636000	-0.370493000
6	0.743602000	-1.166840000	-0.108618000
6	1.497503000	-2.323251000	0.031609000
6	2.880391000	-2.233921000	0.024445000
6	2.661251000	0.114002000	-0.256932000
7	1.332263000	0.030832000	-0.250341000
1	1.022387000	-3.284553000	0.145459000
1	3.481086000	-3.126103000	0.132703000
1	4.552286000	-0.881299000	-0.132838000
1	3.079888000	1.104304000	-0.373727000
6	0.004251000	1.527047000	2.634648000
1	0.897374000	1.991491000	2.218012000
1	0.005591000	1.663485000	3.712767000
1	0.003603000	0.462958000	2.406062000
1	-0.889540000	1.992125000	2.220170000
b			
29	0.910805000	0.000636000	-0.320701000
6	2.880779000	0.003602000	-0.619855000
6	-1.747893000	3.482249000	-0.111579000
6	-1.900011000	-0.746628000	-0.109750000
6	-3.064866000	-1.495642000	-0.006251000
6	-2.983374000	-2.878605000	-0.009570000
6	-1.743263000	-3.483276000	-0.115978000
6	-0.627870000	-2.669499000	-0.216138000
7	-0.702317000	-1.340333000	-0.212914000
1	-4.027523000	-1.017174000	0.076353000
1	-3.881889000	-3.474276000	0.070534000
1	-1.634540000	-4.557290000	-0.122214000
1	0.363603000	-3.094030000	-0.301302000
6	-1.900941000	0.745396000	-0.108629000
6	-3.066986000	1.492733000	-0.006154000
6	-2.987384000	2.875787000	-0.007982000
6	-0.631215000	2.670088000	-0.210374000
7	-0.703850000	1.340825000	-0.208825000
1	-4.029134000	1.012870000	0.074326000
1	-3.886837000	3.470178000	0.071096000
1	-1.640655000	4.556419000	-0.116668000
1	0.359908000	3.096009000	-0.292667000
1	1.270061000	0.892111000	2.215327000
6	0.817688000	-0.002823000	2.642718000
1	0.990097000	-0.004447000	3.715848000
1	-0.252575000	-0.001339000	2.446767000

1	1.268264000	-0.897491000	2.212894000
17	4.020964000	-0.003592000	0.671476000
8	3.436565000	0.010794000	-1.772100000
1	4.414165000	0.011009000	-1.758835000
c			
29	-0.753850000	0.000079000	-0.364973000
6	-2.696653000	0.000580000	-0.725166000
6	1.901493000	-3.481904000	-0.146790000
6	2.058366000	0.745736000	-0.132985000
6	3.222292000	1.494694000	-0.020024000
6	3.140816000	2.877672000	-0.025904000
6	1.901734000	3.481990000	-0.145318000
6	0.787264000	2.667921000	-0.254061000
7	0.861586000	1.338914000	-0.246901000
1	4.184151000	1.016081000	0.071182000
1	4.038513000	3.473561000	0.061357000
1	1.793105000	4.555986000	-0.155465000
1	-0.203311000	3.092145000	-0.349902000
6	2.058296000	-0.745672000	-0.133259000
6	3.222117000	-1.494755000	-0.020036000
6	3.140565000	-2.877720000	-0.026531000
6	0.787124000	-2.667725000	-0.255648000
7	0.861515000	-1.338713000	-0.247895000
1	4.183945000	-1.016226000	0.071929000
1	4.038177000	-3.473707000	0.060929000
1	1.792789000	-4.555887000	-0.157461000
1	-0.203436000	-3.091847000	-0.352081000
1	-0.679273000	0.891281000	2.253250000
6	-0.241179000	-0.001018000	2.698644000
1	-0.679082000	-0.893128000	2.252698000
1	0.834641000	-0.000820000	2.536081000
1	-0.445845000	-0.001363000	3.766296000
6	-3.849925000	-0.000253000	0.310133000
8	-4.990969000	0.000665000	-0.059443000
8	-3.416615000	-0.002047000	1.551567000
1	-4.169190000	-0.002518000	2.164602000
8	-3.225189000	0.001819000	-1.880363000
1	-4.216925000	0.001965000	-1.808044000
d			
29	-1.005751000	0.000182000	-0.361082000
6	-2.978217000	0.000247000	-0.706448000
6	1.644798000	-3.496586000	-0.084982000
6	1.801642000	0.747827000	-0.092030000

6	2.969642000	1.498952000	0.047371000
6	2.888622000	2.888567000	0.050209000
6	1.645299000	3.496400000	-0.084797000
6	0.527916000	2.679035000	-0.220630000
7	0.600718000	1.343430000	-0.226289000
1	3.937877000	1.015458000	0.155065000
1	3.792479000	3.488145000	0.159070000
1	1.534610000	4.579567000	-0.084844000
1	-0.469577000	3.108751000	-0.327153000
6	1.801525000	-0.748038000	-0.092072000
6	2.969425000	-1.499334000	0.047256000
6	2.888211000	-2.888938000	0.050023000
6	0.527527000	-2.679055000	-0.220746000
7	0.600512000	-1.343462000	-0.226334000
1	3.937733000	-1.015984000	0.154940000
1	3.791985000	-3.488651000	0.158828000
1	1.533956000	-4.579737000	-0.085082000
1	-0.470029000	-3.108626000	-0.327271000
1	-1.412790000	-0.906182000	2.052993000
6	-1.052838000	0.000028000	2.563665000
1	-1.448988000	-0.000015000	3.586195000
1	0.044883000	0.000133000	2.589891000
1	-1.412965000	0.906177000	2.053007000
6	-3.855514000	-0.000050000	0.446815000
7	-4.407964000	-0.000317000	1.466617000
8	-3.667110000	0.000404000	-1.795383000
1	-3.076614000	0.000574000	-2.570964000
e			
29	0.938223000	0.016384000	-0.317261000
6	2.893840000	0.037878000	-0.537378000
6	-1.669505000	3.505318000	0.016696000
6	-1.883758000	-0.689362000	-0.167646000
6	-3.050796000	-1.427501000	-0.036609000
6	-2.987464000	-2.808311000	-0.103577000
6	-1.762789000	-3.419981000	-0.299899000
6	-0.643468000	-2.615346000	-0.416043000
7	-0.701123000	-1.289864000	-0.350620000
1	-4.000014000	-0.941543000	0.124479000
1	-3.887776000	-3.397663000	-0.000781000
1	-1.668204000	-4.493726000	-0.358548000
1	0.338113000	-3.045534000	-0.562552000
6	-1.863412000	0.798918000	-0.102692000
6	-3.018618000	1.555050000	-0.010109000

6	-2.918035000	2.922167000	0.050643000
6	-0.569467000	2.682401000	-0.082408000
7	-0.660023000	1.374606000	-0.141227000
1	-3.990070000	1.087538000	0.008397000
1	-3.809514000	3.528763000	0.122823000
1	-1.543304000	4.576232000	0.063473000
1	0.430144000	3.091735000	-0.113452000
1	0.511924000	0.407584000	2.270105000
6	0.578587000	-0.599772000	2.681507000
1	0.631492000	-0.533325000	3.764936000
1	-0.297240000	-1.175555000	2.391845000
1	1.474542000	-1.096611000	2.311396000
17	3.896877000	-0.033842000	0.812161000
6	3.641845000	0.170813000	-1.794720000
1	3.094808000	-0.282440000	-2.620260000
1	3.623628000	1.255519000	-1.992254000
1	4.682347000	-0.147620000	-1.759409000
f			
29	0.674702000	0.445362000	-0.410763000
6	2.588580000	0.581567000	-0.745876000
6	-2.776948000	3.073197000	0.062357000
6	-1.806201000	-1.041429000	-0.121786000
6	-2.713098000	-2.082128000	0.023818000
6	-2.252574000	-3.388686000	-0.024828000
6	-0.902161000	-3.627169000	-0.211368000
6	-0.058194000	-2.537372000	-0.347443000
7	-0.501803000	-1.283219000	-0.308335000
1	-3.762433000	-1.888920000	0.180475000
1	-2.945787000	-4.210514000	0.087709000
1	-0.503919000	-4.629873000	-0.248279000
1	1.008477000	-2.663411000	-0.484358000
6	-2.202773000	0.395192000	-0.063137000
6	-3.522791000	0.802897000	0.072441000
6	-3.811117000	2.156495000	0.135350000
6	-1.486800000	2.591723000	-0.077500000
7	-1.205842000	1.291306000	-0.140047000
1	-4.322247000	0.081187000	0.125361000
1	-4.834640000	2.488093000	0.240456000
1	-2.957082000	4.136475000	0.110034000
1	-0.644536000	3.267445000	-0.140355000
1	1.360321000	-0.712417000	2.101419000
6	0.892045000	0.148338000	2.578383000
1	1.329611000	1.075711000	2.207568000

1	-0.177213000	0.141802000	2.374957000
1	1.051604000	0.090558000	3.652107000
6	3.317843000	0.916807000	-1.962798000
1	3.687319000	1.928906000	-1.693730000
1	4.214566000	0.318849000	-2.139043000
1	2.702827000	1.021005000	-2.850012000
6	3.442401000	0.033364000	0.316609000
8	3.498979000	-1.165532000	0.400008000
8	4.048014000	0.915530000	1.093211000
1	4.555079000	0.446246000	1.775411000
g			
29	1.004086000	-0.023106000	-0.347742000
6	2.931350000	-0.018336000	-0.622384000
6	-1.572050000	3.495965000	-0.123979000
6	-1.795249000	-0.728127000	-0.106254000
6	-2.965829000	-1.463198000	0.020992000
6	-2.898623000	-2.847104000	0.030887000
6	-1.666573000	-3.466429000	-0.085246000
6	-0.543488000	-2.666917000	-0.209504000
7	-0.605112000	-1.336842000	-0.221067000
1	-3.921623000	-0.972786000	0.113658000
1	-3.802022000	-3.432391000	0.130192000
1	-1.569282000	-4.541519000	-0.079466000
1	0.442357000	-3.102790000	-0.300402000
6	-1.775220000	0.762207000	-0.114684000
6	-2.925736000	1.530092000	0.000419000
6	-2.820769000	2.911771000	-0.004923000
6	-0.470696000	2.664903000	-0.236076000
7	-0.568732000	1.337016000	-0.232874000
1	-3.894924000	1.067007000	0.094670000
1	-3.708210000	3.522462000	0.084716000
1	-1.445553000	4.568013000	-0.129994000
1	0.526855000	3.072737000	-0.329336000
1	1.365530000	0.891746000	2.203084000
6	0.910925000	-0.001328000	2.631606000
1	1.085531000	0.002265000	3.704217000
1	-0.159959000	-0.002638000	2.437334000
1	1.366337000	-0.897038000	2.209128000
6	3.734722000	0.014278000	0.534636000
7	4.321345000	0.051698000	1.529706000
6	3.676265000	-0.076722000	-1.883127000
1	4.628698000	0.455769000	-1.870555000
1	3.946440000	-1.148379000	-1.952696000

1	3.074660000	0.158181000	-2.754554000
h			
29	0.928374000	0.001557000	-0.339398000
6	2.917015000	0.000729000	-0.618786000
6	-1.747082000	3.480339000	-0.115118000
6	-1.898487000	-0.748188000	-0.121436000
6	-3.062656000	-1.497856000	-0.011174000
6	-2.979460000	-2.880633000	-0.005962000
6	-1.738489000	-3.484160000	-0.109904000
6	-0.624629000	-2.669043000	-0.218235000
7	-0.700635000	-1.340196000	-0.225090000
1	-4.025714000	-1.020042000	0.071156000
1	-3.876958000	-3.477017000	0.080117000
1	-1.628170000	-4.558056000	-0.107125000
1	0.367924000	-3.092277000	-0.299621000
6	-1.900295000	0.743954000	-0.122237000
6	-3.065759000	1.490877000	-0.007118000
6	-2.986007000	2.873859000	-0.004219000
6	-0.631675000	2.667850000	-0.227171000
7	-0.704382000	1.338798000	-0.231772000
1	-4.027112000	1.010774000	0.081571000
1	-3.884542000	3.468120000	0.085662000
1	-1.639462000	4.554512000	-0.114500000
1	0.359441000	3.093445000	-0.313537000
1	1.201738000	0.903477000	2.196458000
6	0.752560000	0.011523000	2.633110000
1	0.936332000	0.014636000	3.704414000
1	-0.319658000	0.011868000	2.447935000
1	1.200216000	-0.883782000	2.201834000
17	3.946990000	0.000241000	0.763875000
7	3.601225000	0.000000000	-1.722237000
1	4.617204000	-0.000763000	-1.747456000
1	3.113398000	0.000135000	-2.607061000
i			
29	0.760161000	0.234602000	-0.485347000
6	2.696582000	0.674337000	-0.717798000
6	-2.648973000	2.858257000	0.359803000
6	-1.772433000	-1.230049000	-0.267312000
6	-2.720006000	-2.244576000	-0.303870000
6	-2.303776000	-3.550108000	-0.507502000
6	-0.954534000	-3.812598000	-0.669880000
6	-0.072837000	-2.745609000	-0.631340000
7	-0.469230000	-1.489662000	-0.439050000

1	-3.770094000	-2.026810000	-0.189118000
1	-3.028686000	-4.351352000	-0.540939000
1	-0.587610000	-4.815580000	-0.827527000
1	0.991155000	-2.897363000	-0.756614000
6	-2.133781000	0.198302000	-0.040482000
6	-3.405871000	0.592162000	0.354378000
6	-3.664132000	1.938035000	0.556596000
6	-1.405919000	2.385549000	-0.025209000
7	-1.153054000	1.093299000	-0.218511000
1	-4.184781000	-0.135349000	0.519651000
1	-4.647634000	2.260674000	0.868416000
1	-2.807072000	3.916173000	0.505717000
1	-0.577472000	3.064280000	-0.180115000
1	0.758773000	1.307714000	2.129213000
6	0.156012000	0.571334000	2.659789000
1	0.545875000	0.449419000	3.667097000
1	-0.875257000	0.915563000	2.707432000
1	0.200330000	-0.385196000	2.139891000
6	3.705500000	0.314391000	0.381092000
8	4.856911000	0.641800000	0.356889000
8	3.129664000	-0.400075000	1.339920000
1	3.791580000	-0.609261000	2.017093000
7	3.346262000	1.228012000	-1.681551000
1	4.358067000	1.364452000	-1.608358000
1	2.881881000	1.552613000	-2.518025000j
29	1.008716000	0.000199000	-0.371261000
6	2.996182000	0.000163000	-0.672008000
6	-1.649498000	3.482877000	-0.091552000
6	-1.804931000	-0.746027000	-0.101770000
6	-2.967215000	-1.493819000	0.036068000
6	-2.886517000	-2.876755000	0.040015000
6	-1.649506000	-3.482795000	-0.092325000
6	-0.536966000	-2.670079000	-0.226801000
7	-0.610688000	-1.340831000	-0.233141000
1	-3.927147000	-1.014395000	0.141740000
1	-3.782850000	-3.471360000	0.147711000
1	-1.541018000	-4.556869000	-0.090916000
1	0.452371000	-3.095829000	-0.330126000
6	-1.804946000	0.746101000	-0.101660000
6	-2.967303000	1.493881000	0.035499000
6	-2.886596000	2.876826000	0.039813000
6	-0.536886000	2.670169000	-0.225566000
7	-0.610618000	1.340930000	-0.232240000

1	-3.927334000	1.014456000	0.140267000
1	-3.783014000	3.471394000	0.147002000
1	-1.540965000	4.556946000	-0.089814000
1	0.452508000	3.095947000	-0.328238000
1	1.422804000	0.893698000	2.143893000
6	0.981029000	-0.001252000	2.582308000
1	1.188251000	-0.001822000	3.649167000
1	-0.094823000	-0.001035000	2.418669000
1	1.422578000	-0.895877000	2.143007000
6	3.821396000	-0.000004000	0.503741000
7	4.389746000	-0.000113000	1.503090000
7	3.671638000	0.000359000	-1.779636000
1	4.686978000	0.000369000	-1.819237000
1	3.188874000	0.000463000	-2.667643000
TS			
a			
29	-0.982568000	-1.008507000	-0.027607000
6	-2.502587000	-2.179527000	-0.049593000
1	-2.961580000	-2.629211000	-0.932521000
1	-2.998717000	-2.603195000	0.825968000
6	3.229583000	-2.147520000	0.020556000
6	0.781123000	1.299934000	-0.000369000
6	1.243626000	2.608597000	0.010266000
6	0.328652000	3.649035000	0.003254000
6	-1.024979000	3.361156000	-0.013721000
6	-1.408676000	2.031322000	-0.023249000
7	-0.532245000	1.029387000	-0.017259000
1	2.300061000	2.824112000	0.024280000
1	0.673547000	4.673504000	0.011310000
1	-1.770486000	4.141821000	-0.019323000
1	-2.454491000	1.753852000	-0.035964000
6	1.691301000	0.120141000	0.007082000
6	3.074087000	0.237191000	0.021027000
6	3.850081000	-0.910687000	0.027879000
6	1.845864000	-2.184009000	0.006300000
7	1.097155000	-1.082610000	-0.000255000
1	3.548929000	1.205292000	0.026345000
1	4.928409000	-0.835487000	0.038779000
1	3.796296000	-3.066265000	0.025570000
1	1.314388000	-3.125810000	-0.000198000
1	-3.784995000	-0.731882000	-0.181701000
6	-4.853210000	-0.809504000	0.093815000
1	-5.343490000	0.043695000	-0.369277000

1	-4.947919000	-0.758962000	1.174275000
1	-5.289026000	-1.728968000	-0.283827000
	b		
29	-0.724250000	-0.289787000	-0.178751000
6	-2.796773000	0.023940000	-0.194966000
6	2.420285000	-3.307187000	-0.006063000
6	1.940989000	0.900833000	0.006240000
6	2.988044000	1.811941000	0.055125000
6	2.702331000	3.168029000	0.054416000
6	1.383481000	3.583780000	0.002936000
6	0.395468000	2.614449000	-0.048289000
7	0.668409000	1.312652000	-0.046023000
1	4.014179000	1.482561000	0.089329000
1	3.506108000	3.889918000	0.092256000
1	1.119646000	4.630608000	0.000198000
1	-0.652483000	2.880671000	-0.095667000
6	2.157526000	-0.576659000	0.004941000
6	3.421342000	-1.142018000	0.114282000
6	3.553061000	-2.520577000	0.107798000
6	1.194948000	-2.672731000	-0.107706000
7	1.063285000	-1.346707000	-0.103415000
1	4.298302000	-0.522134000	0.208931000
1	4.531556000	-2.972066000	0.193051000
1	2.475924000	-4.385208000	-0.014244000
1	0.281267000	-3.244991000	-0.194377000
1	-2.798851000	-0.872272000	-0.969848000
6	-4.113270000	-1.018488000	-1.235623000
1	-4.151179000	-2.072612000	-0.973558000
1	-3.999276000	-0.821357000	-2.298778000
1	-4.932757000	-0.466846000	-0.794724000
17	-3.406999000	-0.340457000	1.412399000
8	-3.054686000	1.267576000	-0.644454000
1	-3.939172000	1.571238000	-0.386444000
	c		
29	0.575338000	0.091966000	-0.223801000
6	2.613840000	-0.360292000	-0.285540000
6	-1.959516000	-3.506204000	0.097018000
6	-2.255745000	0.714206000	0.016793000
6	-3.442994000	1.424977000	0.136658000
6	-3.412311000	2.809208000	0.105077000
6	-2.197636000	3.455869000	-0.043038000
6	-1.056700000	2.680712000	-0.153382000
7	-1.082217000	1.348958000	-0.126177000
1	-4.384331000	0.913620000	0.258543000
1	-4.329536000	3.373755000	0.197949000
1	-2.127860000	4.532748000	-0.071012000
1	-0.082716000	3.138106000	-0.265689000
6	-2.209422000	-0.778053000	0.045985000
6	-3.351812000	-1.562606000	0.134523000
6	-3.221557000	-2.942178000	0.160710000
6	-0.868985000	-2.656964000	0.005852000
7	-0.992812000	-1.332698000	-0.019097000
1	-4.332990000	-1.117544000	0.178918000
1	-4.100985000	-3.567114000	0.229177000
1	-1.816022000	-4.576044000	0.115450000
1	0.141164000	-3.041755000	-0.050500000
1	2.611662000	0.287047000	-1.322775000
6	3.849956000	0.411703000	-1.731351000
1	3.957815000	1.489303000	-1.669831000
1	3.592053000	0.039489000	-2.720270000
1	4.684459000	-0.132111000	-1.309528000
6	3.404601000	0.268507000	0.819484000
8	4.079646000	-0.396888000	1.554730000
8	3.244194000	1.590375000	0.907178000
1	3.783851000	1.915407000	1.643306000
8	2.833988000	-1.676873000	-0.417691000
1	3.630891000	-1.921958000	0.086936000
	d		
29	-0.763887000	0.492632000	0.032987000
6	-2.858904000	0.617074000	0.207135000
6	2.758004000	3.064748000	-0.021588000
6	1.719497000	-1.042304000	-0.027248000
6	2.638207000	-2.081903000	-0.080424000
6	2.179919000	-3.389940000	-0.070016000
6	0.819194000	-3.630425000	-0.008080000
6	-0.036091000	-2.541532000	0.037753000
7	0.402680000	-1.284580000	0.027480000
1	3.697509000	-1.888264000	-0.133391000
1	2.883302000	-4.209835000	-0.110862000
1	0.420190000	-4.633476000	0.003118000
1	-1.109413000	-2.677041000	0.082770000
6	2.132026000	0.393069000	-0.028291000
6	3.465155000	0.782869000	-0.017890000
6	3.779859000	2.131453000	-0.015470000
6	1.454374000	2.601767000	-0.028769000
7	1.147330000	1.304971000	-0.033258000

1	4.256065000	0.050463000	-0.005661000
1	4.813960000	2.446301000	-0.006110000
1	2.957782000	4.125564000	-0.017809000
1	0.621357000	3.291697000	-0.028414000
1	-2.858253000	1.246176000	-0.838229000
6	-4.085452000	1.336189000	-1.270553000
1	-4.037072000	0.697219000	-2.146241000
1	-3.954777000	2.397326000	-1.475600000
1	-4.952050000	1.147580000	-0.653142000
8	-3.249267000	1.313142000	1.292925000
1	-4.113061000	1.032882000	1.628620000
6	-3.316612000	-0.740891000	0.069730000
7	-3.603401000	-1.851690000	-0.005195000
e			
29	0.703369000	0.435742000	-0.086661000
6	2.762468000	0.369418000	-0.232276000
6	-2.695365000	3.167322000	0.088136000
6	-1.870509000	-0.984511000	-0.006267000
6	-2.837396000	-1.980177000	0.046050000
6	-2.441254000	-3.307619000	0.014607000
6	-1.093516000	-3.610750000	-0.065744000
6	-0.190539000	-2.561331000	-0.110755000
7	-0.568518000	-1.286339000	-0.083173000
1	-3.885688000	-1.737447000	0.115164000
1	-3.181396000	-4.094473000	0.055267000
1	-0.742970000	-4.631526000	-0.090828000
1	0.875204000	-2.742917000	-0.166710000
6	-2.209745000	0.468758000	0.025644000
6	-3.520956000	0.926470000	0.054869000
6	-3.764994000	2.289125000	0.087157000
6	-1.418062000	2.636622000	0.053909000
7	-1.177888000	1.326361000	0.023660000
1	-4.348553000	0.235533000	0.049255000
1	-4.780993000	2.657654000	0.110050000
1	-2.839011000	4.236935000	0.112944000
1	-0.550714000	3.282953000	0.050133000
1	2.808802000	1.409653000	0.452639000
6	3.978923000	1.837605000	0.627757000
1	3.863186000	1.956725000	1.703105000
1	3.920865000	2.766326000	0.067677000
1	4.840555000	1.237505000	0.378339000
17	3.350108000	-0.994203000	0.711458000
6	3.369899000	0.434442000	-1.602276000
1	4.446787000	0.258205000	-1.617420000
1	2.893849000	-0.350415000	-2.192504000
1	3.141656000	1.387840000	-2.077020000
f			
29	-0.556462000	-0.447600000	-0.198538000
6	-2.566242000	-0.339564000	-0.434338000
6	1.199826000	3.597417000	-0.097699000
6	2.338582000	-0.473625000	0.056599000
6	3.647145000	-0.925992000	0.166747000
6	3.893976000	-2.287684000	0.216730000
6	2.829904000	-3.170534000	0.155562000
6	1.554568000	-2.645728000	0.044804000
7	1.312011000	-1.336451000	-0.003423000
1	4.470237000	-0.231175000	0.213198000
1	4.908164000	-2.651892000	0.302642000
1	2.976351000	-4.239425000	0.191683000
1	0.690991000	-3.294989000	-0.007199000
6	1.994827000	0.977920000	0.001874000
6	2.953445000	1.980072000	0.065785000
6	2.547368000	3.304472000	0.013900000
6	0.302493000	2.542889000	-0.155945000
7	0.693229000	1.271396000	-0.109126000
1	4.002090000	1.745639000	0.155922000
1	3.281016000	4.096992000	0.062561000
1	0.843392000	4.615665000	-0.138199000
1	-0.764433000	2.711767000	-0.234746000
1	-2.868202000	-1.655370000	0.124263000
6	-3.855670000	-2.253786000	0.129641000
1	-3.776912000	-2.779265000	1.077871000
1	-3.784044000	-2.910241000	-0.731646000
1	-4.733768000	-1.624076000	0.101216000
6	-3.152174000	-0.238117000	-1.800263000
1	-2.767049000	-0.995941000	-2.476040000
1	-4.241639000	-0.205639000	-1.833076000
1	-2.791718000	0.734681000	-2.156134000
6	-3.118261000	0.613327000	0.556238000
8	-2.995466000	1.791313000	0.333175000
8	-3.618259000	0.107081000	1.680794000
1	-3.852675000	0.845495000	2.263568000
g			
29	0.775126000	0.459124000	-0.111607000
6	2.818396000	0.453008000	-0.294952000
6	-0.934428000	-3.619276000	-0.008210000

6	-2.127696000	0.439246000	0.026586000
6	-3.447429000	0.872280000	0.042179000
6	-3.717852000	2.230189000	0.061465000
6	-2.665613000	3.129375000	0.063732000
6	-1.378181000	2.623724000	0.041917000
7	-1.112912000	1.317748000	0.023349000
1	-4.261382000	0.165361000	0.033848000
1	-4.740991000	2.578975000	0.073019000
1	-2.830308000	4.196102000	0.079183000
1	-0.523304000	3.286457000	0.038874000
6	-1.760924000	-1.007640000	0.012196000
6	-2.707374000	-2.019659000	0.098503000
6	-2.286017000	-3.340144000	0.085842000
6	-0.050593000	-2.555069000	-0.086995000
7	-0.454077000	-1.286777000	-0.079171000
1	-3.758720000	-1.795324000	0.182149000
1	-3.010425000	-4.139726000	0.153259000
1	-0.563868000	-4.633206000	-0.017363000
1	1.017656000	-2.720536000	-0.150592000
1	3.004816000	1.245627000	0.837424000
6	4.021283000	1.484559000	1.386199000
1	3.822789000	1.088508000	2.378279000
1	4.028324000	2.570539000	1.338354000
1	4.898891000	1.042865000	0.936096000
6	3.279015000	-0.843747000	0.096763000
7	3.543024000	-1.930841000	0.371003000
6	3.546033000	1.070667000	-1.446299000
1	3.160471000	0.538518000	-2.324045000
1	3.286373000	2.118588000	-1.569256000
1	4.627048000	0.936677000	-1.432443000
		h	
29	0.706091000	-0.459721000	0.114416000
6	2.795263000	-0.447388000	0.269890000
6	-1.011158000	3.615600000	0.077191000
6	-2.200797000	-0.443118000	-0.029481000
6	-3.520287000	-0.875820000	-0.061597000
6	-3.789996000	-2.233650000	-0.097696000
6	-2.737338000	-3.132093000	-0.099052000
6	-1.450130000	-2.626167000	-0.060218000
7	-1.185629000	-1.320578000	-0.026386000
1	-4.334694000	-0.169397000	-0.053177000
1	-4.812795000	-2.582720000	-0.122588000
1	-2.901194000	-4.198716000	-0.126822000
1	-0.595233000	-3.288867000	-0.055174000
6	-1.834643000	1.003690000	0.006160000
6	-2.781128000	2.016702000	-0.076134000
6	-2.361532000	3.336845000	-0.038473000
6	-0.128632000	2.550393000	0.148559000
7	-0.529512000	1.282293000	0.115308000
1	-3.831175000	1.792740000	-0.175436000
1	-3.085742000	4.136869000	-0.102530000
1	-0.642738000	4.629905000	0.108002000
1	0.938581000	2.711739000	0.229872000
1	2.681700000	-1.432792000	-0.404230000
6	3.934657000	-1.777425000	-0.645665000
1	3.808439000	-1.823008000	-1.724852000
1	3.863148000	-2.733597000	-0.134084000
1	4.825211000	-1.229721000	-0.371853000
17	3.297511000	0.956714000	-0.713529000
7	3.310226000	-0.548217000	1.536911000
1	4.232254000	-0.166093000	1.705607000
1	2.668642000	-0.398469000	2.297457000
		i	
29	0.540754000	-0.625469000	0.227964000
6	2.633615000	-0.547417000	0.474438000
6	-0.761025000	3.610518000	0.119723000
6	-2.326489000	-0.318731000	-0.036659000
6	-3.674305000	-0.621394000	-0.182758000
6	-4.074892000	-1.946300000	-0.222454000
6	-3.122081000	-2.944318000	-0.116362000
6	-1.798468000	-2.567479000	0.023857000
7	-1.408253000	-1.293571000	0.062402000
1	-4.410416000	0.161504000	-0.268597000
1	-5.121268000	-2.192874000	-0.336047000
1	-3.389282000	-3.989880000	-0.142078000
1	-1.017470000	-3.310796000	0.108841000
6	-1.825874000	1.087916000	0.015565000
6	-2.675018000	2.185644000	-0.031749000
6	-2.132748000	3.460219000	0.020991000
6	0.021248000	2.467495000	0.165246000
7	-0.499620000	1.243537000	0.114261000
1	-3.743756000	2.063414000	-0.105140000
1	-2.780541000	4.324981000	-0.014507000
1	-0.299686000	4.585676000	0.161804000
1	1.100541000	2.526453000	0.238269000
1	2.613278000	-1.716202000	0.172162000

6	3.892405000	-2.049513000	0.004578000
1	3.738753000	-2.559470000	-0.941910000
1	3.922121000	-2.694183000	0.877326000
1	4.736443000	-1.375339000	-0.026473000
6	2.970165000	0.350612000	-0.652819000
8	3.239068000	1.512967000	-0.501644000
8	2.882292000	-0.241851000	-1.855613000
1	3.085976000	0.421973000	-2.529715000
7	3.047366000	-0.178886000	1.721720000
1	3.780746000	0.511925000	1.789432000
1	2.396183000	-0.188573000	2.485448000
j			
29	-0.788608000	-0.463299000	-0.043063000
6	-2.912868000	-0.597644000	-0.224815000
6	0.872789000	3.622169000	-0.018859000
6	2.110498000	-0.424083000	0.026027000
6	3.436900000	-0.837078000	0.056700000
6	3.729183000	-2.190128000	0.077915000
6	2.691850000	-3.106581000	0.068676000
6	1.397232000	-2.621148000	0.037748000
7	1.110895000	-1.319600000	0.016763000
1	4.240120000	-0.118269000	0.065203000
1	4.757737000	-2.521923000	0.102011000
1	2.873571000	-4.170524000	0.085168000
1	0.553564000	-3.298188000	0.029893000
6	1.723948000	1.018444000	0.006105000
6	2.663384000	2.040194000	0.007220000
6	2.229732000	3.356841000	-0.006220000
6	-0.005368000	2.549963000	-0.020380000
7	0.410462000	1.285063000	-0.009454000
1	3.720193000	1.827448000	0.018249000
1	2.949560000	4.163380000	-0.005660000
1	0.492081000	4.632331000	-0.027289000
1	-1.076879000	2.704012000	-0.025666000
1	-2.814879000	-1.271356000	0.757703000
6	-4.089970000	-1.339106000	1.213140000
1	-3.926233000	-0.865953000	2.177214000
1	-4.051482000	-2.426108000	1.235080000
1	-4.980053000	-0.974876000	0.720011000
6	-3.211652000	0.778564000	0.053294000
7	-3.407339000	1.891797000	0.264595000
7	-3.336491000	-1.124629000	-1.412564000
1	-4.279081000	-0.975489000	-1.739676000
1	-2.845235000	-1.899684000	-1.818087000

P

a			
29	1.184224000	-0.732920000	-0.047074000
6	3.291181000	-1.649689000	-0.050087000
1	3.647361000	-2.669734000	-0.175464000
1	2.748321000	-1.435210000	-0.991787000
6	-2.755634000	-2.643034000	0.016382000
6	-0.957123000	1.194875000	0.003445000
6	-1.670652000	2.386328000	0.012551000
6	-0.985443000	3.590133000	0.003855000
6	0.397965000	3.581425000	-0.014784000
6	1.040582000	2.356107000	-0.024311000
7	0.384902000	1.196448000	-0.014779000
1	-2.748455000	2.388418000	0.024325000
1	-1.530801000	4.523440000	0.010749000
1	0.971892000	4.495670000	-0.022796000
1	2.120555000	2.298222000	-0.040729000
6	-1.633502000	-0.138414000	0.010621000
6	-3.015703000	-0.267835000	0.049931000
6	-3.580499000	-1.532813000	0.052648000
6	-1.387913000	-2.436629000	-0.020021000
7	-0.841857000	-1.221410000	-0.022787000
1	-3.654774000	0.599631000	0.079739000
1	-4.655170000	-1.645534000	0.083235000
1	-3.153600000	-3.646506000	0.016784000
1	-0.701764000	-3.272374000	-0.048104000
1	2.639926000	-1.699115000	0.844465000
6	4.426310000	-0.655953000	0.160295000
1	5.005014000	-0.918196000	1.043115000
1	4.052217000	0.357815000	0.296581000
1	5.098052000	-0.654095000	-0.695157000
b			
29	-0.589222000	0.551554000	0.078972000
6	-3.755607000	-0.111213000	0.238036000
6	3.118467000	2.901845000	-0.102706000
6	1.781478000	-1.116216000	0.008578000
6	2.625173000	-2.219514000	0.020296000
6	2.078171000	-3.491590000	0.060228000
6	0.702418000	-3.636274000	0.086614000
6	-0.075200000	-2.490753000	0.073673000
7	0.449281000	-1.267142000	0.036877000
1	3.696438000	-2.100509000	0.002021000

1	2.724202000	-4.358301000	0.070950000
1	0.235496000	-4.609152000	0.117717000
1	-1.155569000	-2.543175000	0.089771000
6	2.299581000	0.285051000	-0.033962000
6	3.656586000	0.573784000	-0.101690000
6	4.068074000	1.896076000	-0.135952000
6	1.784974000	2.536431000	-0.037925000
7	1.386640000	1.266436000	-0.005092000
1	4.392024000	-0.213907000	-0.130114000
1	5.121242000	2.134132000	-0.188564000
1	3.395447000	3.944948000	-0.126804000
1	1.004506000	3.285096000	-0.010668000
1	-3.729921000	-0.388479000	1.285628000
6	-5.116106000	0.263702000	-0.273512000
1	-5.558792000	1.046569000	0.336384000
1	-5.751221000	-0.621294000	-0.232116000
1	-5.053393000	0.614180000	-1.304107000
17	-2.705876000	1.469470000	0.236781000
8	-3.098035000	-1.066066000	-0.479987000
1	-3.308351000	-0.980661000	-1.418230000
c			
29	-0.323405000	-0.164976000	0.068321000
6	-4.656177000	0.313745000	0.052511000
6	2.033777000	3.552946000	-0.080243000
6	2.538882000	-0.648980000	0.043275000
6	3.770911000	-1.290620000	0.039499000
6	3.817361000	-2.673593000	0.096822000
6	2.633884000	-3.388226000	0.157752000
6	1.445195000	-2.679987000	0.154571000
7	1.396332000	-1.349824000	0.096821000
1	4.689165000	-0.727854000	-0.007876000
1	4.770222000	-3.184093000	0.094884000
1	2.623745000	-4.466582000	0.206792000
1	0.494234000	-3.193349000	0.202215000
6	2.412484000	0.838814000	-0.006369000
6	3.517376000	1.679980000	-0.033478000
6	3.323343000	3.051273000	-0.071620000
6	0.983549000	2.651189000	-0.051031000
7	1.167288000	1.333264000	-0.016777000
1	4.519980000	1.283830000	-0.021956000
1	4.174070000	3.717946000	-0.091981000
1	1.838271000	4.614310000	-0.107019000
1	-0.043280000	2.992380000	-0.052555000
1	-5.176414000	0.896904000	-0.707494000
6	-5.451682000	-0.951323000	0.368448000
1	-5.611667000	-1.552287000	-0.524818000
1	-6.415443000	-0.659261000	0.778081000
1	-4.923775000	-1.553422000	1.110189000
6	-3.318047000	-0.089381000	-0.528618000
8	-2.292105000	-0.100771000	0.127997000
8	-3.378956000	-0.464827000	-1.789680000
1	-2.513106000	-0.766307000	-2.101208000
8	-4.479212000	1.147811000	1.155516000
1	-4.095529000	0.645269000	1.881349000
d			
29	-0.469361000	-0.107072000	-0.081558000
6	-5.053827000	0.021423000	-0.324768000
6	1.996654000	3.531004000	0.018847000
6	2.385425000	-0.683553000	0.029276000
6	3.595924000	-1.362092000	0.086737000
6	3.597661000	-2.747166000	0.082144000
6	2.392828000	-3.425018000	0.020546000
6	1.227904000	-2.679428000	-0.032446000
7	1.222357000	-1.347828000	-0.027824000
1	4.530687000	-0.827266000	0.136456000
1	4.532681000	-3.287847000	0.126802000
1	2.348526000	-4.503606000	0.014073000
1	0.261159000	-3.162391000	-0.080604000
6	2.301569000	0.807196000	0.027834000
6	3.428462000	1.618478000	0.059338000
6	3.271840000	2.994887000	0.054923000
6	0.923097000	2.657406000	-0.012523000
7	1.070310000	1.334425000	-0.008204000
1	4.419222000	1.194054000	0.084725000
1	4.139857000	3.638783000	0.078493000
1	1.829921000	4.597565000	0.013752000
1	-0.093469000	3.026495000	-0.042795000
1	-5.289249000	0.056018000	-1.389442000
6	-5.693052000	-1.201025000	0.316324000
1	-5.358369000	-2.117404000	-0.166007000
1	-6.772098000	-1.117107000	0.209225000
1	-5.441136000	-1.253081000	1.375759000
6	-3.568512000	-0.070407000	-0.242970000
7	-2.429532000	-0.124224000	-0.166796000
8	-5.476229000	1.237436000	0.218845000
1	-5.564217000	1.154544000	1.174189000

e			
29	-0.640281000	-0.567707000	0.003351000
6	-3.892498000	-0.284836000	-0.004958000
6	0.560855000	3.658315000	0.000981000
6	2.241267000	-0.232456000	-0.000706000
6	3.606198000	-0.489171000	-0.007176000
6	4.049068000	-1.802049000	-0.007168000
6	3.122623000	-2.829513000	-0.000825000
6	1.779269000	-2.495132000	0.004742000
7	1.351578000	-1.234580000	0.004694000
1	4.324901000	0.314195000	-0.012839000
1	5.108754000	-2.015566000	-0.012250000
1	3.424515000	-3.865956000	-0.000466000
1	1.015653000	-3.261504000	0.009430000
6	1.688341000	1.156047000	0.000183000
6	2.508956000	2.277284000	0.003287000
6	1.939458000	3.539372000	0.003646000
6	-0.190985000	2.497097000	-0.001427000
7	0.351723000	1.280473000	-0.001706000
1	3.582270000	2.178347000	0.006060000
1	2.569097000	4.418064000	0.006122000
1	0.073530000	4.621563000	0.001050000
1	-1.272388000	2.541934000	-0.003264000
1	-4.838829000	-0.818882000	-0.006516000
6	-3.708494000	0.515575000	1.261468000
1	-3.831148000	-0.100107000	2.149136000
1	-4.452543000	1.312653000	1.285305000
1	-2.716263000	0.972252000	1.285658000
17	-2.671082000	-1.670715000	0.005066000
6	-3.698986000	0.505906000	-1.276036000
1	-3.814551000	-0.116610000	-2.159884000
1	-4.443172000	1.302412000	-1.311754000
1	-2.706824000	0.962973000	-1.296083000

f			
29	-0.501902000	0.496079000	0.000389000
6	-3.784732000	-0.441015000	-0.000948000
6	3.085246000	3.054680000	-0.000180000
6	1.983759000	-1.032898000	0.000171000
6	2.889683000	-2.086467000	0.000981000
6	2.419702000	-3.388941000	0.000998000
6	1.054398000	-3.614223000	0.000221000
6	0.214530000	-2.514627000	-0.000411000
7	0.661505000	-1.259976000	-0.000400000
1	3.952047000	-1.903572000	0.001736000
1	3.115398000	-4.216335000	0.001633000
1	0.643473000	-4.612488000	0.000172000
1	-0.860798000	-2.641133000	-0.000961000
6	2.422940000	0.394966000	-0.000035000
6	3.762362000	0.762863000	-0.001435000
6	4.094327000	2.107992000	-0.001488000
6	1.774182000	2.609280000	0.001028000
7	1.453133000	1.318045000	0.001088000
1	4.545126000	0.021564000	-0.002643000
1	5.132529000	2.409137000	-0.002583000
1	3.300513000	4.112568000	-0.000149000
1	0.947802000	3.307774000	0.001994000
1	-4.871799000	-0.489770000	-0.001309000
6	-3.408324000	1.014528000	0.000201000
8	-2.260522000	1.437800000	0.000520000
8	-4.430130000	1.841073000	0.000961000
1	-4.108565000	2.756298000	0.001757000
6	-3.243298000	-1.105208000	-1.268925000
1	-2.153347000	-1.040793000	-1.310082000
1	-3.521883000	-2.157764000	-1.272576000
1	-3.644486000	-0.644630000	-2.170273000
6	-3.244049000	-1.106835000	1.266508000
1	-3.522888000	-2.159318000	1.268799000
1	-2.154096000	-1.042683000	1.308188000
1	-3.645473000	-0.647178000	2.168224000

g			
29	-0.471783000	-0.102349000	-0.023900000
6	-5.025952000	-0.437101000	-0.074440000
6	1.966113000	3.547888000	0.048987000
6	2.395953000	-0.662439000	-0.001083000
6	3.614028000	-1.329784000	0.006916000
6	3.627489000	-2.714775000	-0.011334000
6	2.427212000	-3.402849000	-0.037082000
6	1.254527000	-2.667242000	-0.042950000
7	1.237903000	-1.336245000	-0.025353000
1	4.545382000	-0.787092000	0.027747000
1	4.568157000	-3.247406000	-0.005261000
1	2.392322000	-4.481716000	-0.052070000
1	0.290568000	-3.157959000	-0.062480000
6	2.297013000	0.827172000	0.016873000
6	3.416379000	1.649352000	0.035204000
6	3.246934000	3.023973000	0.051460000

6	0.900771000	2.664154000	0.029649000
7	1.059977000	1.342507000	0.013926000
1	4.411291000	1.234002000	0.036359000
1	4.109108000	3.675951000	0.065619000
1	1.789014000	4.612710000	0.061370000
1	-0.119563000	3.023683000	0.026475000
1	-5.251275000	-1.478530000	-0.313585000
6	-5.579377000	-0.100187000	1.314987000
1	-5.169633000	-0.755179000	2.080515000
1	-6.660620000	-0.219968000	1.296944000
1	-5.351548000	0.932308000	1.575165000
6	-3.566079000	-0.339786000	-0.061240000
7	-2.426184000	-0.244444000	-0.046219000
6	-5.596362000	0.473549000	-1.167718000
1	-5.197675000	0.222861000	-2.148107000
1	-6.677529000	0.353906000	-1.190914000
1	-5.370059000	1.516282000	-0.950526000
h			
29	-0.561456000	0.176971000	-0.240513000
6	-4.495011000	-0.407912000	0.003388000
6	2.530718000	3.306366000	0.178616000
6	2.142456000	-0.903835000	0.015367000
6	3.207976000	-1.794241000	0.068844000
6	2.962139000	-3.154661000	-0.005368000
6	1.657737000	-3.599343000	-0.133324000
6	0.650098000	-2.652368000	-0.182531000
7	0.881217000	-1.342856000	-0.109512000
1	4.221664000	-1.438956000	0.161357000
1	3.782971000	-3.857010000	0.033782000
1	1.420095000	-4.650480000	-0.196879000
1	-0.384726000	-2.950193000	-0.287086000
6	2.335123000	0.575612000	0.084459000
6	3.581100000	1.160915000	0.270305000
6	3.676281000	2.542612000	0.316902000
6	1.324899000	2.647658000	0.001504000
7	1.232451000	1.322161000	-0.043236000
1	4.469854000	0.560635000	0.381888000
1	4.638416000	3.014045000	0.460717000
1	2.562393000	4.385109000	0.207703000
1	0.397266000	3.194416000	-0.108973000
1	-4.238554000	-0.990496000	-0.868989000
6	-4.106342000	-0.907901000	1.349905000
1	-3.102100000	-1.316486000	1.334070000
1	-4.810060000	-1.695750000	1.629715000
1	-4.156464000	-0.110161000	2.088485000
17	-2.670475000	0.942834000	-0.438791000
7	-5.560130000	0.351016000	-0.113481000
1	-5.892888000	0.885519000	0.675332000
1	-5.905385000	0.619801000	-1.021593000
i			
29	-0.559066000	0.098734000	0.352208000
6	-3.552223000	0.515003000	0.406326000
6	2.278994000	3.432013000	-0.152107000
6	2.220462000	-0.795702000	0.002776000
6	3.331036000	-1.595412000	-0.239505000
6	3.195787000	-2.972832000	-0.192584000
6	1.956195000	-3.520331000	0.088421000
6	0.896955000	-2.656009000	0.307818000
7	1.023440000	-1.332337000	0.269952000
1	4.287781000	-1.159140000	-0.478845000
1	4.049689000	-3.608523000	-0.380638000
1	1.805277000	-4.588467000	0.131729000
1	-0.093949000	-3.036306000	0.519070000
6	2.287951000	0.694381000	-0.041394000
6	3.495701000	1.378292000	-0.115852000
6	3.489650000	2.761578000	-0.172608000
6	1.120149000	2.680412000	-0.062416000
7	1.119954000	1.350320000	-0.005949000
1	4.433718000	0.846259000	-0.117075000
1	4.421405000	3.306703000	-0.229775000
1	2.226639000	4.509351000	-0.198260000
1	0.150993000	3.160925000	-0.035982000
1	-3.615270000	1.591227000	0.226791000
6	-4.893344000	0.010749000	0.927735000
1	-5.693666000	0.238896000	0.229121000
1	-5.117542000	0.483618000	1.882155000
1	-4.864338000	-1.070094000	1.073568000
6	-3.195892000	-0.087815000	-0.943644000
8	-2.102242000	-0.505932000	-1.236640000
8	-4.219308000	-0.077785000	-1.783509000
1	-3.931052000	-0.437562000	-2.635766000
7	-2.434515000	0.284122000	1.336832000
1	-2.577076000	-0.601882000	1.814146000
1	-2.447961000	0.990732000	2.063266000
j			
29	-0.469464000	-0.118520000	-0.093080000

6	-5.042959000	-0.044971000	-0.311268000
6	1.965761000	3.540089000	0.035220000
6	2.392878000	-0.670440000	0.028267000
6	3.608922000	-1.338976000	0.087247000
6	3.622145000	-2.723942000	0.076648000
6	2.423194000	-3.411456000	0.007634000
6	1.252271000	-2.675291000	-0.046624000
7	1.235743000	-1.343930000	-0.036288000
1	4.538959000	-0.796469000	0.142550000
1	4.561437000	-3.257080000	0.122404000
1	2.387922000	-4.490343000	-0.003601000
1	0.289608000	-3.165781000	-0.100541000
6	2.295892000	0.819476000	0.033223000
6	3.415108000	1.640814000	0.076241000
6	3.245645000	3.015695000	0.077302000
6	0.900479000	2.656753000	-0.007374000
7	1.060206000	1.335333000	-0.008439000
1	4.409543000	1.225374000	0.106679000
1	4.107434000	3.667523000	0.109868000
1	1.789206000	4.605082000	0.034191000
1	-0.119485000	3.016008000	-0.042393000
1	-5.282322000	-0.121492000	-1.373589000
6	-5.637322000	-1.241254000	0.435230000
1	-5.271221000	-2.185994000	0.036235000
1	-6.720003000	-1.212195000	0.330512000
1	-5.387244000	-1.187661000	1.494282000
6	-3.566385000	-0.104462000	-0.248451000
7	-2.426714000	-0.154015000	-0.180132000
7	-5.450437000	1.270364000	0.148076000
1	-5.522499000	1.297563000	1.157920000
1	-6.352232000	1.516068000	-0.237477000
Reaction with ethane<br>R			
a			
29	-0.094693000	1.647237000	-0.541313000
6	0.040037000	3.524807000	-0.720822000
1	0.281015000	4.230847000	0.078711000
1	-0.105250000	4.109585000	-1.632946000
6	3.123226000	-1.259729000	-0.896121000
6	-1.036819000	-1.059926000	-0.172843000
6	-1.866007000	-2.150799000	0.044870000
6	-3.215504000	-1.938455000	0.277305000
6	-3.706414000	-0.644642000	0.286144000

6	-2.819146000	0.393012000	0.057391000
7	-1.521611000	0.191723000	-0.165664000
1	-1.475113000	-3.155694000	0.030073000
1	-3.874313000	-2.778328000	0.448080000
1	-4.750495000	-0.435578000	0.463442000
1	-3.153368000	1.421402000	0.052750000
6	0.424283000	-1.191716000	-0.426862000
6	1.090020000	-2.409060000	-0.387433000
6	2.454641000	-2.441034000	-0.625301000
6	2.391732000	-0.084627000	-0.917781000
7	1.079909000	-0.051862000	-0.691556000
1	0.561805000	-3.322863000	-0.166443000
1	2.987730000	-3.380930000	-0.595208000
1	4.185914000	-1.240143000	-1.084763000
1	2.867960000	0.864816000	-1.121846000
6	1.883180000	-0.050952000	2.740298000
1	1.362987000	-0.614690000	3.513125000
1	2.003452000	-0.705879000	1.875846000
1	2.877760000	0.183940000	3.115794000
6	1.120232000	1.218266000	2.381869000
1	1.639153000	1.783290000	1.603689000
1	0.111829000	0.986372000	2.028909000
1	1.016184000	1.874063000	3.245116000
b			
29	0.939048000	0.224367000	-0.485447000
6	2.919573000	0.287702000	-0.674796000
6	-1.694887000	3.612559000	0.430892000
6	-1.872713000	-0.527830000	-0.412408000
6	-3.028679000	-1.295499000	-0.349751000
6	-2.955398000	-2.651487000	-0.623162000
6	-1.732477000	-3.210142000	-0.952766000
6	-0.624793000	-2.380522000	-0.989318000
7	-0.691427000	-1.077203000	-0.726178000
1	-3.973555000	-0.853192000	-0.076696000
1	-3.845250000	-3.263281000	-0.573994000
1	-1.630794000	-4.262121000	-1.172713000
1	0.353939000	-2.770682000	-1.235801000
6	-1.864464000	0.933832000	-0.118423000
6	-3.030403000	1.666485000	0.059286000
6	-2.942346000	3.020705000	0.337669000
6	-0.578945000	2.817964000	0.231405000
7	-0.659427000	1.516212000	-0.036672000
1	-3.997882000	1.198172000	-0.028185000

1	-3.841133000	3.605021000	0.476717000
1	-1.581143000	4.664044000	0.647234000
1	0.417552000	3.235063000	0.288455000
1	0.657200000	-0.222035000	2.008401000
6	0.873925000	-1.271559000	2.229843000
1	1.622016000	-1.283781000	3.021578000
1	1.328664000	-1.728739000	1.347325000
6	-0.388204000	-2.016532000	2.645913000
1	-0.837481000	-1.562076000	3.527418000
1	-1.136889000	-2.012343000	1.852313000
1	-0.166632000	-3.055307000	2.885541000
17	3.965454000	-0.008467000	0.662383000
8	3.554105000	0.551641000	-1.753301000
1	4.528277000	0.548313000	-1.670073000
c			
29	0.804154000	0.135987000	-0.528977000
6	2.757878000	0.069155000	-0.809746000
6	-1.612937000	3.695198000	0.318644000
6	-2.055058000	-0.445596000	-0.422491000
6	-3.262147000	-1.132152000	-0.386539000
6	-3.272719000	-2.494771000	-0.635463000
6	-2.080773000	-3.141144000	-0.913787000
6	-0.918799000	-2.388596000	-0.929071000
7	-0.905326000	-1.079085000	-0.690771000
1	-4.184576000	-0.622184000	-0.158956000
1	-4.203744000	-3.043375000	-0.608302000
1	-2.043933000	-4.201535000	-1.112792000
1	0.038378000	-2.847630000	-1.138658000
6	-1.954528000	1.018516000	-0.158910000
6	-3.069370000	1.821323000	0.042617000
6	-2.894474000	3.174092000	0.284503000
6	-0.552031000	2.832335000	0.103501000
7	-0.716009000	1.531600000	-0.128674000
1	-4.064646000	1.408072000	0.006112000
1	-3.752967000	3.811852000	0.442098000
1	-1.431761000	4.743230000	0.503487000
1	0.468077000	3.191896000	0.117030000
1	0.200261000	-0.195953000	2.019951000
6	0.404932000	-1.223456000	2.332619000
1	1.085444000	-1.170794000	3.181616000
1	0.936050000	-1.728581000	1.522148000
6	-0.878222000	-1.958618000	2.697349000
1	-1.404524000	-1.449945000	3.503642000

1	-1.558906000	-2.024975000	1.847574000
1	-0.666707000	-2.973773000	3.029672000
6	3.841877000	0.027597000	0.296477000
8	5.002774000	-0.037116000	0.002282000
8	3.329662000	0.077219000	1.506844000
1	4.040177000	0.048796000	2.167455000
8	3.356265000	0.059636000	-1.929891000
1	4.340885000	0.022473000	-1.797728000
d			
29	1.016873000	0.227443000	-0.536562000
6	2.985887000	0.323398000	-0.761417000
6	-1.608338000	3.594235000	0.454627000
6	-1.775979000	-0.546655000	-0.390808000
6	-2.922093000	-1.324656000	-0.292025000
6	-2.842282000	-2.682976000	-0.552196000
6	-1.622337000	-3.234215000	-0.904465000
6	-0.524324000	-2.394827000	-0.979172000
7	-0.597620000	-1.088479000	-0.730789000
1	-3.864102000	-0.888780000	0.000480000
1	-3.724606000	-3.302542000	-0.473695000
1	-1.515169000	-4.288097000	-1.112323000
1	0.451916000	-2.779893000	-1.242829000
6	-1.772580000	0.915711000	-0.101558000
6	-2.938085000	1.637662000	0.116162000
6	-2.852863000	2.991631000	0.397321000
6	-0.492345000	2.811035000	0.215504000
7	-0.570517000	1.509732000	-0.057775000
1	-3.903764000	1.160954000	0.060141000
1	-3.751765000	3.567183000	0.568432000
1	-1.496758000	4.645134000	0.674879000
1	0.501730000	3.236635000	0.246204000
1	0.882138000	-0.183575000	1.936230000
6	1.096329000	-1.234332000	2.158701000
1	1.883841000	-1.243091000	2.910500000
1	1.504776000	-1.711190000	1.263375000
6	-0.153604000	-1.957478000	2.643311000
1	-0.555346000	-1.484912000	3.538075000
1	-0.939773000	-1.957169000	1.886482000
1	0.068303000	-2.994811000	2.888547000
6	3.780677000	0.110180000	0.423652000
7	4.263072000	-0.078019000	1.450117000
8	3.742021000	0.558674000	-1.775267000
1	3.220155000	0.704011000	-2.578778000

e			
29	0.967217000	0.197123000	-0.461979000
6	2.929226000	0.247465000	-0.611006000
6	-1.610908000	3.634119000	0.429364000
6	-1.864775000	-0.495520000	-0.441928000
6	-3.037501000	-1.239234000	-0.413527000
6	-2.984074000	-2.596286000	-0.686238000
6	-1.763638000	-3.179853000	-0.980112000
6	-0.638866000	-2.372736000	-0.985525000
7	-0.686677000	-1.068092000	-0.724905000
1	-3.980663000	-0.777743000	-0.167614000
1	-3.887203000	-3.189890000	-0.663375000
1	-1.676852000	-4.233959000	-1.196223000
1	0.338646000	-2.782804000	-1.202887000
6	-1.831844000	0.963904000	-0.141515000
6	-2.983838000	1.720016000	0.028199000
6	-2.869688000	3.070116000	0.317131000
6	-0.509950000	2.816985000	0.237433000
7	-0.615617000	1.519422000	-0.041780000
1	-3.960122000	1.272873000	-0.071519000
1	-3.757169000	3.672675000	0.450826000
1	-1.477170000	4.681161000	0.655643000
1	0.494782000	3.211207000	0.311001000
1	0.565874000	-0.237910000	2.040490000
6	0.762834000	-1.287362000	2.279051000
1	1.483489000	-1.300426000	3.095711000
1	1.242520000	-1.755489000	1.415919000
6	-0.520225000	-2.015660000	2.658769000
1	-0.994392000	-1.549287000	3.520858000
1	-1.242049000	-2.011153000	1.840589000
1	-0.317989000	-3.054595000	2.914436000
17	3.855990000	-0.057855000	0.760741000
6	3.743988000	0.506752000	-1.804696000
1	4.773979000	0.805430000	-1.617981000
1	3.756276000	-0.465442000	-2.324685000
1	3.228535000	1.186791000	-2.481988000

f			
29	0.732294000	0.523398000	-0.500160000
6	2.649692000	0.746174000	-0.739952000
6	-2.505822000	3.304076000	0.439414000
6	-1.861263000	-0.781030000	-0.444486000
6	-2.839148000	-1.766015000	-0.409916000
6	-2.482787000	-3.079035000	-0.673549000
6	-1.162369000	-3.379543000	-0.961029000
6	-0.244273000	-2.342824000	-0.976686000
7	-0.587134000	-1.080472000	-0.730355000
1	-3.861654000	-1.525376000	-0.165394000
1	-3.230925000	-3.858836000	-0.645905000
1	-0.842835000	-4.390356000	-1.164776000
1	0.802480000	-2.523083000	-1.186027000
6	-2.144790000	0.650554000	-0.142033000
6	-3.431746000	1.137637000	0.039406000
6	-3.612160000	2.479446000	0.333535000
6	-1.254565000	2.747807000	0.236385000
7	-1.077335000	1.458709000	-0.047721000
1	-4.287484000	0.488106000	-0.054942000
1	-4.608549000	2.873662000	0.476354000
1	-2.601589000	4.354414000	0.669493000
1	-0.359310000	3.350962000	0.304572000
1	0.398111000	0.052689000	2.016085000
6	0.818693000	-0.913103000	2.310393000
1	1.490493000	-0.727251000	3.147991000
1	1.419909000	-1.304019000	1.486103000
6	-0.280364000	-1.896668000	2.693644000
1	-0.886306000	-1.509677000	3.511354000
1	-0.946058000	-2.099745000	1.853406000
1	0.142605000	-2.847051000	3.014901000
6	3.401536000	1.268304000	-1.878272000
1	4.280680000	1.840851000	-1.561561000
1	3.820480000	0.363429000	-2.354208000
1	2.806713000	1.806917000	-2.606453000
6	3.503331000	0.078338000	0.255335000
8	3.732961000	-1.090907000	0.096841000
8	3.907381000	0.840834000	1.258471000
1	4.431462000	0.304033000	1.874535000

g			
29	1.029146000	0.226556000	-0.507226000
6	2.965768000	0.310731000	-0.675073000
6	-1.573618000	3.604195000	0.448522000
6	-1.763261000	-0.529004000	-0.423684000
6	-2.917704000	-1.298776000	-0.374736000
6	-2.837865000	-2.655183000	-0.645071000
6	-1.609913000	-3.212464000	-0.957741000
6	-0.503596000	-2.380968000	-0.985709000
7	-0.577677000	-1.076809000	-0.727812000
1	-3.867058000	-0.857947000	-0.115261000

1	-3.726942000	-3.268785000	-0.605709000
1	-1.503019000	-4.265153000	-1.171583000
1	0.478830000	-2.769610000	-1.218763000
6	-1.755067000	0.930005000	-0.122590000
6	-2.916201000	1.659295000	0.091717000
6	-2.822146000	3.011204000	0.380407000
6	-0.461421000	2.813881000	0.214808000
7	-0.548752000	1.514728000	-0.064874000
1	-3.884951000	1.189692000	0.028976000
1	-3.717671000	3.592718000	0.549010000
1	-1.455733000	4.653350000	0.673664000
1	0.535811000	3.230832000	0.254182000
1	0.757420000	-0.199676000	1.995629000
6	0.967172000	-1.246859000	2.236260000
1	1.713565000	-1.246112000	3.028896000
1	1.426167000	-1.722327000	1.365575000
6	-0.300786000	-1.978236000	2.657829000
1	-0.754648000	-1.504818000	3.526961000
1	-1.045249000	-1.989305000	1.859787000
1	-0.084141000	-3.012414000	2.920678000
6	3.686054000	0.054362000	0.508952000
7	4.199676000	-0.158232000	1.522190000
6	3.793465000	0.578704000	-1.853394000
1	4.734165000	1.089087000	-1.641000000
1	4.083743000	-0.440481000	-2.175572000
1	3.246454000	1.027685000	-2.675439000
	h		
29	0.961090000	0.165861000	-0.510197000
6	2.959748000	0.187983000	-0.683343000
6	-1.555620000	3.657089000	0.410556000
6	-1.892423000	-0.476260000	-0.415679000
6	-3.075206000	-1.200828000	-0.335818000
6	-3.053286000	-2.561416000	-0.594339000
6	-1.853471000	-3.167665000	-0.925011000
6	-0.717227000	-2.378554000	-0.979466000
7	-0.734474000	-1.070414000	-0.733090000
1	-4.000983000	-0.721797000	-0.059173000
1	-3.964123000	-3.140148000	-0.530997000
1	-1.790907000	-4.225511000	-1.131095000
1	0.245658000	-2.807085000	-1.225247000
6	-1.828578000	0.986236000	-0.132221000
6	-2.965760000	1.762882000	0.047349000
6	-2.825191000	3.113367000	0.322208000
6	-0.471612000	2.819754000	0.208318000
7	-0.602400000	1.521996000	-0.057753000
1	-3.950708000	1.331361000	-0.034854000
1	-3.700496000	3.731758000	0.463749000
1	-1.401111000	4.703516000	0.626325000
1	0.540778000	3.197571000	0.263250000
1	0.622493000	-0.244286000	1.993664000
6	0.811142000	-1.296973000	2.224533000
1	1.557223000	-1.321487000	3.017755000
1	1.257738000	-1.771107000	1.346936000
6	-0.470075000	-2.006857000	2.643224000
1	-0.909234000	-1.535838000	3.521252000
1	-1.217699000	-1.988619000	1.848725000
1	-0.275871000	-3.049603000	2.889697000
17	3.881913000	0.005718000	0.762372000
7	3.723606000	0.335459000	-1.722317000
1	3.302755000	0.451659000	-2.633527000
1	4.738589000	0.340610000	-1.670831000
	i		
29	0.821088000	-0.021339000	-0.607054000
6	2.799574000	-0.200766000	-0.837653000
6	-1.284999000	3.760821000	0.171830000
6	-2.087718000	-0.353216000	-0.392104000
6	-3.343372000	-0.936750000	-0.275878000
6	-3.474138000	-2.303277000	-0.460080000
6	-2.350234000	-3.056565000	-0.752770000
6	-1.133666000	-2.402296000	-0.847542000
7	-1.004470000	-1.088882000	-0.674763000
1	-4.210766000	-0.343314000	-0.034351000
1	-4.443914000	-2.772322000	-0.370304000
1	-2.406290000	-4.124516000	-0.900426000
1	-0.225225000	-2.948188000	-1.066539000
6	-1.860828000	1.107994000	-0.197006000
6	-2.900196000	2.008358000	-0.002016000
6	-2.606321000	3.349380000	0.183861000
6	-0.305902000	2.803872000	-0.035385000
7	-0.584715000	1.515505000	-0.217639000
1	-3.927248000	1.679679000	-0.000729000
1	-3.404132000	4.062907000	0.335453000
1	-1.012570000	4.795496000	0.315645000
1	0.741874000	3.073441000	-0.054835000
1	0.209370000	-0.168221000	1.984809000
6	0.385271000	-1.190695000	2.327456000

1	1.092239000	-1.136606000	3.154636000
1	0.873420000	-1.741954000	1.519448000
6	-0.912435000	-1.865970000	2.751911000
1	-1.391425000	-1.317217000	3.561492000
1	-1.623204000	-1.923967000	1.926289000
1	-0.731040000	-2.880748000	3.102797000
6	3.731917000	0.041179000	0.356362000
8	4.907742000	-0.183672000	0.336027000
8	3.061016000	0.530136000	1.392302000
1	3.677819000	0.671513000	2.127131000
7	3.531942000	-0.487575000	-1.856887000
1	3.128408000	-0.690518000	-2.760565000
1	4.550039000	-0.534098000	-1.762898000
j			
29	1.022352000	0.232181000	-0.537563000
6	3.018724000	0.319242000	-0.736479000
6	-1.618731000	3.599588000	0.464164000
6	-1.788087000	-0.537058000	-0.398102000
6	-2.934641000	-1.314451000	-0.293885000
6	-2.857941000	-2.671587000	-0.560175000
6	-1.640720000	-3.221833000	-0.923360000
6	-0.542779000	-2.382345000	-1.001533000
7	-0.612669000	-1.077297000	-0.747498000
1	-3.873700000	-0.878967000	0.008730000
1	-3.739962000	-3.291036000	-0.477433000
1	-1.535724000	-4.274963000	-1.136188000
1	0.431691000	-2.766571000	-1.273258000
6	-1.783027000	0.924562000	-0.103714000
6	-2.949708000	1.648320000	0.104068000
6	-2.864322000	3.000814000	0.391359000
6	-0.502521000	2.813902000	0.233015000
7	-0.580402000	1.514335000	-0.046139000
1	-3.915797000	1.174106000	0.034373000
1	-3.763499000	3.578158000	0.554859000
1	-1.506802000	4.649290000	0.690006000
1	0.492564000	3.236424000	0.275114000
1	0.859818000	-0.220394000	1.945876000
6	1.070764000	-1.274215000	2.154431000
1	1.853744000	-1.296877000	2.910692000
1	1.483128000	-1.735529000	1.252997000
6	-0.183277000	-2.003073000	2.619528000
1	-0.589271000	-1.545650000	3.520338000
1	-0.965128000	-1.986933000	1.858387000

1	0.034147000	-3.045412000	2.847484000
6	3.764673000	0.104317000	0.472506000
7	4.264976000	-0.079047000	1.491222000
7	3.762547000	0.548546000	-1.773930000
1	3.337135000	0.713293000	-2.675891000
1	4.777858000	0.574950000	-1.743269000
TS			
a			
29	0.898396000	-0.864266000	-0.243233000
6	2.500472000	-2.035706000	-0.375241000
1	2.904407000	-2.560474000	0.492909000
1	2.721900000	-2.646237000	-1.259282000
6	-3.193919000	-2.429676000	0.154924000
6	-1.127636000	1.255801000	-0.009891000
6	-1.731787000	2.505513000	0.050987000
6	-0.945632000	3.643186000	-0.019079000
6	0.425944000	3.509581000	-0.149095000
6	0.951544000	2.230708000	-0.203967000
7	0.201186000	1.133071000	-0.136306000
1	-2.801321000	2.600135000	0.149288000
1	-1.402412000	4.621881000	0.026470000
1	1.076474000	4.369058000	-0.208104000
1	2.017845000	2.076176000	-0.306801000
6	-1.904855000	-0.015246000	0.056310000
6	-3.284773000	-0.042948000	0.209875000
6	-3.934562000	-1.265557000	0.259310000
6	-1.821604000	-2.318640000	0.006039000
7	-1.194028000	-1.146082000	-0.041514000
1	-3.854497000	0.868818000	0.292218000
1	-5.008302000	-1.304111000	0.378356000
1	-3.660048000	-3.402876000	0.187751000
1	-1.198668000	-3.199060000	-0.078623000
1	3.644759000	-1.401824000	-0.676855000
6	4.259438000	-0.514528000	-0.222803000
1	4.001023000	0.277722000	-0.919893000
1	5.254461000	-0.908172000	-0.427505000
6	4.020114000	-0.184841000	1.222371000
1	4.234615000	-1.032079000	1.869994000
1	4.692953000	0.625915000	1.503784000
1	2.996778000	0.144024000	1.396351000
b			
29	-0.369939000	0.565780000	-0.011948000
6	-2.246898000	1.261265000	0.002749000

6	0.818513000	-3.658261000	0.306255000
6	2.525564000	0.210899000	-0.071575000
6	3.894358000	0.470207000	-0.153180000
6	4.330912000	1.786612000	-0.271342000
6	3.392754000	2.812297000	-0.306104000
6	2.046481000	2.472098000	-0.222099000
7	1.622121000	1.208911000	-0.108126000
1	4.621009000	-0.338608000	-0.128046000
1	5.396962000	2.004873000	-0.336154000
1	3.689147000	3.856011000	-0.397273000
1	1.272123000	3.240307000	-0.246997000
6	1.974209000	-1.173327000	0.059839000
6	2.791204000	-2.301425000	0.145579000
6	2.204728000	-3.557567000	0.269585000
6	0.072996000	-2.487022000	0.216208000
7	0.631814000	-1.277882000	0.095392000
1	3.874830000	-2.212307000	0.119907000
1	2.829807000	-4.448015000	0.337992000
1	0.317154000	-4.619832000	0.403361000
1	-1.017755000	-2.513157000	0.240306000
1	-2.687067000	-1.161873000	-0.748954000
6	-3.619262000	-1.751979000	-0.708042000
1	-3.693668000	-2.204072000	0.292308000
1	-3.510772000	-2.565754000	-1.439156000
6	-4.842385000	-0.897665000	-1.022735000
1	-4.748808000	-0.408047000	-2.003600000
1	-5.753754000	-1.510285000	-1.048966000
1	-5.009541000	-0.126681000	-0.252257000
17	-3.140817000	1.370448000	1.482010000
8	-2.928717000	1.603763000	-1.024106000
1	-3.864388000	1.838128000	-0.828981000
c			
29	-0.336430000	0.105180000	0.110809000
6	-2.387419000	-0.279646000	0.044785000
6	2.149227000	-3.536079000	-0.106697000
6	2.519593000	0.675695000	0.061236000
6	3.724847000	1.365229000	0.029920000
6	3.716820000	2.749262000	0.079569000
6	2.506789000	3.416638000	0.156928000
6	1.347265000	2.661648000	0.181026000
7	1.350999000	1.330320000	0.136405000
1	4.662858000	0.837916000	-0.037777000
1	4.648064000	3.297710000	0.055072000

1	2.454832000	4.494198000	0.195550000
1	0.375884000	3.135032000	0.235431000
6	2.447381000	-0.814810000	0.006071000
6	3.578080000	-1.620679000	-0.022428000
6	3.423393000	-2.996716000	-0.079813000
6	1.071511000	-2.666435000	-0.071115000
7	1.218863000	-1.345643000	-0.015580000
1	4.568408000	-1.194717000	0.002473000
1	4.293225000	-3.638229000	-0.102511000
1	1.986876000	-4.602386000	-0.152784000
1	0.052449000	-3.031201000	-0.086989000
1	-2.446463000	0.258599000	1.113372000
6	-3.719721000	0.531515000	1.504697000
1	-3.873567000	1.530201000	1.113062000
1	-3.287817000	0.559089000	2.507095000
6	-4.852198000	-0.432087000	1.372769000
1	-4.559146000	-1.434695000	1.676654000
1	-5.650170000	-0.105711000	2.042927000
1	-5.258816000	-0.456872000	0.362192000
6	-3.084330000	0.440609000	-1.061833000
8	-3.748090000	-0.152142000	-1.869558000
8	-2.856497000	1.757863000	-1.087079000
1	-3.331166000	2.131484000	-1.844317000
8	-2.658012000	-1.595491000	0.059162000
1	-3.414635000	-1.770086000	-0.529592000
d			
29	0.523575000	-0.381390000	0.102792000
6	2.624111000	-0.330794000	0.326850000
6	-2.751691000	-3.269430000	-0.071190000
6	-2.093197000	0.916054000	-0.037972000
6	-3.104435000	1.866196000	-0.072243000
6	-2.768106000	3.211052000	-0.063321000
6	-1.434712000	3.574750000	-0.021156000
6	-0.481352000	2.569467000	0.013501000
7	-0.803499000	1.277490000	0.005779000
1	-4.142443000	1.576688000	-0.104343000
1	-3.544490000	3.962840000	-0.089573000
1	-1.129008000	4.610074000	-0.013926000
1	0.575455000	2.801946000	0.045711000
6	-2.371802000	-0.551032000	-0.049618000
6	-3.662384000	-1.061670000	-0.116471000
6	-3.853086000	-2.432803000	-0.126737000
6	-1.498173000	-2.688922000	-0.006889000

7	-1.309304000	-1.369417000	0.004137000
1	-4.515462000	-0.404257000	-0.161650000
1	-4.852958000	-2.840494000	-0.178234000
1	-2.853876000	-4.344054000	-0.077345000
1	-0.607685000	-3.301503000	0.037168000
1	2.720826000	-1.008756000	-0.635022000
6	3.980954000	-0.890256000	-1.180855000
1	3.768900000	-0.128609000	-1.924712000
1	3.793838000	-1.898384000	-1.557317000
6	5.262598000	-0.750344000	-0.431089000
1	5.318007000	-1.442599000	0.404998000
1	6.070258000	-0.996867000	-1.124325000
1	5.415129000	0.266383000	-0.075456000
6	2.870525000	1.067878000	0.095964000
7	2.984567000	2.195860000	-0.094848000
8	3.211779000	-0.798625000	1.459841000
1	2.687743000	-1.511499000	1.836195000
	e		
29	0.464730000	-0.329115000	0.107374000
6	2.512707000	-0.072415000	0.286562000
6	-2.675306000	-3.356845000	-0.067420000
6	-2.229671000	0.853145000	-0.019310000
6	-3.281021000	1.756582000	-0.106543000
6	-3.006782000	3.114681000	-0.082117000
6	-1.694207000	3.538940000	0.026153000
6	-0.701427000	2.575860000	0.103247000
7	-0.962407000	1.271919000	0.081664000
1	-4.301061000	1.419184000	-0.198175000
1	-3.813530000	3.831075000	-0.149645000
1	-1.437859000	4.587430000	0.047853000
1	0.342178000	2.852587000	0.181103000
6	-2.435579000	-0.625088000	-0.037027000
6	-3.700025000	-1.199655000	-0.067112000
6	-3.819932000	-2.579032000	-0.083181000
6	-1.451396000	-2.712439000	-0.033008000
7	-1.330551000	-1.385660000	-0.018197000
1	-4.586463000	-0.585995000	-0.072576000
1	-4.798389000	-3.038070000	-0.105832000
1	-2.721574000	-4.435301000	-0.078823000
1	-0.529159000	-3.277438000	-0.015648000
1	2.657629000	-1.032412000	-0.408742000
6	3.871803000	-1.378213000	-0.790283000
1	3.702085000	-1.060608000	-1.816414000

1	3.613227000	-2.426029000	-0.632035000
6	5.200774000	-0.987761000	-0.234598000
1	5.357246000	-1.364491000	0.772463000
1	5.961368000	-1.438114000	-0.876589000
1	5.350973000	0.090700000	-0.262088000
17	2.953698000	1.408034000	-0.556486000
6	3.076449000	-0.141433000	1.671602000
1	4.106464000	0.207772000	1.749571000
1	2.464486000	0.509566000	2.298424000
1	2.994728000	-1.153866000	2.067491000
	f		
29	-0.350568000	0.215537000	0.252640000
6	-2.286779000	-0.220604000	0.494433000
6	2.473927000	3.512912000	-0.139554000
6	2.409251000	-0.718762000	-0.028411000
6	3.528101000	-1.532821000	-0.140314000
6	3.365787000	-2.908923000	-0.097698000
6	2.096475000	-3.439105000	0.051397000
6	1.028443000	-2.562347000	0.155606000
7	1.185600000	-1.241368000	0.118941000
1	4.514326000	-1.113916000	-0.261671000
1	4.226534000	-3.557378000	-0.183133000
1	1.928927000	-4.505053000	0.085563000
1	0.011588000	-2.919189000	0.264490000
6	2.483806000	0.771204000	-0.069675000
6	3.685835000	1.454978000	-0.196629000
6	3.679500000	2.839402000	-0.231789000
6	1.317515000	2.763629000	-0.013346000
7	1.318692000	1.431781000	0.021009000
1	4.620503000	0.921989000	-0.266284000
1	4.608547000	3.383350000	-0.329958000
1	2.422982000	4.590929000	-0.163118000
1	0.351066000	3.243532000	0.063364000
1	-2.954701000	1.076274000	-0.171790000
6	-3.721758000	1.901974000	-0.040642000
1	-3.371324000	2.643816000	-0.758001000
1	-3.578182000	2.273283000	0.971819000
6	-5.129693000	1.433144000	-0.318374000
1	-5.431231000	0.625124000	0.348131000
1	-5.822917000	2.258962000	-0.157483000
1	-5.238326000	1.091983000	-1.344032000
6	-2.739114000	-1.217379000	-0.503819000
8	-2.421718000	-2.363823000	-0.309244000

8	-3.372930000	-0.775210000	-1.582396000
1	-3.536816000	-1.530840000	-2.168061000
6	-2.944920000	-0.346895000	1.815274000
1	-4.032492000	-0.429807000	1.780390000
1	-2.573839000	-1.318287000	2.173758000
1	-2.629275000	0.411595000	2.524172000
g			
29	-0.657258000	-0.495118000	-0.466428000
6	-2.680558000	-0.609132000	-0.685253000
6	2.849650000	-3.037874000	0.031576000
6	1.797348000	1.063554000	-0.024881000
6	2.685340000	2.106600000	0.201714000
6	2.217249000	3.410746000	0.163525000
6	0.878232000	3.642818000	-0.095715000
6	0.053569000	2.549296000	-0.305889000
7	0.501988000	1.296460000	-0.271955000
1	3.725842000	1.917923000	0.412904000
1	2.895402000	4.234342000	0.338075000
1	0.472994000	4.642757000	-0.133071000
1	-1.003436000	2.677190000	-0.504130000
6	2.216160000	-0.368890000	-0.000919000
6	3.542503000	-0.754444000	0.145456000
6	3.861088000	-2.102012000	0.162131000
6	1.553149000	-2.578227000	-0.115926000
7	1.241775000	-1.282384000	-0.132871000
1	4.325133000	-0.018613000	0.237711000
1	4.889977000	-2.414429000	0.273585000
1	3.052810000	-4.098049000	0.039153000
1	0.729120000	-3.270343000	-0.225945000
1	-2.848400000	-1.235347000	0.696295000
6	-3.250138000	-1.085645000	1.763621000
1	-3.296649000	-2.115173000	2.118127000
1	-4.246650000	-0.661273000	1.677070000
6	-2.261958000	-0.217145000	2.500201000
1	-1.265314000	-0.659240000	2.496103000
1	-2.211048000	0.782560000	2.067803000
1	-2.574114000	-0.107229000	3.538975000
6	-3.217408000	0.693770000	-0.465646000
7	-3.537337000	1.791427000	-0.313849000
6	-3.546486000	-1.516286000	-1.481090000
1	-4.611278000	-1.448608000	-1.261341000
1	-3.409048000	-1.145756000	-2.508836000
1	-3.196286000	-2.543571000	-1.462750000

h			
29	0.470038000	-0.353938000	0.132609000
6	2.550653000	-0.124670000	0.303234000
6	-2.715371000	-3.329019000	-0.091088000
6	-2.194990000	0.872374000	-0.004658000
6	-3.229190000	1.794099000	-0.104057000
6	-2.932299000	3.147219000	-0.069489000
6	-1.614506000	3.548738000	0.060189000
6	-0.639225000	2.568934000	0.147573000
7	-0.922335000	1.269537000	0.117104000
1	-4.253070000	1.474483000	-0.214475000
1	-3.725629000	3.877487000	-0.146787000
1	-1.340864000	4.592655000	0.089420000
1	0.407938000	2.826991000	0.239698000
6	-2.426719000	-0.602118000	-0.034523000
6	-3.701052000	-1.153549000	-0.073023000
6	-3.845624000	-2.530429000	-0.102469000
6	-1.479977000	-2.707571000	-0.046460000
7	-1.335751000	-1.383161000	-0.018770000
1	-4.576373000	-0.524131000	-0.074057000
1	-4.832140000	-2.971489000	-0.131855000
1	-2.781105000	-4.406286000	-0.113233000
1	-0.568253000	-3.289405000	-0.031311000
1	2.542982000	-1.115140000	-0.312562000
6	3.845414000	-1.376896000	-0.678577000
1	3.692469000	-1.136138000	-1.726902000
1	3.609571000	-2.419032000	-0.453414000
6	5.168085000	-0.952349000	-0.124340000
1	5.257934000	-1.204724000	0.929480000
1	5.945029000	-1.500489000	-0.660544000
1	5.350065000	0.110203000	-0.279826000
17	2.890578000	1.310589000	-0.700152000
7	3.047721000	-0.119773000	1.583035000
1	2.373260000	-0.041701000	2.325984000
1	3.888615000	0.416119000	1.753840000
i			
29	-0.326206000	-0.540411000	-0.235688000
6	-2.404222000	-0.302942000	-0.470912000
6	3.138093000	-3.148043000	0.110638000
6	2.176544000	0.976607000	-0.015066000
6	3.112060000	2.001724000	0.036905000
6	2.674997000	3.316164000	-0.013702000
6	1.320175000	3.577442000	-0.115456000

6	0.448193000	2.501515000	-0.165644000
7	0.867840000	1.239473000	-0.116207000
1	4.167209000	1.792992000	0.112337000
1	3.390611000	4.125506000	0.025770000
1	0.939586000	4.586912000	-0.156439000
1	-0.622633000	2.648150000	-0.241875000
6	2.560296000	-0.466306000	0.034860000
6	3.878349000	-0.878441000	0.185085000
6	4.169081000	-2.231762000	0.222899000
6	1.850270000	-2.663659000	-0.033442000
7	1.565686000	-1.362162000	-0.070066000
1	4.675562000	-0.158438000	0.275759000
1	5.191281000	-2.563480000	0.339901000
1	3.318564000	-4.212052000	0.134711000
1	1.011229000	-3.340192000	-0.123016000
1	-2.522359000	-1.436243000	-0.155339000
6	-3.831502000	-1.729687000	0.121426000
1	-3.606166000	-2.054498000	1.133168000
1	-3.761952000	-2.533579000	-0.610838000
6	-5.073777000	-0.909591000	-0.020799000
1	-5.147816000	-0.480205000	-1.018771000
1	-5.933089000	-1.570316000	0.110147000
1	-5.139010000	-0.125127000	0.731179000
6	-2.662622000	0.650073000	0.626595000
8	-2.878725000	1.820007000	0.439163000
8	-2.576520000	0.105623000	1.853713000
1	-2.720675000	0.811915000	2.499285000
7	-2.817289000	0.085333000	-1.717328000
1	-2.195790000	-0.036699000	-2.496858000
1	-3.391883000	0.915169000	-1.762294000
j			
29	0.532970000	0.362679000	-0.098456000
6	2.653319000	0.335625000	-0.329609000
6	-2.717501000	3.282913000	0.067920000
6	-2.094609000	-0.908135000	0.041451000
6	-3.113286000	-1.849739000	0.090866000
6	-2.788226000	-3.197363000	0.083973000
6	-1.458442000	-3.572420000	0.029011000
6	-0.496918000	-2.575439000	-0.019165000
7	-0.808588000	-1.280874000	-0.013593000
1	-4.148343000	-1.551349000	0.134966000
1	-3.570523000	-3.942485000	0.122414000
1	-1.161619000	-4.610341000	0.023123000
1	0.557921000	-2.815803000	-0.060802000
6	-2.361034000	0.561489000	0.050106000
6	-3.647688000	1.083064000	0.107870000
6	-3.826422000	2.455892000	0.116518000
6	-1.468485000	2.691588000	0.011135000
7	-1.291512000	1.370638000	0.001899000
1	-4.506883000	0.433196000	0.145980000
1	-4.823048000	2.872278000	0.161046000
1	-2.810457000	4.358385000	0.073334000
1	-0.572032000	3.295826000	-0.028175000
1	2.649838000	0.974089000	0.651661000
6	3.929220000	0.844924000	1.222957000
1	3.705919000	0.067164000	1.946743000
1	3.723167000	1.842393000	1.616863000
6	5.249387000	0.740556000	0.534530000
1	5.322393000	1.452740000	-0.285695000
1	6.031597000	0.990929000	1.253842000
1	5.435676000	-0.269791000	0.172344000
6	2.838329000	-1.070926000	-0.127126000
7	2.959458000	-2.207329000	0.009113000
7	3.147101000	0.898561000	-1.478667000
1	2.579996000	1.534032000	-2.008620000
1	3.899225000	0.451903000	-1.977703000
P			
a			
29	0.924345000	-0.589003000	-0.188698000
6	3.100247000	-1.287483000	-0.358280000
1	2.544570000	-1.355203000	0.599829000
1	3.562836000	-2.267977000	-0.462252000
6	-0.281564000	3.624713000	-0.040883000
6	-1.929705000	-0.278739000	0.057551000
6	-3.286863000	-0.545809000	0.182454000
6	-3.722043000	-1.860858000	0.202704000
6	-2.795134000	-2.882766000	0.098342000
6	-1.459765000	-2.540344000	-0.021466000
7	-1.038342000	-1.276761000	-0.041251000
1	-4.005614000	0.253143000	0.265428000
1	-4.776034000	-2.080562000	0.299573000
1	-3.090602000	-3.920970000	0.109454000
1	-0.697233000	-3.302997000	-0.105145000
6	-1.390368000	1.115238000	0.026769000
6	-2.216237000	2.229642000	0.096924000
6	-1.655173000	3.495675000	0.062925000

6	0.477114000	2.469495000	-0.107822000
7	-0.058945000	1.250384000	-0.074958000
1	-3.286078000	2.124345000	0.175226000
1	-2.288530000	4.370059000	0.116326000
1	0.197549000	4.591594000	-0.070973000
1	1.554419000	2.519013000	-0.191326000
1	2.455395000	-1.191289000	-1.254110000
6	4.133727000	-0.168122000	-0.292030000
1	3.627869000	0.796217000	-0.208237000
1	4.688589000	-0.145152000	-1.230034000
6	5.089616000	-0.355298000	0.877648000
1	5.623479000	-1.301947000	0.793356000
1	5.828205000	0.442769000	0.912100000
1	4.553061000	-0.357366000	1.827478000
		b	
29	0.419008000	0.621666000	-0.362540000
6	3.517745000	-0.019822000	-0.353558000
6	-0.631721000	-3.627055000	-0.053624000
6	-2.412563000	0.212892000	0.113171000
6	-3.760704000	0.437505000	0.360670000
6	-4.239225000	1.737364000	0.383579000
6	-3.363721000	2.785247000	0.160160000
6	-2.033452000	2.483522000	-0.074878000
7	-1.570834000	1.235144000	-0.097311000
1	-4.437734000	-0.382385000	0.538215000
1	-5.286352000	1.925452000	0.575253000
1	-3.694497000	3.812801000	0.168157000
1	-1.308873000	3.267117000	-0.251647000
6	-1.826817000	-1.161384000	0.066720000
6	-2.603237000	-2.303858000	0.212410000
6	-1.997453000	-3.548301000	0.153167000
6	0.076996000	-2.445863000	-0.193726000
7	-0.503388000	-1.248521000	-0.132474000
1	-3.668208000	-2.236500000	0.365444000
1	-2.590977000	-4.444913000	0.264827000
1	-0.120832000	-4.576446000	-0.109551000
1	1.145148000	-2.447234000	-0.367200000
1	4.506020000	0.310424000	-0.666874000
6	3.439014000	-0.426506000	1.092432000
1	3.987918000	-1.369402000	1.152848000
1	2.394414000	-0.652169000	1.325697000
17	2.516716000	1.573210000	-0.592212000
6	4.021903000	0.591855000	2.059135000
1	4.028681000	0.186366000	3.068025000
1	5.049762000	0.839099000	1.793347000
1	3.441421000	1.512228000	2.068190000
8	2.975357000	-0.983974000	-1.151743000
1	3.214817000	-0.845471000	-2.074975000
		c	
29	0.339950000	-0.176834000	-0.677162000
6	3.348828000	0.518744000	0.583728000
6	-2.720542000	-3.261043000	0.055212000
6	-2.257411000	0.946993000	0.015534000
6	-3.287439000	1.848411000	0.253800000
6	-3.027140000	3.207573000	0.201701000
6	-1.745110000	3.639712000	-0.089582000
6	-0.772981000	2.682371000	-0.320105000
7	-1.018083000	1.374127000	-0.268416000
1	-4.286112000	1.504548000	0.469879000
1	-3.820701000	3.918673000	0.383766000
1	-1.498111000	4.689277000	-0.142682000
1	0.242198000	2.971201000	-0.558292000
6	-2.467517000	-0.531543000	0.050911000
6	-3.680458000	-1.102608000	0.415044000
6	-3.806006000	-2.482242000	0.415946000
6	-1.543218000	-2.618361000	-0.287992000
7	-1.419156000	-1.293273000	-0.288609000
1	-4.520784000	-0.490594000	0.700894000
1	-4.743975000	-2.940476000	0.696666000
1	-2.776315000	-4.339061000	0.039564000
1	-0.664924000	-3.181779000	-0.574998000
1	2.546077000	1.264583000	0.493111000
6	3.088423000	-0.351490000	1.819375000
1	3.270335000	0.306130000	2.668734000
1	3.857962000	-1.124031000	1.850216000
6	1.696227000	-0.958396000	1.908718000
1	1.574300000	-1.470260000	2.861288000
1	0.920862000	-0.188142000	1.856113000
1	1.514844000	-1.695565000	1.124383000
6	3.265693000	-0.331655000	-0.667041000
8	2.212683000	-0.635772000	-1.208267000
8	4.422109000	-0.743401000	-1.128143000
1	4.279570000	-1.297113000	-1.911826000
8	4.601450000	1.102143000	0.767729000
1	4.868870000	1.612269000	-0.001987000
		d	

29	0.205389000	0.058383000	-0.200920000
6	4.781668000	-0.108969000	-0.535444000
6	-2.314403000	-3.509482000	0.286406000
6	-2.630662000	0.702033000	0.008714000
6	-3.825708000	1.407337000	0.066805000
6	-3.803316000	2.788520000	-0.034931000
6	-2.590360000	3.435737000	-0.192416000
6	-1.441976000	2.664524000	-0.241347000
7	-1.459784000	1.336669000	-0.143691000
1	-4.767000000	0.896251000	0.189554000
1	-4.726053000	3.349966000	0.008989000
1	-2.527362000	4.510176000	-0.275795000
1	-0.469744000	3.122972000	-0.363089000
6	-2.572451000	-0.786431000	0.111052000
6	-3.708682000	-1.569082000	0.270536000
6	-3.575771000	-2.945150000	0.358907000
6	-1.230259000	-2.663516000	0.126742000
7	-1.354687000	-1.340942000	0.041228000
1	-4.688470000	-1.122652000	0.326230000
1	-4.451336000	-3.566826000	0.483359000
1	-2.166137000	-4.576817000	0.351239000
1	-0.223600000	-3.055102000	0.064930000
1	5.109059000	0.698214000	-1.197855000
6	5.393734000	0.084224000	0.848302000
1	6.468822000	-0.025826000	0.712304000
1	5.061619000	-0.739767000	1.480216000
6	5.053765000	1.432420000	1.465698000
1	5.548466000	1.540266000	2.427970000
1	5.383757000	2.253758000	0.828986000
1	3.981473000	1.540007000	1.633306000
6	3.298377000	-0.002748000	-0.445005000
7	2.159199000	0.048183000	-0.365411000
8	5.130350000	-1.374302000	-1.019435000
1	5.050575000	-1.407186000	-1.977174000

e

29	-0.343548000	-0.265595000	0.181791000
6	-3.587538000	0.282586000	-0.611941000
6	1.780548000	3.572644000	0.137401000
6	2.534311000	-0.591802000	-0.055804000
6	3.794864000	-1.147254000	-0.232507000
6	3.927645000	-2.525782000	-0.275244000
6	2.801845000	-3.319176000	-0.143011000
6	1.578446000	-2.692248000	0.021177000
7	1.448431000	-1.368124000	0.062686000
1	4.667343000	-0.523932000	-0.344762000
1	4.902801000	-2.971549000	-0.412843000
1	2.860977000	-4.396773000	-0.169194000
1	0.667488000	-3.267151000	0.123069000
6	2.313157000	0.884392000	0.008432000
6	3.366350000	1.790167000	0.021278000
6	3.096169000	3.147009000	0.085031000
6	0.785303000	2.611418000	0.127402000
7	1.040455000	1.305831000	0.063859000
1	4.389203000	1.450849000	-0.007674000
1	3.907738000	3.860990000	0.097240000
1	1.524184000	4.619979000	0.189596000
1	-0.258152000	2.894262000	0.172687000
1	-3.636965000	-0.209082000	-1.580874000
6	-4.954556000	0.362068000	0.033095000
1	-5.507550000	1.110210000	-0.541624000
1	-4.846471000	0.760005000	1.044062000
17	-2.555785000	-0.899477000	0.382858000
6	-5.721560000	-0.951899000	0.038969000
1	-6.719028000	-0.806760000	0.447690000
1	-5.827988000	-1.344553000	-0.972632000
1	-5.219319000	-1.704604000	0.644722000
6	-2.856983000	1.601457000	-0.689065000
1	-3.453744000	2.298373000	-1.278599000
1	-2.722820000	2.022129000	0.308066000
1	-1.886670000	1.495555000	-1.176550000

f

29	0.222684000	-0.678546000	-0.130244000
6	3.550157000	-0.282006000	0.481994000
6	-3.680819000	-2.703746000	0.086710000
6	-2.021750000	1.188968000	-0.035516000
6	-2.770040000	2.359306000	0.001567000
6	-2.122940000	3.582673000	-0.040880000
6	-0.741522000	3.613757000	-0.117526000
6	-0.065287000	2.407024000	-0.147127000
7	-0.683091000	1.227755000	-0.108339000
1	-3.845688000	2.327547000	0.065571000
1	-2.694759000	4.499618000	-0.012823000
1	-0.195461000	4.544340000	-0.152950000
1	1.015896000	2.381232000	-0.203142000
6	-2.655000000	-0.163369000	0.006653000
6	-4.032067000	-0.340393000	0.048886000

6	-4.548008000	-1.625554000	0.089755000
6	-2.321109000	-2.446179000	0.040357000
7	-1.823223000	-1.212748000	0.001954000
1	-4.703393000	0.503181000	0.047554000
1	-5.617619000	-1.778605000	0.122690000
1	-4.041002000	-3.720987000	0.117617000
1	-1.600860000	-3.253592000	0.033629000
1	4.588942000	-0.451536000	0.764010000
6	3.031538000	-1.567677000	-0.091567000
8	1.851599000	-1.815479000	-0.299867000
8	3.967486000	-2.434903000	-0.411020000
1	3.561314000	-3.217102000	-0.815896000
6	3.509916000	0.762212000	-0.651503000
1	2.478915000	0.840044000	-1.014204000
1	4.107153000	0.397953000	-1.489049000
6	2.741604000	0.138702000	1.707781000
1	3.244752000	0.954498000	2.221444000
1	1.745426000	0.491622000	1.427489000
1	2.628276000	-0.681221000	2.415433000
6	4.022879000	2.127046000	-0.213523000
1	4.046903000	2.813092000	-1.058309000
1	3.394328000	2.569140000	0.559502000
1	5.035239000	2.052044000	0.183947000
	g		
29	0.208478000	-0.003498000	-0.174816000
6	4.760455000	0.015723000	-0.530632000
6	-2.427761000	-3.484128000	0.290242000
6	-2.614101000	0.733873000	-0.005187000
6	-3.787907000	1.475300000	0.033916000
6	-3.721492000	2.854686000	-0.073925000
6	-2.487222000	3.463468000	-0.218278000
6	-1.362421000	2.656925000	-0.248335000
7	-1.422729000	1.331155000	-0.144804000
1	-4.746024000	0.993922000	0.146468000
1	-4.626933000	3.444548000	-0.045117000
1	-2.390181000	4.535047000	-0.305913000
1	-0.374719000	3.084132000	-0.359361000
6	-2.601380000	-0.755067000	0.103639000
6	-3.762536000	-1.502960000	0.250625000
6	-3.672482000	-2.881997000	0.344740000
6	-1.316855000	-2.671460000	0.142564000
7	-1.399764000	-1.345947000	0.051783000
1	-4.728981000	-1.026858000	0.292100000

1	-4.567952000	-3.476649000	0.459290000
1	-2.312359000	-4.555225000	0.359622000
1	-0.321824000	-3.093378000	0.094753000
1	5.025439000	0.945846000	-1.040417000
6	5.384230000	0.021963000	0.875075000
1	6.464711000	-0.008505000	0.732386000
1	5.106023000	-0.902758000	1.383601000
6	3.301401000	0.023809000	-0.422825000
7	2.161344000	0.007540000	-0.326784000
6	5.210386000	-1.182656000	-1.373358000
1	4.760823000	-1.169403000	-2.363687000
1	6.292175000	-1.145499000	-1.482928000
1	4.946182000	-2.116151000	-0.878972000
6	4.992913000	1.234050000	1.706563000
1	5.485794000	1.209258000	2.676008000
1	5.281694000	2.159908000	1.208338000
1	3.916957000	1.264801000	1.883184000
	h		
29	-0.364455000	0.345666000	-0.083150000
6	-4.360199000	0.128496000	-0.455830000
6	3.021331000	3.173618000	0.180543000
6	2.242734000	-0.978685000	-0.059386000
6	3.223707000	-1.962509000	-0.085649000
6	2.850659000	-3.293530000	-0.162525000
6	1.505707000	-3.616233000	-0.211657000
6	0.587542000	-2.581376000	-0.183096000
7	0.941473000	-1.299815000	-0.109722000
1	4.269293000	-1.702489000	-0.047794000
1	3.604797000	-4.067747000	-0.183814000
1	1.169648000	-4.640383000	-0.271450000
1	-0.475100000	-2.781675000	-0.219521000
6	2.573477000	0.475444000	0.025663000
6	3.879111000	0.943672000	0.101848000
6	4.101817000	2.309133000	0.179863000
6	1.750023000	2.628847000	0.102634000
7	1.535332000	1.318971000	0.027499000
1	4.717356000	0.265464000	0.102754000
1	5.111778000	2.689917000	0.239946000
1	3.152317000	4.243571000	0.240319000
1	0.868917000	3.257543000	0.100492000
1	-3.928777000	-0.221070000	-1.383711000
6	-4.396570000	-0.798726000	0.714804000
1	-5.327998000	-1.361844000	0.588357000

1	-4.501376000	-0.213777000	1.629564000
17	-2.408033000	1.301496000	-0.051500000
6	-3.227739000	-1.767067000	0.805326000
1	-3.441509000	-2.537696000	1.542603000
1	-3.060243000	-2.258716000	-0.154265000
1	-2.316688000	-1.251698000	1.101333000
7	-5.293405000	1.046775000	-0.569892000
1	-5.356047000	1.616809000	-1.398638000
1	-5.774024000	1.386033000	0.250257000
i			
29	-0.056895000	-0.296638000	0.165377000
6	-4.412069000	-0.518566000	0.429052000
6	1.960045000	3.603433000	-0.114504000
6	2.838749000	-0.536725000	0.007873000
6	4.121952000	-1.067173000	-0.027590000
6	4.290695000	-2.440566000	0.034821000
6	3.176540000	-3.255343000	0.132490000
6	1.930445000	-2.653707000	0.162147000
7	1.764324000	-1.333672000	0.100001000
1	4.986080000	-0.426638000	-0.099719000
1	5.284171000	-2.865950000	0.009334000
1	3.262451000	-4.330089000	0.186527000
1	1.029416000	-3.247166000	0.240862000
6	2.579030000	0.933333000	-0.045891000
6	3.601781000	1.867597000	-0.149800000
6	3.286671000	3.216099000	-0.184958000
6	0.997299000	2.613857000	-0.014318000
7	1.296654000	1.317031000	0.016727000
1	4.633626000	1.559692000	-0.202689000
1	4.072652000	3.953926000	-0.265188000
1	1.671021000	4.643356000	-0.135380000
1	-0.053463000	2.864973000	0.046078000
1	-4.207197000	-0.608884000	1.495829000
6	-4.989861000	0.874204000	0.144314000
1	-5.911885000	0.938697000	0.719858000
1	-5.267004000	0.919417000	-0.911360000
6	-4.048778000	2.018688000	0.495095000
1	-4.549989000	2.975633000	0.367568000
1	-3.712220000	1.948713000	1.530002000
1	-3.164998000	2.026621000	-0.146553000
6	-3.063178000	-0.614657000	-0.252349000
8	-2.001267000	-0.516328000	0.342040000
8	-3.132787000	-0.780599000	-1.560926000

1	-2.246144000	-0.811559000	-1.947629000
7	-5.359823000	-1.533687000	0.035598000
1	-5.049349000	-2.470553000	0.254191000
1	-5.578788000	-1.482846000	-0.951131000
j			
29	-0.206529000	0.089541000	-0.171100000
6	-4.783564000	0.124905000	-0.537421000
6	2.596347000	3.430576000	0.300291000
6	2.576136000	-0.790695000	-0.010210000
6	3.713125000	-1.588006000	0.019268000
6	3.579199000	-2.962274000	-0.091740000
6	2.316067000	-3.510288000	-0.229567000
6	1.231523000	-2.650128000	-0.251006000
7	1.356693000	-1.329225000	-0.144658000
1	4.694252000	-1.154063000	0.126376000
1	4.455234000	-3.595303000	-0.070619000
1	2.166951000	-4.575725000	-0.319063000
1	0.223597000	-3.028656000	-0.357274000
6	2.635401000	0.696758000	0.103672000
6	3.831703000	1.386261000	0.254102000
6	3.809878000	2.767637000	0.353138000
6	1.446885000	2.674237000	0.149445000
7	1.464026000	1.346341000	0.053721000
1	4.773374000	0.862915000	0.295373000
1	4.733478000	3.317031000	0.470382000
1	2.533794000	4.505780000	0.373611000
1	0.474109000	3.145203000	0.103169000
1	-5.044084000	-0.721310000	-1.176915000
6	-5.411081000	-0.086282000	0.843564000
1	-6.487892000	-0.054992000	0.684444000
1	-5.154198000	0.767166000	1.474828000
6	-4.991491000	-1.390524000	1.504428000
1	-5.498780000	-1.512194000	2.458715000
1	-5.248471000	-2.246281000	0.879518000
1	-3.918114000	-1.418300000	1.696155000
6	-3.300710000	0.093534000	-0.413907000
7	-2.159875000	0.115857000	-0.324021000
7	-5.265530000	1.338826000	-1.150796000
1	-4.917279000	1.478556000	-2.089303000
1	-5.060353000	2.156599000	-0.591555000

Reaction with propane

R

a

29	0.019142000	1.427097000	-0.869056000
6	0.524791000	3.188551000	-1.338009000
1	0.975699000	3.924955000	-0.666522000
1	0.437406000	3.660226000	-2.320386000
6	2.558456000	-2.106614000	-1.011983000
6	-1.382492000	-0.950657000	-0.007034000
6	-2.371836000	-1.801198000	0.464523000
6	-3.628988000	-1.287717000	0.741513000
6	-3.869925000	0.060027000	0.541334000
6	-2.833649000	0.846248000	0.067118000
7	-1.625134000	0.355340000	-0.199830000
1	-2.175722000	-2.851060000	0.613445000
1	-4.410481000	-1.937818000	1.109202000
1	-4.834578000	0.499780000	0.744601000
1	-2.970692000	1.905042000	-0.104901000
6	-0.004331000	-1.412614000	-0.331703000
6	0.416525000	-2.722343000	-0.147023000
6	1.712773000	-3.070784000	-0.491758000
6	2.070971000	-0.819286000	-1.156179000
7	0.826322000	-0.481652000	-0.824752000
1	-0.249257000	-3.466300000	0.260599000
1	2.055136000	-4.086799000	-0.353810000
1	3.574392000	-2.336607000	-1.295309000
1	2.695863000	-0.026966000	-1.546081000
6	2.760809000	-0.047353000	2.112567000
1	2.758337000	-0.141347000	3.198772000
1	2.099311000	-0.822732000	1.716778000
1	3.771750000	-0.252442000	1.761933000
6	2.284445000	1.337344000	1.693207000
1	2.948649000	2.096602000	2.108202000
1	2.356656000	1.436934000	0.605067000
6	0.854828000	1.603809000	2.149099000
1	0.180391000	0.818784000	1.795530000
1	0.791568000	1.607862000	3.237498000
1	0.477868000	2.565767000	1.796060000
b			
29	0.724092000	0.049702000	-0.691456000
6	2.621657000	0.100393000	-1.308292000
6	-1.925295000	3.487122000	-0.060720000
6	-2.055030000	-0.736766000	-0.276083000
6	-3.203673000	-1.501356000	-0.121111000
6	-3.109760000	-2.882591000	-0.173915000
6	-1.873672000	-3.469803000	-0.379082000

6	-0.774894000	-2.640746000	-0.527107000
7	-0.861548000	-1.313359000	-0.477723000
1	-4.162573000	-1.036640000	0.043323000
1	-3.995410000	-3.490581000	-0.053393000
1	-1.755554000	-4.541862000	-0.424699000
1	0.213082000	-3.050700000	-0.690173000
6	-2.066020000	0.753747000	-0.208910000
6	-3.229294000	1.485372000	-0.010412000
6	-3.155928000	2.866885000	0.063963000
6	-0.811236000	2.690225000	-0.262108000
7	-0.878093000	1.362795000	-0.335158000
1	-4.184414000	0.994657000	0.086764000
1	-4.053278000	3.449357000	0.219028000
1	-1.822870000	4.560371000	-0.006127000
1	0.172910000	3.127059000	-0.368149000
1	1.498946000	0.772183000	1.664272000
6	1.371165000	-0.156038000	2.234725000
1	1.463593000	-0.988805000	1.526606000
6	-0.013940000	-0.178041000	2.868568000
1	-0.125925000	0.645293000	3.574568000
1	-0.804533000	-0.089770000	2.123289000
1	-0.170831000	-1.107149000	3.417298000
17	3.957595000	0.054185000	-0.220325000
6	2.480683000	-0.260992000	3.273841000
1	3.469456000	-0.249397000	2.817228000
1	2.425416000	0.569465000	3.977765000
1	2.384479000	-1.185340000	3.843643000
8	2.983536000	0.172319000	-2.533249000
1	3.950341000	0.190277000	-2.677464000
c			
29	-0.366410000	0.195657000	-0.702448000
6	1.451403000	0.489297000	-1.399945000
6	-3.251747000	3.236803000	0.564716000
6	-2.866345000	-0.963514000	0.247757000
6	-3.848352000	-1.879369000	0.600750000
6	-3.599620000	-3.232929000	0.442237000
6	-2.378744000	-3.641956000	-0.064804000
6	-1.450258000	-2.669283000	-0.393593000
7	-1.685367000	-1.367619000	-0.241658000
1	-4.797538000	-1.552076000	0.993806000
1	-4.354715000	-3.957799000	0.712416000
1	-2.144369000	-4.686240000	-0.205711000
1	-0.482192000	-2.937884000	-0.794477000

6	-3.053251000	0.509717000	0.382878000
6	-4.222720000	1.075389000	0.872695000
6	-4.320857000	2.454213000	0.964003000
6	-2.119224000	2.599466000	0.087879000
7	-2.020907000	1.274595000	-0.000304000
1	-5.051575000	0.458418000	1.180764000
1	-5.225306000	2.908992000	1.343095000
1	-3.286709000	4.314430000	0.617781000
1	-1.257518000	3.166728000	-0.237195000
1	5.877187000	-1.674060000	2.027731000
6	5.630776000	-0.879585000	1.322321000
1	4.736477000	-1.218012000	0.789578000
6	6.766496000	-0.692467000	0.325566000
1	7.678865000	-0.383196000	0.836229000
1	6.980674000	-1.614050000	-0.213165000
1	6.513147000	0.077219000	-0.404323000
6	5.341713000	0.404316000	2.094843000
1	4.523060000	0.287924000	2.812787000
1	6.212118000	0.710274000	2.674716000
1	5.124330000	1.234025000	1.413871000
6	2.728073000	0.561110000	-0.525776000
8	3.796978000	0.743456000	-1.043511000
8	2.472998000	0.404257000	0.750409000
1	3.300007000	0.451930000	1.267412000
8	1.803857000	0.650508000	-2.608897000
1	2.789134000	0.777483000	-2.663771000

d

29	-0.783266000	-0.036176000	-0.738264000
6	-2.624272000	-0.073760000	-1.488621000
6	1.831295000	-3.482289000	-0.053721000
6	1.970653000	0.743981000	-0.218020000
6	3.111740000	1.506758000	-0.010417000
6	3.015784000	2.888945000	-0.031708000
6	1.784832000	3.479178000	-0.257900000
6	0.693146000	2.652593000	-0.461457000
7	0.782455000	1.324139000	-0.444644000
1	4.066300000	1.039985000	0.172465000
1	3.895707000	3.495358000	0.131239000
1	1.664763000	4.551827000	-0.276871000
1	-0.290761000	3.065441000	-0.641063000
6	1.980500000	-0.747207000	-0.174645000
6	3.134237000	-1.481995000	0.061875000
6	3.056788000	-2.864186000	0.121924000
6	0.726322000	-2.683128000	-0.292152000
7	0.797833000	-1.354878000	-0.353659000
1	4.084939000	-0.993224000	0.202489000
1	3.946860000	-3.448849000	0.307650000
1	1.725506000	-4.555659000	-0.008761000
1	-0.253742000	-3.118360000	-0.436107000
1	-1.679824000	-0.824846000	1.556049000
6	-1.567574000	0.092802000	2.148317000
1	-1.666761000	0.943322000	1.460813000
6	-0.189803000	0.117738000	2.799092000
1	-0.075617000	-0.723932000	3.482741000
1	0.615265000	0.065214000	2.066368000
1	-0.057503000	1.032153000	3.378145000
6	-2.686383000	0.157704000	3.181604000
1	-3.670316000	0.141510000	2.717839000
1	-2.618504000	-0.687947000	3.866246000
1	-2.602663000	1.069860000	3.772788000
6	-3.745698000	-0.055038000	-0.581040000
7	-4.536815000	-0.036919000	0.252555000
8	-3.040310000	-0.114282000	-2.706186000
1	-2.300153000	-0.127445000	-3.331462000

e

29	0.760305000	0.027636000	-0.686418000
6	2.647162000	0.032556000	-1.259912000
6	-1.903292000	3.471934000	-0.183961000
6	-2.026829000	-0.754923000	-0.290846000
6	-3.175831000	-1.519803000	-0.141339000
6	-3.073788000	-2.901578000	-0.144644000
6	-1.829673000	-3.488303000	-0.295732000
6	-0.731241000	-2.658721000	-0.444368000
7	-0.825863000	-1.330980000	-0.443650000
1	-4.140910000	-1.054629000	-0.019312000
1	-3.959374000	-3.510287000	-0.027320000
1	-1.705153000	-4.560616000	-0.299809000
1	0.262882000	-3.068056000	-0.566559000
6	-2.042411000	0.736138000	-0.267141000
6	-3.207753000	1.471564000	-0.097541000
6	-3.135172000	2.854532000	-0.056406000
6	-0.787189000	2.670855000	-0.355317000
7	-0.853426000	1.342063000	-0.397659000
1	-4.163141000	0.982404000	0.005408000
1	-4.033851000	3.440365000	0.076768000
1	-1.801431000	4.546190000	-0.153044000

1	0.198347000	3.104917000	-0.460388000
1	1.397907000	0.861226000	1.688871000
6	1.269482000	-0.033207000	2.310320000
1	1.401474000	-0.902843000	1.655458000
6	-0.134417000	-0.047389000	2.901336000
1	-0.289873000	0.819652000	3.544076000
1	-0.902695000	-0.033330000	2.128105000
1	-0.286608000	-0.939867000	3.509092000
17	3.875920000	-0.023391000	-0.111430000
6	2.345799000	-0.051407000	3.388852000
1	3.348352000	-0.041215000	2.963528000
1	2.248374000	0.816346000	4.041494000
1	2.251982000	-0.943668000	4.008120000
6	3.157318000	0.115091000	-2.635007000
1	4.186882000	-0.208462000	-2.777563000
1	2.469359000	-0.365001000	-3.330022000
1	3.100028000	1.191450000	-2.867074000
	f		
29	0.535837000	0.308001000	-0.759483000
6	2.395625000	0.497221000	-1.308623000
6	-2.421903000	3.345015000	0.290509000
6	-2.104767000	-0.828589000	-0.320886000
6	-3.142914000	-1.737296000	-0.168714000
6	-2.890262000	-3.087519000	-0.351896000
6	-1.610335000	-3.500303000	-0.678732000
6	-0.627263000	-2.534534000	-0.813517000
7	-0.869266000	-1.236836000	-0.642445000
1	-4.134981000	-1.408639000	0.097032000
1	-3.687667000	-3.807998000	-0.235075000
1	-1.370880000	-4.542695000	-0.824970000
1	0.391457000	-2.803027000	-1.061889000
6	-2.276529000	0.638147000	-0.115120000
6	-3.503177000	1.216429000	0.180174000
6	-3.574463000	2.585060000	0.384720000
6	-1.236548000	2.698200000	-0.014851000
7	-1.165010000	1.383590000	-0.213955000
1	-4.396434000	0.616412000	0.250147000
1	-4.522840000	3.049370000	0.616166000
1	-2.432692000	4.413168000	0.446216000
1	-0.308696000	3.247301000	-0.102682000
1	1.461260000	0.548628000	1.676035000
6	1.302934000	-0.443410000	2.117070000
1	1.303191000	-1.170395000	1.298305000

6	-0.046032000	-0.467536000	2.824392000
1	-0.061859000	0.243233000	3.651118000
1	-0.862775000	-0.210755000	2.148686000
1	-0.247114000	-1.457337000	3.235364000
6	2.964330000	0.735493000	-2.632227000
1	3.846083000	1.384496000	-2.600389000
1	3.362023000	-0.256650000	-2.915738000
1	2.249910000	1.052201000	-3.383107000
6	2.457656000	-0.762138000	3.059260000
1	3.407011000	-0.827387000	2.527465000
1	2.543540000	-0.004634000	3.838612000
1	2.296354000	-1.723379000	3.547687000
6	3.414717000	0.164170000	-0.299004000
8	3.733043000	-0.986432000	-0.168518000
8	3.862458000	1.199239000	0.397515000
1	4.495359000	0.885857000	1.063535000
	g		
29	0.793555000	0.058956000	-0.745239000
6	2.622321000	0.135173000	-1.411938000
6	-1.789688000	3.494299000	0.021135000
6	-1.956295000	-0.722330000	-0.265944000
6	-3.099790000	-1.488160000	-0.087618000
6	-3.005643000	-2.869293000	-0.149248000
6	-1.774357000	-3.454864000	-0.385808000
6	-0.679649000	-2.625250000	-0.558347000
7	-0.767680000	-1.298008000	-0.502348000
1	-4.054196000	-1.024299000	0.103732000
1	-3.887375000	-3.478809000	-0.009363000
1	-1.656208000	-4.526685000	-0.436103000
1	0.304699000	-3.033580000	-0.744834000
6	-1.959580000	0.765743000	-0.179394000
6	-3.110151000	1.502277000	0.064300000
6	-3.022029000	2.881620000	0.164695000
6	-0.687710000	2.693917000	-0.227372000
7	-0.770072000	1.368827000	-0.327305000
1	-4.066046000	1.016638000	0.178844000
1	-3.909496000	3.468337000	0.356286000
1	-1.676167000	4.565077000	0.097552000
1	0.297321000	3.124301000	-0.348612000
1	1.637871000	0.733445000	1.683114000
6	1.487712000	-0.211787000	2.219424000
1	1.586990000	-1.021583000	1.485349000
6	0.091366000	-0.240399000	2.828839000

1	-0.020518000	0.556287000	3.564842000
1	-0.689396000	-0.113402000	2.078835000
1	-0.084843000	-1.187425000	3.340043000
6	3.660662000	0.126747000	-0.459454000
7	4.461487000	0.129301000	0.373484000
6	3.076310000	0.168435000	-2.805211000
1	4.001986000	0.723162000	-2.967115000
1	3.337183000	-0.890637000	-2.996763000
1	2.296323000	0.437017000	-3.509560000
6	2.574722000	-0.364293000	3.276925000
1	3.570483000	-0.344096000	2.839094000
1	2.508222000	0.441544000	4.008193000
1	2.454738000	-1.306870000	3.811671000
		h	
29	0.754315000	0.059713000	-0.698135000
6	2.671143000	0.114849000	-1.304842000
6	-1.943506000	3.474254000	-0.078406000
6	-2.039305000	-0.749523000	-0.303431000
6	-3.181949000	-1.523648000	-0.148953000
6	-3.074256000	-2.904281000	-0.188748000
6	-1.830955000	-3.480958000	-0.380256000
6	-0.739690000	-2.642011000	-0.529905000
7	-0.839537000	-1.315295000	-0.494233000
1	-4.146019000	-1.066818000	0.007535000
1	-3.954452000	-3.519955000	-0.067205000
1	-1.701805000	-4.552245000	-0.413100000
1	0.253952000	-3.043164000	-0.681238000
6	-2.063166000	0.741155000	-0.239993000
6	-3.234404000	1.463717000	-0.053306000
6	-3.171610000	2.845298000	0.027547000
6	-0.821887000	2.685469000	-0.270678000
7	-0.878542000	1.358165000	-0.351646000
1	-4.187090000	0.965848000	0.031118000
1	-4.074704000	3.420998000	0.174432000
1	-1.848914000	4.547800000	-0.015556000
1	0.161147000	3.128842000	-0.360767000
1	1.411512000	0.798000000	1.683348000
6	1.287937000	-0.125373000	2.262143000
1	1.405783000	-0.962964000	1.564008000
6	-0.106289000	-0.162523000	2.874669000
1	-0.244598000	0.669304000	3.566063000
1	-0.886797000	-0.100665000	2.116286000
1	-0.255280000	-1.085752000	3.435601000
17	3.920741000	0.010164000	-0.121601000
6	2.381496000	-0.201567000	3.320442000
1	3.376518000	-0.177796000	2.878650000
1	2.300908000	0.634844000	4.015003000
1	2.291098000	-1.121678000	3.898193000
7	3.158896000	0.218574000	-2.503666000
1	4.155858000	0.234168000	-2.700213000
1	2.528071000	0.287490000	-3.289903000
		i	
29	-0.604689000	0.217818000	-0.845889000
6	-2.580675000	0.234146000	-1.173889000
6	2.350252000	-3.020667000	-1.050376000
6	2.076163000	1.064438000	0.009157000
6	3.146769000	1.850261000	0.415133000
6	2.921318000	3.175082000	0.751896000
6	1.636155000	3.683463000	0.676812000
6	0.623691000	2.838712000	0.253555000
7	0.837751000	1.567251000	-0.074779000
1	4.146082000	1.447064000	0.460130000
1	3.743775000	3.801430000	1.067924000
1	1.417188000	4.708995000	0.933404000
1	-0.398241000	3.185921000	0.175514000
6	2.226454000	-0.373531000	-0.356066000
6	3.404576000	-1.078607000	-0.142422000
6	3.465133000	-2.416752000	-0.494494000
6	1.211756000	-2.252692000	-1.224264000
7	1.149013000	-0.966333000	-0.887536000
1	4.261707000	-0.602062000	0.306382000
1	4.372972000	-2.980248000	-0.330627000
1	2.353605000	-4.061204000	-1.337979000
1	0.311812000	-2.682095000	-1.645040000
1	-1.101011000	-2.038071000	0.662120000
6	-0.937765000	-1.653927000	1.674714000
1	-0.776421000	-0.573883000	1.584966000
6	0.306912000	-2.302276000	2.268549000
1	0.183424000	-3.383498000	2.337768000
1	1.196801000	-2.103235000	1.669313000
1	0.491511000	-1.926433000	3.275407000
6	-2.180515000	-1.911692000	2.518403000
1	-3.079206000	-1.486519000	2.069873000
1	-2.349489000	-2.982273000	2.636659000
1	-2.065590000	-1.484868000	3.515274000
6	-3.508531000	0.537565000	0.005007000

8	-4.673561000	0.261755000	0.020922000
8	-2.848341000	1.136329000	0.991432000
1	-3.464971000	1.307157000	1.720333000
7	-3.310801000	0.013150000	-2.210874000
1	-2.903529000	-0.221361000	-3.105327000
1	-4.331112000	0.034180000	-2.142111000
j			
29	0.791718000	0.071090000	-0.740396000
6	2.670272000	0.141192000	-1.459580000
6	-1.844260000	3.489783000	0.056082000
6	-1.981208000	-0.727722000	-0.258653000
6	-3.122727000	-1.496448000	-0.073612000
6	-3.027909000	-2.876924000	-0.142409000
6	-1.797793000	-3.459306000	-0.392526000
6	-0.706493000	-2.625991000	-0.570060000
7	-0.794294000	-1.299270000	-0.507525000
1	-4.076101000	-1.035763000	0.129794000
1	-3.907553000	-3.488181000	0.003010000
1	-1.678300000	-4.530748000	-0.448387000
1	0.277112000	-3.032425000	-0.765956000
6	-1.991087000	0.761470000	-0.163475000
6	-3.148628000	1.487815000	0.082804000
6	-3.072244000	2.866795000	0.192904000
6	-0.736564000	2.698383000	-0.196269000
7	-0.806654000	1.373602000	-0.305872000
1	-4.101379000	0.994851000	0.191403000
1	-3.964792000	3.445012000	0.386779000
1	-1.738984000	4.560815000	0.140889000
1	0.245556000	3.137521000	-0.312139000
1	1.672967000	0.727705000	1.634953000
6	1.539720000	-0.223799000	2.165127000
1	1.633071000	-1.026851000	1.422554000
6	0.153579000	-0.268821000	2.796470000
1	0.047341000	0.520957000	3.540752000
1	-0.639179000	-0.142351000	2.059349000
1	-0.006364000	-1.221493000	3.302555000
6	2.645117000	-0.376454000	3.203295000
1	3.633662000	-0.344732000	2.749990000
1	2.583789000	0.422701000	3.942387000
1	2.540865000	-1.324442000	3.731809000
6	3.743595000	0.126485000	-0.504718000
7	4.539002000	0.113514000	0.324998000
7	3.078131000	0.201395000	-2.689868000
1	4.057955000	0.234492000	-2.957187000
1	2.407001000	0.217886000	-3.445485000
TS			
a			
29	-0.661842000	-0.783929000	-0.229389000
6	-2.390462000	-1.795271000	-0.387828000
6	3.306521000	-2.641441000	0.153940000
6	1.529330000	1.190205000	-0.025176000
6	2.234379000	2.387630000	-0.028471000
6	1.540488000	3.582786000	-0.112660000
6	0.158974000	3.556844000	-0.195335000
6	-0.468923000	2.323486000	-0.190966000
7	0.191886000	1.171100000	-0.106629000
1	3.311437000	2.396441000	0.023070000
1	2.075679000	4.521986000	-0.118025000
1	-0.421903000	4.464085000	-0.265731000
1	-1.547257000	2.253270000	-0.259057000
6	2.205607000	-0.136554000	0.052878000
6	3.573426000	-0.269503000	0.253790000
6	4.127972000	-1.538090000	0.303865000
6	1.951522000	-2.425449000	-0.034200000
7	1.415144000	-1.208634000	-0.082275000
1	4.204489000	0.596043000	0.378403000
1	5.190758000	-1.659289000	0.460068000
1	3.697697000	-3.647212000	0.184213000
1	1.266840000	-3.254926000	-0.151509000
1	-3.457058000	-1.363897000	0.157414000
6	-3.877872000	-0.240111000	0.010449000
1	-3.427590000	0.237848000	-0.850068000
6	-3.552651000	0.444374000	1.313798000
1	-2.480712000	0.591914000	1.434881000
1	-4.042345000	1.419820000	1.318250000
1	-3.931756000	-0.121264000	2.163466000
6	-5.337222000	-0.576509000	-0.215560000
1	-5.886242000	0.362774000	-0.303597000
1	-5.483411000	-1.140985000	-1.133574000
1	-5.751816000	-1.133875000	0.622625000
1	-2.819669000	-2.048991000	-1.358125000
1	-2.559463000	-2.651458000	0.280768000
b			
29	-0.353440000	0.095775000	-0.043653000
6	-2.338457000	-0.551762000	-0.199982000
6	2.305942000	-3.425779000	0.174025000

6	2.464683000	0.800517000	0.044523000
6	3.636593000	1.546053000	0.038233000
6	3.563662000	2.928029000	-0.010526000
6	2.322336000	3.538285000	-0.053453000
6	1.199396000	2.729969000	-0.046331000
7	1.264486000	1.400057000	0.001983000
1	4.599711000	1.062372000	0.066651000
1	4.468916000	3.518843000	-0.016829000
1	2.218589000	4.612021000	-0.093736000
1	0.207434000	3.159826000	-0.082712000
6	2.467288000	-0.691915000	0.093810000
6	3.635227000	-1.438096000	0.183208000
6	3.549589000	-2.820830000	0.222437000
6	1.187533000	-2.612716000	0.088709000
7	1.269117000	-1.286068000	0.049206000
1	4.601547000	-0.961446000	0.225153000
1	4.449128000	-3.416393000	0.291533000
1	2.197506000	-4.499530000	0.202620000
1	0.188688000	-3.028192000	0.053238000
1	-2.549006000	0.351430000	0.466039000
6	-3.963100000	0.331575000	0.658709000
1	-4.583332000	-0.383346000	0.126237000
6	-4.175087000	1.751418000	0.207443000
1	-4.129366000	1.845313000	-0.875116000
1	-5.166331000	2.074819000	0.530788000
1	-3.449075000	2.425842000	0.665163000
6	-3.899419000	0.099385000	2.143503000
1	-4.892624000	0.262171000	2.565757000
1	-3.596121000	-0.918380000	2.379599000
1	-3.218051000	0.800896000	2.626895000
8	-2.438015000	-1.799546000	0.317750000
1	-3.241571000	-2.247824000	0.009974000
17	-2.813205000	-0.408367000	-1.884795000
c			
29	-0.235392000	0.006540000	-0.080240000
6	-2.234872000	-0.555830000	-0.247596000
6	2.529125000	-3.435176000	0.074140000
6	2.568215000	0.795523000	0.055966000
6	3.716491000	1.576860000	0.077708000
6	3.599382000	2.956672000	0.056892000
6	2.339681000	3.528386000	0.012356000
6	1.242644000	2.685085000	-0.009854000
7	1.350987000	1.357709000	0.012999000

1	4.694228000	1.123096000	0.104837000
1	4.485291000	3.575998000	0.072393000
1	2.203195000	4.599052000	-0.007680000
1	0.236559000	3.081406000	-0.050597000
6	2.613318000	-0.696911000	0.070521000
6	3.800108000	-1.411724000	0.166155000
6	3.753545000	-2.796937000	0.167052000
6	1.389631000	-2.652029000	-0.013563000
7	1.433483000	-1.322791000	-0.014697000
1	4.750859000	-0.908596000	0.242597000
1	4.668073000	-3.368685000	0.240630000
1	2.451036000	-4.511944000	0.070400000
1	0.404528000	-3.095196000	-0.084806000
1	-2.401338000	0.079021000	0.726836000
6	-3.728567000	0.201608000	1.131042000
1	-4.488711000	-0.052887000	0.399332000
6	-3.629987000	1.671956000	1.432816000
1	-3.562127000	2.272603000	0.530163000
1	-4.534753000	1.965675000	1.970435000
1	-2.779661000	1.884808000	2.082017000
6	-3.681191000	-0.737176000	2.298011000
1	-4.631438000	-0.661279000	2.831294000
1	-3.560536000	-1.769729000	1.978579000
1	-2.883431000	-0.471242000	2.991327000
6	-2.912134000	-0.007157000	-1.453264000
8	-3.481658000	-0.720808000	-2.235621000
8	-2.793926000	1.318884000	-1.591454000
1	-3.258176000	1.575829000	-2.402091000
8	-2.417064000	-1.888281000	-0.113965000
1	-3.109661000	-2.169272000	-0.738810000
d			
29	0.399352000	-0.233688000	0.318032000
6	2.438356000	0.058029000	0.699991000
6	-2.546449000	-3.434248000	0.006163000
6	-2.311166000	0.793052000	-0.124819000
6	-3.395261000	1.633207000	-0.340867000
6	-3.194056000	3.004515000	-0.354485000
6	-1.919868000	3.504232000	-0.154009000
6	-0.887780000	2.602532000	0.050737000
7	-1.079856000	1.285460000	0.063603000
1	-4.385453000	1.237437000	-0.500409000
1	-4.028020000	3.671910000	-0.521149000
1	-1.720135000	4.565158000	-0.155494000

1	0.128192000	2.942461000	0.209099000
6	-2.442510000	-0.694106000	-0.088322000
6	-3.668293000	-1.336290000	-0.211115000
6	-3.719218000	-2.719282000	-0.164160000
6	-1.364051000	-2.725579000	0.123844000
7	-1.310474000	-1.395058000	0.077121000
1	-4.578461000	-0.772195000	-0.336317000
1	-4.667465000	-3.230004000	-0.257110000
1	-2.540594000	-4.512790000	0.050929000
1	-0.421744000	-3.237992000	0.263998000
1	2.701299000	-0.529036000	-0.284570000
6	4.012076000	-0.464733000	-0.681846000
1	4.667722000	0.116062000	-0.041938000
6	3.791504000	0.141710000	-2.035774000
1	3.527401000	1.195861000	-1.970481000
1	4.728476000	0.069865000	-2.593309000
1	3.030610000	-0.399677000	-2.597831000
6	4.238311000	-1.945782000	-0.588708000
1	5.251251000	-2.156969000	-0.938345000
1	4.163522000	-2.294059000	0.440531000
1	3.541933000	-2.498358000	-1.218474000
8	2.882830000	-0.528642000	1.841920000
1	3.628610000	-0.052775000	2.233307000
6	2.635022000	1.474072000	0.558369000
7	2.723087000	2.615769000	0.443459000
		e	
29	-0.366429000	-0.106006000	-0.260160000
6	-2.336884000	0.454273000	-0.500149000
6	2.269258000	-3.576035000	-0.015258000
6	2.466301000	0.652250000	0.068428000
6	3.633370000	1.383569000	0.247662000
6	3.566384000	2.767740000	0.256112000
6	2.340874000	3.387837000	0.088220000
6	1.222147000	2.588005000	-0.080036000
7	1.284537000	1.259748000	-0.089975000
1	4.584295000	0.893788000	0.384281000
1	4.465149000	3.352237000	0.394717000
1	2.245592000	4.463238000	0.089173000
1	0.237903000	3.020175000	-0.207881000
6	2.446439000	-0.839911000	0.043975000
6	3.605007000	-1.601668000	0.131217000
6	3.514319000	-2.982981000	0.101994000
6	1.161703000	-2.751246000	-0.100800000
7	1.243303000	-1.421811000	-0.072085000
1	4.571244000	-1.130849000	0.215793000
1	4.408634000	-3.586656000	0.168182000
1	2.151938000	-4.648797000	-0.042932000
1	0.167768000	-3.167272000	-0.197775000
1	-2.657394000	-0.464522000	0.176986000
6	-3.946401000	-0.664037000	0.434349000
1	-4.586373000	0.158040000	0.137355000
6	-3.757213000	-0.773484000	1.923572000
1	-3.444900000	0.173047000	2.359841000
1	-4.716786000	-1.045318000	2.368111000
1	-3.038572000	-1.552811000	2.178078000
6	-4.183742000	-1.949238000	-0.305921000
1	-5.084532000	-2.412844000	0.102570000
1	-4.343575000	-1.795108000	-1.369444000
1	-3.362151000	-2.651369000	-0.156039000
17	-2.600575000	1.965410000	0.377235000
6	-2.902546000	0.504135000	-1.888419000
1	-2.799160000	-0.461164000	-2.380686000
1	-3.943773000	0.835039000	-1.929944000
1	-2.308062000	1.222518000	-2.453992000
		f	
29	-0.419965000	-0.474606000	-0.243089000
6	-2.479667000	-0.493423000	-0.489712000
6	1.286311000	3.606571000	0.308199000
6	2.496247000	-0.432962000	-0.033658000
6	3.817525000	-0.862213000	-0.031737000
6	4.094249000	-2.213262000	-0.156832000
6	3.046781000	-3.109066000	-0.282675000
6	1.757580000	-2.606592000	-0.279839000
7	1.486447000	-1.307666000	-0.158508000
1	4.627998000	-0.157050000	0.059502000
1	5.118503000	-2.558970000	-0.158271000
1	3.216428000	-4.170317000	-0.384255000
1	0.905487000	-3.265622000	-0.380916000
6	2.123187000	1.007284000	0.095576000
6	3.064586000	2.009117000	0.296211000
6	2.637577000	3.323131000	0.402522000
6	0.409054000	2.551693000	0.112934000
7	0.817945000	1.289329000	0.010426000
1	4.115712000	1.781315000	0.374083000
1	3.356999000	4.114866000	0.558318000
1	0.913095000	4.616777000	0.384845000

1	-0.659637000	2.717377000	0.034656000
1	-3.291491000	-0.325156000	0.425377000
6	-3.013964000	-0.561144000	1.610069000
1	-4.018687000	-0.174341000	1.818333000
6	-2.910750000	-2.036255000	1.859530000
1	-3.018908000	-2.191776000	2.934499000
1	-3.695572000	-2.599706000	1.360897000
1	-1.935490000	-2.415251000	1.555605000
6	-1.963680000	0.311491000	2.234826000
1	-2.203680000	0.391587000	3.297457000
1	-0.972007000	-0.132930000	2.149680000
1	-1.968880000	1.310797000	1.809185000
6	-2.700632000	0.882563000	-0.991396000
8	-2.969579000	1.855489000	-0.325594000
8	-2.471427000	0.947038000	-2.313651000
1	-2.585167000	1.869250000	-2.585142000
6	-3.103393000	-1.638452000	-1.253769000
1	-2.688048000	-1.576450000	-2.261566000
1	-2.816560000	-2.606010000	-0.849556000
1	-4.189851000	-1.587574000	-1.344121000
	g		
29	0.539173000	0.299098000	-0.587987000
6	2.563406000	0.167157000	-0.876943000
6	-1.392756000	-3.625086000	0.170413000
6	-2.315797000	0.499453000	0.001603000
6	-3.583228000	1.034448000	0.195777000
6	-3.764951000	2.404114000	0.105118000
6	-2.678393000	3.213523000	-0.178617000
6	-1.447498000	2.608756000	-0.360031000
7	-1.267312000	1.291057000	-0.272102000
1	-4.425441000	0.396806000	0.411416000
1	-4.746914000	2.831447000	0.252734000
1	-2.775298000	4.285490000	-0.261249000
1	-0.568428000	3.197354000	-0.587465000
6	-2.044950000	-0.966855000	0.077525000
6	-3.031503000	-1.896727000	0.377266000
6	-2.697674000	-3.241457000	0.423055000
6	-0.464309000	-2.637590000	-0.119438000
7	-0.783864000	-1.346218000	-0.163055000
1	-4.046830000	-1.591533000	0.574147000
1	-3.453848000	-3.978775000	0.653825000
1	-1.092239000	-4.661754000	0.193936000
1	0.570340000	-2.884094000	-0.325142000

1	2.911988000	0.499111000	0.338747000
6	3.234192000	0.805915000	1.461244000
1	4.318872000	0.829924000	1.367811000
6	2.703224000	-0.330146000	2.281573000
1	3.137651000	-1.283482000	1.984861000
1	2.963113000	-0.153943000	3.328165000
1	1.615640000	-0.386204000	2.208312000
6	2.589816000	2.148730000	1.637571000
1	2.834331000	2.520076000	2.635581000
1	2.953255000	2.876042000	0.914107000
1	1.503801000	2.069797000	1.566387000
6	2.933904000	-1.213577000	-0.801028000
7	3.124726000	-2.347633000	-0.713412000
6	3.439487000	1.016886000	-1.748989000
1	3.221182000	2.074078000	-1.622311000
1	4.507197000	0.826164000	-1.642629000
1	3.162951000	0.753948000	-2.776211000
	h		
29	-0.377697000	-0.112267000	-0.262594000
6	-2.391196000	0.408863000	-0.514916000
6	2.282131000	-3.561459000	0.019023000
6	2.437866000	0.669679000	0.061708000
6	3.596080000	1.412807000	0.248642000
6	3.517254000	2.796400000	0.244879000
6	2.288599000	3.404681000	0.057984000
6	1.178462000	2.594342000	-0.115756000
7	1.252499000	1.266263000	-0.114617000
1	4.549063000	0.932226000	0.402393000
1	4.409295000	3.389616000	0.389737000
1	2.184112000	4.479179000	0.049601000
1	0.191761000	3.016584000	-0.256351000
6	2.432687000	-0.823029000	0.050782000
6	3.599139000	-1.572276000	0.138647000
6	3.521724000	-2.954803000	0.123522000
6	1.165683000	-2.748956000	-0.069319000
7	1.235089000	-1.418525000	-0.053920000
1	4.561324000	-1.091338000	0.211734000
1	4.422368000	-3.548922000	0.190393000
1	2.175399000	-4.635557000	0.002759000
1	0.175438000	-3.175591000	-0.157831000
1	-2.517327000	-0.521107000	0.151060000
6	-3.897712000	-0.661893000	0.369917000
1	-4.588957000	0.095625000	0.016325000

6	-3.791663000	-0.721361000	1.870781000
1	-3.642112000	0.261107000	2.310261000
1	-4.726270000	-1.127257000	2.262801000
1	-2.988528000	-1.389751000	2.186552000
6	-4.020956000	-1.979550000	-0.341710000
1	-5.013714000	-2.388837000	-0.147269000
1	-3.908671000	-1.859259000	-1.416691000
1	-3.289698000	-2.699772000	0.028671000
17	-2.593897000	1.923294000	0.407815000
7	-2.863070000	0.404216000	-1.813244000
1	-2.162512000	0.479273000	-2.532897000
1	-3.661722000	0.999189000	-2.001518000
		i	
29	-0.285760000	-0.281657000	-0.237553000
6	-2.279282000	0.384595000	-0.444355000
6	2.599656000	-3.523577000	0.038744000
6	2.471086000	0.711262000	-0.033819000
6	3.592011000	1.530300000	0.004238000
6	3.422487000	2.905745000	-0.025103000
6	2.144209000	3.430774000	-0.091782000
6	1.075526000	2.549142000	-0.131203000
7	1.238556000	1.228619000	-0.103072000
1	4.586489000	1.116190000	0.051139000
1	4.284749000	3.557224000	0.003251000
1	1.970190000	4.496023000	-0.114736000
1	0.053223000	2.904934000	-0.180253000
6	2.562457000	-0.779614000	-0.003388000
6	3.774213000	-1.445835000	0.130380000
6	3.792126000	-2.830182000	0.151003000
6	1.431983000	-2.792625000	-0.087528000
7	1.409837000	-1.460260000	-0.107853000
1	4.698403000	-0.898505000	0.222755000
1	4.729456000	-3.358607000	0.255049000
1	2.566914000	-4.602508000	0.049669000
1	0.474831000	-3.288618000	-0.176157000
1	-2.580934000	-0.705840000	-0.214577000
6	-3.968961000	-0.830417000	0.148583000
1	-4.427515000	0.016242000	0.645261000
6	-3.608056000	-1.953397000	1.086981000
1	-3.043275000	-1.599663000	1.945214000
1	-4.533915000	-2.404135000	1.451807000
1	-3.047507000	-2.734807000	0.570479000
6	-4.606702000	-1.208838000	-1.144724000

1	-5.642718000	-1.492167000	-0.945176000
1	-4.618016000	-0.375358000	-1.844054000
1	-4.106509000	-2.061536000	-1.604672000
6	-2.305275000	1.319533000	0.692441000
8	-2.293793000	2.517815000	0.559242000
8	-2.283239000	0.723303000	1.901167000
1	-2.276802000	1.424019000	2.568585000
7	-2.615039000	0.923888000	-1.664368000
1	-2.024564000	0.732304000	-2.454469000
1	-2.977564000	1.866996000	-1.639091000
		j	
29	0.430378000	-0.197738000	0.342798000
6	2.499705000	0.083896000	0.720364000
6	-2.447383000	-3.469315000	-0.003409000
6	-2.299838000	0.763681000	-0.104538000
6	-3.405250000	1.581408000	-0.294807000
6	-3.232005000	2.956723000	-0.313712000
6	-1.963930000	3.481632000	-0.144099000
6	-0.908843000	2.601916000	0.040498000
7	-1.074912000	1.281025000	0.059844000
1	-4.391285000	1.166249000	-0.428246000
1	-4.083016000	3.606958000	-0.460926000
1	-1.785021000	4.546230000	-0.153191000
1	0.103941000	2.960823000	0.174450000
6	-2.400061000	-0.726092000	-0.073918000
6	-3.609025000	-1.393885000	-0.227033000
6	-3.631689000	-2.777712000	-0.191114000
6	-1.283412000	-2.736377000	0.142278000
7	-1.256891000	-1.404395000	0.108875000
1	-4.527629000	-0.848709000	-0.372247000
1	-4.566656000	-3.307535000	-0.308517000
1	-2.419115000	-4.547891000	0.031079000
1	-0.332872000	-3.230812000	0.292293000
1	2.621893000	-0.546424000	-0.245525000
6	3.938114000	-0.419180000	-0.742394000
1	4.634382000	0.214147000	-0.204897000
6	3.552360000	0.106730000	-2.094408000
1	3.255008000	1.153115000	-2.053299000
1	4.423122000	0.036210000	-2.750079000
1	2.755301000	-0.488237000	-2.542595000
6	4.236384000	-1.881708000	-0.607176000
1	5.233050000	-2.072026000	-1.009700000
1	4.244391000	-2.176903000	0.441969000

1	3.524486000	-2.491681000	-1.163415000
7	3.045630000	-0.421281000	1.887010000
1	2.457117000	-0.980118000	2.479202000
1	3.706602000	0.151961000	2.389001000
6	2.562302000	1.493621000	0.478173000
7	2.574852000	2.629937000	0.292168000
P			
a			
29	-0.774193000	-0.486992000	-0.134411000
6	-2.893494000	-1.438433000	-0.305445000
1	-2.249043000	-1.305746000	-1.195869000
1	-3.245946000	-2.466873000	-0.360493000
6	0.720929000	3.628648000	0.017511000
6	2.111819000	-0.374866000	0.044638000
6	3.453037000	-0.728540000	0.120516000
6	3.803371000	-2.068720000	0.121101000
6	2.809760000	-3.028756000	0.046222000
6	1.495942000	-2.600401000	-0.026663000
7	1.156056000	-1.312675000	-0.027644000
1	4.224430000	0.022005000	0.178805000
1	4.843891000	-2.355899000	0.179590000
1	3.038597000	-4.083731000	0.043827000
1	0.683256000	-3.312130000	-0.086688000
6	1.662200000	1.050943000	0.038458000
6	2.560294000	2.107977000	0.109862000
6	2.084124000	3.408735000	0.099163000
6	-0.113438000	2.526669000	-0.050120000
7	0.340739000	1.274696000	-0.040115000
1	3.621628000	1.931313000	0.173475000
1	2.774738000	4.238553000	0.154062000
1	0.305726000	4.625039000	0.006146000
1	-1.186508000	2.647157000	-0.114656000
1	-2.344975000	-1.382941000	0.658361000
6	-4.024207000	-0.414105000	-0.300471000
1	-4.588132000	-0.537710000	-1.227086000
6	-4.957062000	-0.658276000	0.879724000
1	-5.782695000	0.051675000	0.875080000
1	-5.376406000	-1.663515000	0.853981000
1	-4.419710000	-0.539641000	1.823637000
6	-3.449520000	0.996941000	-0.267091000
1	-4.237431000	1.748693000	-0.284169000
1	-2.874494000	1.145321000	0.654744000
1	-2.795439000	1.180174000	-1.124926000

b			
29	0.086200000	0.532265000	0.594208000
6	3.464577000	-0.130306000	0.047475000
6	-1.994393000	-3.286370000	-0.068925000
6	-2.752160000	0.879248000	0.047423000
6	-3.997393000	1.453570000	-0.172140000
6	-4.126802000	2.831831000	-0.116015000
6	-3.013355000	3.606371000	0.156721000
6	-1.806059000	2.961370000	0.364445000
7	-1.679803000	1.637393000	0.311795000
1	-4.861282000	0.845368000	-0.386703000
1	-5.090067000	3.292207000	-0.285462000
1	-3.070492000	4.683155000	0.208591000
1	-0.905491000	3.520528000	0.580823000
6	-2.530685000	-0.597514000	-0.000964000
6	-3.563424000	-1.494122000	-0.244761000
6	-3.290968000	-2.851389000	-0.279963000
6	-1.019946000	-2.333475000	0.170869000
7	-1.277030000	-1.027520000	0.203062000
1	-4.571936000	-1.148597000	-0.404909000
1	-4.085926000	-3.559027000	-0.469357000
1	-1.737834000	-4.334814000	-0.086180000
1	0.006826000	-2.624850000	0.347992000
1	2.815823000	-0.972601000	-0.167550000
6	4.641972000	-0.479640000	0.931103000
1	4.231450000	-0.920231000	1.840331000
6	5.466793000	-1.538697000	0.192320000
1	6.291821000	-1.861398000	0.824657000
1	4.869525000	-2.415341000	-0.057563000
1	5.882580000	-1.133668000	-0.730084000
6	5.489374000	0.733996000	1.301843000
1	6.305787000	0.419397000	1.948843000
1	5.953129000	1.189630000	0.423016000
1	4.913703000	1.492463000	1.834125000
17	2.260618000	0.964760000	1.152695000
8	3.712164000	0.532116000	-1.090817000
1	4.399577000	1.196633000	-0.953299000
c			
29	-0.218961000	0.050600000	0.000077000
6	4.082796000	0.774267000	-0.143371000
6	-2.705600000	-3.555650000	0.045219000
6	-3.066504000	0.664612000	0.057108000
6	-4.275376000	1.347037000	0.098849000

6	-4.270837000	2.732415000	0.114210000
6	-3.062467000	3.406008000	0.088957000
6	-1.899301000	2.656306000	0.047659000
7	-1.900784000	1.325127000	0.030536000
1	-5.213858000	0.816992000	0.122512000
1	-5.204376000	3.276422000	0.147847000
1	-3.013793000	4.484377000	0.102406000
1	-0.929202000	3.135084000	0.029841000
6	-2.988456000	-0.827222000	0.046788000
6	-4.121195000	-1.631211000	0.061189000
6	-3.976719000	-3.008653000	0.059830000
6	-1.625275000	-2.691017000	0.028981000
7	-1.760306000	-1.365747000	0.027581000
1	-5.108616000	-1.198729000	0.073692000
1	-4.850514000	-3.645003000	0.071403000
1	-2.547202000	-4.623488000	0.046691000
1	-0.612087000	-3.070107000	0.018867000
1	4.509751000	1.517826000	-0.818916000
6	5.000928000	-0.468426000	-0.139889000
1	5.029982000	-0.839983000	-1.166658000
6	6.404859000	-0.049470000	0.279681000
1	7.070058000	-0.911644000	0.280735000
1	6.815926000	0.696177000	-0.400437000
1	6.391061000	0.375332000	1.282703000
6	4.453250000	-1.562571000	0.772068000
1	5.100306000	-2.437163000	0.735475000
1	4.426606000	-1.227934000	1.810878000
1	3.448983000	-1.883984000	0.487335000
6	2.728859000	0.395692000	-0.693225000
8	1.734876000	0.303405000	0.008014000
8	2.730459000	0.134505000	-1.984114000
1	1.851816000	-0.145246000	-2.279258000
8	3.948483000	1.385256000	1.103347000
1	3.428339000	0.819982000	1.683530000
d			
29	-0.155928000	0.047097000	-0.163153000
6	4.391862000	0.542002000	-0.560258000
6	-2.668939000	-3.545305000	-0.063658000
6	-2.999445000	0.672941000	0.062453000
6	-4.198457000	1.365809000	0.168586000
6	-4.179695000	2.750677000	0.197375000
6	-2.967197000	3.412968000	0.119694000
6	-1.814752000	2.652746000	0.016425000

7	-1.829439000	1.321933000	-0.011060000
1	-5.139629000	0.843357000	0.228709000
1	-5.105201000	3.303222000	0.279575000
1	-2.907494000	4.490694000	0.137976000
1	-0.842339000	3.122711000	-0.047163000
6	-2.934990000	-0.818225000	0.022747000
6	-4.070221000	-1.616065000	0.085741000
6	-3.933643000	-2.993722000	0.041945000
6	-1.585978000	-2.685225000	-0.121391000
7	-1.713489000	-1.360521000	-0.079481000
1	-5.052459000	-1.179138000	0.167134000
1	-4.808754000	-3.626585000	0.089508000
1	-2.517431000	-4.613468000	-0.101451000
1	-0.577202000	-3.066876000	-0.204795000
1	4.582161000	0.645845000	-1.630055000
6	5.142446000	-0.687775000	-0.024656000
1	4.723975000	-1.553852000	-0.543877000
6	6.621727000	-0.562699000	-0.372590000
1	7.166371000	-1.430305000	-0.004306000
1	6.774347000	-0.497711000	-1.449473000
1	7.050878000	0.327745000	0.086058000
6	4.929008000	-0.864612000	1.475945000
1	5.385682000	-1.795084000	1.807629000
1	5.408687000	-0.065078000	2.045206000
1	3.871483000	-0.899035000	1.743677000
6	2.921797000	0.343290000	-0.426428000
7	1.793807000	0.197999000	-0.310527000
8	4.754577000	1.757021000	0.027344000
1	4.794946000	1.654877000	0.984229000
e			
29	0.321720000	-0.128421000	-0.764084000
6	3.498118000	0.225309000	-0.245946000
6	-2.562230000	-3.339360000	0.076643000
6	-2.238067000	0.882066000	0.163305000
6	-3.288793000	1.735365000	0.475455000
6	-3.083107000	3.104849000	0.449740000
6	-1.834813000	3.594846000	0.108449000
6	-0.839349000	2.683342000	-0.197161000
7	-1.031363000	1.365709000	-0.168201000
1	-4.263314000	1.346490000	0.723515000
1	-3.893737000	3.779058000	0.688248000
1	-1.631628000	4.654419000	0.071040000
1	0.150373000	3.017576000	-0.479949000

6	-2.394166000	-0.603810000	0.161794000
6	-3.545336000	-1.230314000	0.621910000
6	-3.628285000	-2.612532000	0.577014000
6	-1.444474000	-2.644474000	-0.352507000
7	-1.361853000	-1.316538000	-0.309322000
1	-4.368231000	-0.658669000	1.020209000
1	-4.518242000	-3.113267000	0.931714000
1	-2.587064000	-4.417162000	0.020521000
1	-0.581791000	-3.165609000	-0.747071000
1	4.480800000	0.243642000	-0.711184000
6	3.517400000	-0.719115000	0.954234000
1	3.937736000	-1.662588000	0.601019000
6	4.461061000	-0.143804000	2.013870000
1	4.654526000	-0.893376000	2.778941000
1	5.419029000	0.152500000	1.585547000
1	4.019872000	0.724137000	2.504745000
6	2.143030000	-1.003276000	1.554014000
1	2.255531000	-1.578210000	2.472252000
1	1.612469000	-0.084558000	1.816412000
1	1.518807000	-1.598067000	0.884350000
17	2.465883000	-0.512460000	-1.594333000
6	3.007663000	1.633158000	0.007583000
1	3.695820000	2.134586000	0.687718000
1	2.972885000	2.202045000	-0.919124000
1	2.016862000	1.635369000	0.465471000
		f	
29	-0.135891000	0.434359000	-0.860512000
6	-3.049461000	-0.355621000	0.170590000
6	3.187721000	3.023024000	0.358013000
6	2.210125000	-1.078127000	-0.045865000
6	3.106979000	-2.115559000	0.176642000
6	2.686335000	-3.424527000	0.008708000
6	1.381519000	-3.671216000	-0.380907000
6	0.549626000	-2.585602000	-0.592370000
7	0.949084000	-1.325746000	-0.428991000
1	4.126367000	-1.915530000	0.465176000
1	3.374963000	-4.240680000	0.176799000
1	1.012633000	-4.675377000	-0.525854000
1	-0.476028000	-2.727521000	-0.907903000
6	2.595601000	0.356144000	0.116903000
6	3.819673000	0.737739000	0.651263000
6	4.117742000	2.085221000	0.770876000
6	1.986079000	2.566396000	-0.154996000

7	1.696426000	1.271742000	-0.272454000
1	4.534351000	0.001549000	0.982528000
1	5.066598000	2.395470000	1.185558000
1	3.379083000	4.082886000	0.432088000
1	1.224661000	3.260886000	-0.484407000
1	-2.141665000	-0.937339000	-0.024962000
6	-2.905211000	0.310281000	1.558983000
1	-3.789145000	0.938141000	1.698546000
6	-2.860391000	-0.740868000	2.662842000
1	-2.676876000	-0.264995000	3.625262000
1	-3.792096000	-1.295913000	2.742014000
1	-2.049754000	-1.451925000	2.482101000
6	-1.662043000	1.194108000	1.633090000
1	-1.605835000	1.694277000	2.598853000
1	-0.754497000	0.585048000	1.539012000
1	-1.651325000	1.974058000	0.867433000
6	-3.163073000	0.724740000	-0.870858000
8	-4.112826000	1.392482000	-1.113758000
8	-1.984560000	0.928549000	-1.586645000
1	-2.160015000	1.629815000	-2.232817000
6	-4.278021000	-1.258603000	0.061531000
1	-4.414114000	-1.616551000	-0.957773000
1	-4.173055000	-2.122158000	0.712829000
1	-5.172101000	-0.705939000	0.347006000
		g	
29	-0.145817000	-0.094015000	-0.220197000
6	4.389969000	-0.279846000	-0.698542000
6	-2.977201000	-3.441397000	0.023639000
6	-2.914917000	0.789930000	0.086826000
6	-4.041111000	1.592722000	0.214666000
6	-3.893657000	2.970022000	0.221017000
6	-2.627769000	3.515600000	0.099761000
6	-1.554475000	2.650125000	-0.024111000
7	-1.692566000	1.326160000	-0.030585000
1	-5.024412000	1.160486000	0.307893000
1	-4.761372000	3.607144000	0.319630000
1	-2.468126000	4.583259000	0.099920000
1	-0.544987000	3.026590000	-0.122281000
6	-2.989576000	-0.701060000	0.070947000
6	-4.189857000	-1.389673000	0.190421000
6	-4.181344000	-2.774606000	0.166278000
6	-1.823002000	-2.685305000	-0.089033000
7	-1.827557000	-1.354289000	-0.066013000

1	-5.124190000	-0.863284000	0.301551000
1	-5.108000000	-3.323675000	0.258235000
1	-2.925314000	-4.519408000	-0.000448000
1	-0.856973000	-3.158996000	-0.202097000
1	4.594813000	-1.159842000	-1.313819000
6	5.032543000	-0.507051000	0.695079000
1	4.558076000	-1.399668000	1.109627000
6	6.523548000	-0.789561000	0.545780000
1	6.941749000	-1.076766000	1.509298000
1	6.713347000	-1.600215000	-0.158178000
1	7.063522000	0.094805000	0.207562000
6	4.781907000	0.658234000	1.645638000
1	5.149500000	0.409755000	2.639962000
1	5.308917000	1.554517000	1.317666000
1	3.720251000	0.895090000	1.733412000
6	2.935687000	-0.230037000	-0.554893000
7	1.799788000	-0.179052000	-0.425390000
6	4.870528000	0.973343000	-1.441086000
1	4.635836000	1.875484000	-0.880262000
1	5.947152000	0.918305000	-1.581344000
1	4.403612000	1.044466000	-2.421012000
	h		
29	-0.141729000	0.057926000	-0.235227000
6	4.548044000	-0.191445000	-0.737473000
6	-2.591729000	-3.577432000	-0.052422000
6	-3.026957000	0.629406000	0.122414000
6	-4.236587000	1.292304000	0.290141000
6	-4.245397000	2.677192000	0.328973000
6	-3.052266000	3.366630000	0.200381000
6	-1.888756000	2.632215000	0.037445000
7	-1.879083000	1.303825000	0.000137000
1	-5.162556000	0.748844000	0.390552000
1	-5.177628000	3.209067000	0.458495000
1	-3.016784000	4.445402000	0.224987000
1	-0.926799000	3.117444000	-0.067648000
6	-2.923605000	-0.859333000	0.067141000
6	-4.035153000	-1.687495000	0.166009000
6	-3.865727000	-3.060339000	0.105413000
6	-1.534883000	-2.688064000	-0.143873000
7	-1.693476000	-1.367907000	-0.086112000
1	-5.024168000	-1.275600000	0.287543000
1	-4.721815000	-3.716201000	0.180331000
1	-2.414078000	-4.640987000	-0.104985000

1	-0.519856000	-3.040961000	-0.268862000
1	4.087019000	-0.965319000	-1.335980000
6	4.802687000	-0.433922000	0.703184000
1	3.897359000	-0.900600000	1.088098000
6	5.957438000	-1.452883000	0.781900000
1	6.117568000	-1.715087000	1.825712000
1	5.735379000	-2.367035000	0.232945000
1	6.880453000	-1.024803000	0.390975000
6	5.098626000	0.839012000	1.481966000
1	5.186852000	0.603461000	2.540214000
1	6.049854000	1.286271000	1.182323000
1	4.293656000	1.564007000	1.359903000
17	2.012460000	0.602988000	-0.486873000
7	5.037076000	0.819533000	-1.367942000
1	5.449317000	1.590795000	-0.858590000
1	4.878509000	0.947423000	-2.357382000
	i		
29	-0.233257000	-0.292957000	-0.082451000
6	4.104506000	-0.858009000	-0.061591000
6	-3.534236000	-3.179375000	-0.066381000
6	-2.845779000	0.998733000	-0.009308000
6	-3.849492000	1.958236000	0.025814000
6	-3.504811000	3.299896000	0.030039000
6	-2.167829000	3.655406000	-0.001612000
6	-1.225272000	2.642210000	-0.033641000
7	-1.553135000	1.351758000	-0.035654000
1	-4.889442000	1.675050000	0.049554000
1	-4.275928000	4.057009000	0.056912000
1	-1.855555000	4.688787000	-0.002131000
1	-0.167530000	2.868474000	-0.060585000
6	-3.138067000	-0.466125000	-0.023910000
6	-4.433815000	-0.966538000	-0.019408000
6	-4.631992000	-2.337256000	-0.040295000
6	-2.274227000	-2.606704000	-0.069405000
7	-2.079939000	-1.289237000	-0.047338000
1	-5.284876000	-0.304995000	-0.002690000
1	-5.635348000	-2.739594000	-0.037899000
1	-3.643200000	-4.253232000	-0.085504000
1	-1.384980000	-3.222354000	-0.092134000
1	4.589519000	-1.700161000	0.436148000
6	4.873255000	0.424193000	0.351879000
1	4.757593000	0.528122000	1.432444000
6	6.353041000	0.260660000	0.028972000

1	6.901384000	1.154562000	0.323604000
1	6.781759000	-0.589520000	0.559556000
1	6.492478000	0.103384000	-1.039176000
6	4.294180000	1.660437000	-0.330201000
1	4.799203000	2.555604000	0.029010000
1	4.445646000	1.612113000	-1.409416000
1	3.225582000	1.782825000	-0.136725000
6	2.704370000	-0.773541000	0.508227000
8	1.707016000	-0.600416000	-0.175341000
8	2.655709000	-0.872029000	1.823846000
1	1.745763000	-0.780406000	2.141132000
7	4.137765000	-1.065562000	-1.486037000
1	3.535810000	-0.412355000	-1.970528000
1	3.848957000	-2.000048000	-1.740409000
j			
29	-0.152022000	-0.121569000	-0.187385000
6	4.404574000	-0.494731000	-0.599522000
6	-3.047287000	-3.412219000	0.110125000
6	-2.906195000	0.817486000	0.063779000
6	-4.018861000	1.643672000	0.156450000
6	-3.846486000	3.017748000	0.129455000
6	-2.569373000	3.537234000	0.010953000
6	-1.510745000	2.649725000	-0.076912000
7	-1.672709000	1.328532000	-0.051709000
1	-5.011005000	1.231817000	0.247892000
1	-4.703571000	3.672716000	0.200212000
1	-2.390179000	4.601490000	-0.013901000
1	-0.493513000	3.005691000	-0.171189000
6	-3.008829000	-0.671818000	0.085513000
6	-4.223120000	-1.334729000	0.209197000
6	-4.240205000	-2.719761000	0.221313000
6	-1.877959000	-2.680777000	-0.008978000
7	-1.857950000	-1.349783000	-0.021022000
1	-5.148622000	-0.788605000	0.296279000
1	-5.177896000	-3.249104000	0.316987000
1	-3.015472000	-4.491275000	0.115068000
1	-0.919777000	-3.175084000	-0.098203000
1	4.604370000	-1.516493000	-0.927591000
6	5.053605000	-0.293140000	0.786917000
1	4.534530000	-0.966108000	1.473964000
6	6.523860000	-0.688560000	0.721668000
1	6.987152000	-0.557095000	1.698238000
1	6.643627000	-1.731680000	0.428822000

1	7.053697000	-0.066913000	0.001856000
6	4.879844000	1.140006000	1.278452000
1	5.247349000	1.229656000	2.299048000
1	5.457769000	1.833227000	0.665802000
1	3.833733000	1.453494000	1.274584000
6	2.927600000	-0.394934000	-0.461555000
7	1.792767000	-0.280306000	-0.364977000
7	4.929063000	0.415045000	-1.591012000
1	4.680653000	1.376935000	-1.402760000
1	4.645071000	0.177169000	-2.531037000
Reaction with isobutane			
R			
a			
29	-0.141046000	1.148808000	1.140048000
6	-1.147000000	2.564230000	1.891860000
1	-1.915821000	3.142503000	1.370682000
1	-1.092270000	2.949255000	2.913577000
6	-1.311304000	-3.043605000	1.025314000
6	1.832912000	-0.491745000	-0.191070000
6	2.972769000	-0.893196000	-0.872376000
6	3.934183000	0.050966000	-1.196459000
6	3.736926000	1.371448000	-0.833174000
6	2.575626000	1.696088000	-0.152774000
7	1.650635000	0.790944000	0.159837000
1	3.120173000	-1.925753000	-1.146194000
1	4.828071000	-0.246402000	-1.726649000
1	4.460483000	2.137775000	-1.066550000
1	2.374358000	2.713677000	0.152865000
6	0.748214000	-1.435113000	0.198573000
6	0.758043000	-2.780581000	-0.142676000
6	-0.284291000	-3.591792000	0.276840000
6	-1.258742000	-1.691111000	1.314462000
7	-0.258841000	-0.909420000	0.912020000
1	1.561159000	-3.197908000	-0.728978000
1	-0.290637000	-4.641615000	0.019207000
1	-2.140179000	-3.640818000	1.374012000
1	-2.043973000	-1.211593000	1.883476000
6	-2.695757000	-0.824914000	-1.695732000
1	-2.847563000	-0.793834000	-2.776503000
1	-1.779860000	-1.390614000	-1.507357000
1	-3.532130000	-1.371004000	-1.258081000
6	-2.601072000	0.591377000	-1.138360000
1	-2.479902000	0.520994000	-0.050035000

6	-1.386825000	1.310027000	-1.718926000
1	-0.470178000	0.733042000	-1.572204000
1	-1.510787000	1.448381000	-2.794862000
1	-1.248750000	2.300735000	-1.278142000
6	-3.878590000	1.376781000	-1.414490000
1	-3.822447000	2.387423000	-1.007222000
1	-4.043668000	1.462506000	-2.490079000
1	-4.748841000	0.883991000	-0.981428000
b			
29	-0.557979000	-0.744679000	-0.633683000
6	-2.420314000	-1.316147000	-1.043235000
6	2.683170000	-3.100580000	1.155704000
6	1.961874000	0.715155000	-0.523682000
6	2.891143000	1.746518000	-0.572532000
6	2.536763000	2.949164000	-1.162244000
6	1.265256000	3.096692000	-1.688472000
6	0.390387000	2.028935000	-1.587130000
7	0.726958000	0.872699000	-1.020149000
1	3.877771000	1.625064000	-0.154744000
1	3.249317000	3.760805000	-1.207384000
1	0.949393000	4.015549000	-2.158919000
1	-0.620130000	2.099799000	-1.967945000
6	2.268512000	-0.616491000	0.075190000
6	3.533927000	-0.955805000	0.534646000
6	3.741173000	-2.211987000	1.081286000
6	1.452292000	-2.691490000	0.671348000
7	1.249547000	-1.485577000	0.145654000
1	4.354808000	-0.260151000	0.464661000
1	4.721216000	-2.490652000	1.442412000
1	2.800947000	-4.088510000	1.574583000
1	0.594756000	-3.350074000	0.705380000
6	-1.513144000	1.704882000	1.495986000
1	-1.659010000	1.471049000	0.432535000
6	-0.528690000	2.865474000	1.598358000
1	-0.390456000	3.146229000	2.644453000
1	0.451133000	2.597995000	1.196316000
1	-0.890809000	3.744313000	1.063625000
17	-3.760715000	-0.948613000	-0.025206000
6	-2.866735000	2.091811000	2.081918000
1	-3.577759000	1.267052000	2.027199000
1	-2.755784000	2.363159000	3.133498000
1	-3.295410000	2.947354000	1.560092000
6	-0.952515000	0.469550000	2.194692000
1	-0.879282000	0.645953000	3.269847000
1	-1.592966000	-0.405168000	2.052218000
1	0.051950000	0.228791000	1.838686000
8	-2.757308000	-1.973494000	-2.087589000
1	-3.711795000	-2.172465000	-2.160009000
c			
29	-0.579751000	0.145194000	-0.971623000
6	-2.536293000	-0.023468000	-1.251619000
6	2.400686000	-3.069453000	-0.999859000
6	2.085127000	1.071869000	-0.194524000
6	3.133696000	1.878863000	0.227165000
6	2.903176000	3.226955000	0.447661000
6	1.633670000	3.739037000	0.243849000
6	0.640677000	2.871357000	-0.177795000
7	0.859342000	1.576244000	-0.393169000
1	4.121058000	1.472970000	0.378871000
1	3.709944000	3.868173000	0.774099000
1	1.411523000	4.783296000	0.403787000
1	-0.368578000	3.221325000	-0.349779000
6	2.248301000	-0.387279000	-0.457689000
6	3.443854000	-1.059051000	-0.237128000
6	3.517510000	-2.415365000	-0.508588000
6	1.244437000	-2.332012000	-1.188766000
7	1.167854000	-1.030351000	-0.920053000
1	4.309146000	-0.542276000	0.146152000
1	4.440061000	-2.952592000	-0.338759000
1	2.416678000	-4.124025000	-1.230147000
1	0.343650000	-2.797205000	-1.567603000
6	-0.664831000	-0.967163000	2.157558000
1	-0.495753000	-0.109855000	1.492548000
6	0.687840000	-1.586419000	2.496949000
1	0.558555000	-2.394341000	3.219910000
1	1.172632000	-2.009156000	1.615137000
1	1.359853000	-0.849942000	2.939474000
6	-1.346565000	-0.446129000	3.418089000
1	-2.307826000	0.014821000	3.188733000
1	-1.529937000	-1.266811000	4.114288000
1	-0.727370000	0.292177000	3.927396000
6	-1.554818000	-1.978063000	1.441129000
1	-1.719368000	-2.853441000	2.072676000
1	-2.539240000	-1.559056000	1.215869000
1	-1.102682000	-2.325222000	0.509241000
6	-3.604469000	0.499574000	-0.257918000

8	-4.768094000	0.281330000	-0.446064000
8	-3.074274000	1.181900000	0.736667000
1	-3.774940000	1.492572000	1.332275000
8	-3.147229000	-0.585548000	-2.210505000
1	-4.130212000	-0.554238000	-2.066405000
d			
29	-0.567746000	-0.791933000	-0.689276000
6	-2.355683000	-1.479303000	-1.202682000
6	2.730358000	-2.978284000	1.194825000
6	1.850104000	0.805466000	-0.482209000
6	2.719452000	1.888480000	-0.492223000
6	2.309998000	3.080079000	-1.068719000
6	1.043946000	3.165878000	-1.620922000
6	0.230151000	2.048077000	-1.561322000
7	0.620514000	0.901338000	-1.008633000
1	3.701583000	1.816228000	-0.053117000
1	2.975551000	3.931799000	-1.081911000
1	0.685259000	4.075174000	-2.079053000
1	-0.774804000	2.070570000	-1.962198000
6	2.214011000	-0.513395000	0.111941000
6	3.475890000	-0.780327000	0.625142000
6	3.734767000	-2.026774000	1.172211000
6	1.499474000	-2.640456000	0.658998000
7	1.247881000	-1.444316000	0.130576000
1	4.254435000	-0.034795000	0.599742000
1	4.712657000	-2.248301000	1.576218000
1	2.888579000	-3.960068000	1.614835000
1	0.681943000	-3.348886000	0.655254000
6	-1.747806000	1.562316000	1.441921000
1	-1.858555000	1.347784000	0.368973000
6	-0.789922000	2.739338000	1.598278000
1	-0.699386000	3.005440000	2.653340000
1	0.211385000	2.499573000	1.232917000
1	-1.149543000	3.619870000	1.064600000
6	-3.125670000	1.911712000	1.994273000
1	-3.822937000	1.081167000	1.889606000
1	-3.049669000	2.153273000	3.056288000
1	-3.546093000	2.778980000	1.485117000
6	-1.183100000	0.323457000	2.131928000
1	-1.132387000	0.485373000	3.210618000
1	-1.811356000	-0.555940000	1.969053000
1	-0.166429000	0.103223000	1.795216000
6	-3.451492000	-1.328547000	-0.276763000
7	-4.218537000	-1.151506000	0.560918000
8	-2.759459000	-2.060763000	-2.277772000
1	-2.035800000	-2.173200000	-2.912414000
e			
29	-0.585614000	-0.767817000	-0.607840000
6	-2.437060000	-1.312435000	-0.991263000
6	2.664471000	-3.069889000	1.226038000
6	1.941345000	0.692807000	-0.565982000
6	2.873501000	1.717804000	-0.664913000
6	2.510392000	2.904964000	-1.280276000
6	1.227647000	3.043555000	-1.781065000
6	0.351686000	1.982139000	-1.632188000
7	0.697185000	0.840351000	-1.041815000
1	3.869105000	1.603396000	-0.266841000
1	3.224820000	3.711850000	-1.364331000
1	0.904234000	3.950996000	-2.268259000
1	-0.667048000	2.046741000	-1.991674000
6	2.251257000	-0.620574000	0.068791000
6	3.516555000	-0.946064000	0.538220000
6	3.723185000	-2.184887000	1.123313000
6	1.433530000	-2.675019000	0.730179000
7	1.231602000	-1.486257000	0.166510000
1	4.336996000	-0.252046000	0.449085000
1	4.702910000	-2.452532000	1.493514000
1	2.781506000	-4.043803000	1.676744000
1	0.574764000	-3.330399000	0.787479000
6	-1.462594000	1.761020000	1.507240000
1	-1.639942000	1.501713000	0.454883000
6	-0.469805000	2.918037000	1.554382000
1	-0.299660000	3.222404000	2.589116000
1	0.496688000	2.635456000	1.130793000
1	-0.842528000	3.786388000	1.009809000
17	-3.681604000	-0.873362000	0.052736000
6	-2.796852000	2.169556000	2.122331000
1	-3.512390000	1.346994000	2.109124000
1	-2.654194000	2.466888000	3.163035000
1	-3.236995000	3.013817000	1.591678000
6	-0.889085000	0.538806000	2.218123000
1	-0.787418000	0.737451000	3.287087000
1	-1.536511000	-0.335303000	2.108585000
1	0.104647000	0.286515000	1.840856000
6	-2.905991000	-2.110369000	-2.131942000
1	-3.967814000	-2.031573000	-2.358579000

1	-2.279052000	-1.938308000	-3.006115000
1	-2.696638000	-3.148165000	-1.823948000
f			
29	0.380535000	0.823072000	-0.616299000
6	2.195649000	1.379623000	-1.041956000
6	-3.017640000	2.961454000	1.122623000
6	-2.031574000	-0.796901000	-0.548743000
6	-2.887254000	-1.888570000	-0.615373000
6	-2.432020000	-3.069146000	-1.180661000
6	-1.136295000	-3.135547000	-1.662880000
6	-0.339317000	-2.009130000	-1.550597000
7	-0.776111000	-0.873506000	-1.010802000
1	-3.891853000	-1.831690000	-0.227386000
1	-3.085369000	-3.928436000	-1.238348000
1	-0.742738000	-4.036664000	-2.108385000
1	0.687008000	-2.012181000	-1.895146000
6	-2.434185000	0.512241000	0.040435000
6	-3.727426000	0.774618000	0.470603000
6	-4.020313000	2.013472000	1.017661000
6	-1.753675000	2.628585000	0.666183000
7	-1.469259000	1.439697000	0.138616000
1	-4.503462000	0.031563000	0.378106000
1	-5.023099000	2.232603000	1.356717000
1	-3.202407000	3.937673000	1.544748000
1	-0.936299000	3.334536000	0.725937000
6	1.413392000	-1.602126000	1.560362000
1	1.549580000	-1.403337000	0.490911000
6	0.449138000	-2.774366000	1.707551000
1	0.307861000	-3.015868000	2.763053000
1	-0.532361000	-2.539853000	1.288285000
1	0.829601000	-3.666858000	1.209607000
6	2.652907000	2.168669000	-2.184699000
1	3.463503000	2.853815000	-1.911355000
1	3.133498000	1.424110000	-2.844415000
1	1.865267000	2.671106000	-2.733793000
6	2.778839000	-1.946782000	2.144130000
1	3.469553000	-1.106434000	2.061768000
1	2.685356000	-2.198028000	3.202475000
1	3.227642000	-2.796385000	1.630710000
6	0.840043000	-0.353742000	2.225485000
1	0.774179000	-0.498107000	3.306030000
1	1.466905000	0.526310000	2.053913000
1	-0.169501000	-0.136720000	1.868200000
6	3.309363000	0.774549000	-0.293660000
8	3.709436000	-0.299162000	-0.654364000
8	3.750832000	1.486908000	0.732667000
1	4.469263000	1.003043000	1.171130000
g			
29	0.581508000	0.800071000	-0.680084000
6	2.352848000	1.468020000	-1.119711000
6	-2.680209000	2.990022000	1.231739000
6	-1.844055000	-0.772526000	-0.510150000
6	-2.728155000	-1.841847000	-0.561166000
6	-2.326140000	-3.024027000	-1.162009000
6	-1.053020000	-3.113928000	-1.697018000
6	-0.224634000	-2.009697000	-1.597922000
7	-0.608427000	-0.872442000	-1.022132000
1	-3.716755000	-1.765287000	-0.137465000
1	-3.003354000	-3.865334000	-1.207842000
1	-0.700209000	-4.016325000	-2.172952000
1	0.785382000	-2.034928000	-1.985219000
6	-2.194738000	0.537023000	0.109241000
6	-3.449983000	0.807130000	0.635667000
6	-3.693156000	2.047884000	1.202922000
6	-1.455390000	2.649202000	0.683702000
7	-1.219440000	1.458458000	0.136923000
1	-4.234617000	0.068068000	0.606420000
1	-4.665864000	2.272248000	1.617706000
1	-2.826914000	3.967221000	1.666315000
1	-0.631157000	3.349578000	0.683803000
6	1.688674000	-1.635535000	1.458858000
1	1.824081000	-1.393942000	0.395148000
6	0.728553000	-2.816356000	1.563653000
1	0.612829000	-3.109041000	2.609199000
1	-0.263970000	-2.567050000	1.180720000
1	1.100676000	-3.683381000	1.016473000
6	3.373431000	1.288982000	-0.164899000
7	4.155941000	1.127486000	0.669977000
6	2.791200000	2.103835000	-2.365806000
1	3.588058000	2.839001000	-2.241338000
1	3.258467000	1.262619000	-2.913754000
1	1.973733000	2.469789000	-2.977640000
6	3.054091000	-1.996493000	2.034373000
1	3.751394000	-1.162199000	1.963589000
1	2.955261000	-2.262083000	3.088774000
1	3.487400000	-2.851574000	1.515345000

6	1.106969000	-0.414089000	2.165051000
1	1.027849000	-0.603254000	3.237607000
1	1.739569000	0.467884000	2.038736000
1	0.100170000	-0.184198000	1.806288000
		h	
29	-0.587724000	-0.761938000	-0.636960000
6	-2.472569000	-1.319472000	-1.039915000
6	2.657617000	-3.099721000	1.189945000
6	1.957242000	0.690292000	-0.555012000
6	2.893638000	1.714399000	-0.624278000
6	2.541261000	2.913662000	-1.221892000
6	1.264655000	3.065170000	-1.734576000
6	0.384445000	2.003488000	-1.614769000
7	0.719492000	0.849935000	-1.041943000
1	3.883851000	1.590477000	-0.215763000
1	3.258706000	3.719984000	-1.282393000
1	0.949104000	3.982603000	-2.208068000
1	-0.630427000	2.077881000	-1.983658000
6	2.258156000	-0.634828000	0.061447000
6	3.520515000	-0.970913000	0.532517000
6	3.720133000	-2.217538000	1.103050000
6	1.430841000	-2.693979000	0.692204000
7	1.235590000	-1.497981000	0.142011000
1	4.344082000	-0.279115000	0.455947000
1	4.697124000	-2.493184000	1.474639000
1	2.768688000	-4.079204000	1.630022000
1	0.569106000	-3.346656000	0.737585000
6	-1.450434000	1.732785000	1.510786000
1	-1.624853000	1.490145000	0.453947000
6	-0.451018000	2.882909000	1.580763000
1	-0.285006000	3.170495000	2.621025000
1	0.516276000	2.601287000	1.158426000
1	-0.815576000	3.761728000	1.047471000
17	-3.727598000	-0.858607000	0.047733000
6	-2.784977000	2.140277000	2.125729000
1	-3.505450000	1.322782000	2.095156000
1	-2.645134000	2.420374000	3.171658000
1	-3.216863000	2.995609000	1.606057000
6	-0.888027000	0.496300000	2.205770000
1	-0.792717000	0.677253000	3.278538000
1	-1.539415000	-0.372270000	2.077434000
1	0.106884000	0.245253000	1.830944000
7	-2.938925000	-1.991869000	-2.047918000

1	-3.924749000	-2.210313000	-2.163510000
1	-2.302448000	-2.320180000	-2.760750000
		i	
29	0.627601000	0.509105000	-0.703470000
6	2.559764000	0.657501000	-1.209455000
6	-1.975411000	3.557891000	1.079185000
6	-2.190600000	-0.327699000	-0.580388000
6	-3.347650000	-1.093062000	-0.660791000
6	-3.285525000	-2.355334000	-1.228482000
6	-2.072358000	-2.827244000	-1.698820000
6	-0.962365000	-2.010014000	-1.570243000
7	-1.018609000	-0.795785000	-1.028978000
1	-4.288200000	-0.719808000	-0.288231000
1	-4.177525000	-2.961897000	-1.298996000
1	-1.979371000	-3.805370000	-2.146119000
1	0.012837000	-2.340048000	-1.904935000
6	-2.170988000	1.045044000	0.004674000
6	-3.314092000	1.670449000	0.484251000
6	-3.212848000	2.941019000	1.027519000
6	-0.882900000	2.871189000	0.576430000
7	-0.976639000	1.651482000	0.053070000
1	-4.274779000	1.182868000	0.439085000
1	-4.093772000	3.440165000	1.406187000
1	-1.851395000	4.545746000	1.496511000
1	0.106252000	3.309299000	0.594419000
6	0.753014000	-1.974142000	1.652930000
1	1.014036000	-1.731073000	0.614206000
6	-0.340026000	-3.037286000	1.634701000
1	-0.592360000	-3.330962000	2.655800000
1	-1.252429000	-2.662852000	1.165141000
1	-0.020812000	-3.933148000	1.100530000
6	2.009056000	-2.499462000	2.339375000
1	2.798761000	-1.747692000	2.348634000
1	1.789321000	-2.764462000	3.375362000
1	2.392581000	-3.390033000	1.841275000
6	0.247875000	-0.706251000	2.334359000
1	0.059264000	-0.898571000	3.392773000
1	0.973939000	0.105940000	2.264727000
1	-0.690728000	-0.367119000	1.892267000
6	3.690745000	0.637012000	-0.173202000
8	4.851856000	0.594433000	-0.462403000
8	3.210224000	0.679020000	1.063013000
1	3.952049000	0.671342000	1.687986000

7	3.099521000	0.785488000	-2.371931000
1	4.117604000	0.838661000	-2.463587000
1	2.543590000	0.821384000	-3.214684000
j			
29	-0.588304000	-0.795976000	-0.686459000
6	-2.421661000	-1.456990000	-1.168722000
6	2.702233000	-3.010590000	1.204509000
6	1.869695000	0.771863000	-0.498874000
6	2.753981000	1.843203000	-0.513974000
6	2.361453000	3.036496000	-1.098289000
6	1.097247000	3.135372000	-1.652551000
6	0.268852000	2.028487000	-1.586303000
7	0.642539000	0.880207000	-1.026439000
1	3.734021000	1.760818000	-0.071926000
1	3.038108000	3.879351000	-1.115482000
1	0.751190000	4.046607000	-2.116561000
1	-0.735599000	2.061380000	-1.988026000
6	2.216344000	-0.548205000	0.103841000
6	3.478840000	-0.831075000	0.608060000
6	3.722225000	-2.076344000	1.164341000
6	1.472634000	-2.656738000	0.675680000
7	1.235575000	-1.462007000	0.138516000
1	4.269549000	-0.099077000	0.567840000
1	4.699960000	-2.310470000	1.561565000
1	2.847908000	-3.990915000	1.632441000
1	0.643240000	-3.351360000	0.684849000
6	-1.696715000	1.616030000	1.439933000
1	-1.816797000	1.385878000	0.371606000
6	-0.713583000	2.774864000	1.573321000
1	-0.612154000	3.055767000	2.623637000
1	0.280367000	2.507801000	1.206969000
1	-1.056763000	3.654368000	1.027070000
6	-3.064180000	2.002876000	1.993249000
1	-3.779057000	1.185769000	1.901986000
1	-2.978571000	2.257194000	3.051633000
1	-3.467920000	2.872302000	1.474065000
6	-1.155077000	0.376262000	2.146138000
1	-1.088191000	0.556069000	3.221210000
1	-1.806688000	-0.489269000	2.003754000
1	-0.148986000	0.124468000	1.800343000
6	-3.469540000	-1.264251000	-0.205417000
7	-4.241237000	-1.077773000	0.626006000
7	-2.822623000	-2.041370000	-2.255584000

1	-3.782174000	-2.335305000	-2.415184000
1	-2.166911000	-2.229039000	-3.001615000
TS			
a			
29	0.645321000	-0.008097000	0.807232000
6	2.567721000	-0.026414000	1.439519000
1	3.552633000	-0.003565000	0.703362000
1	2.953043000	-0.926732000	1.930417000
6	-1.910453000	3.486352000	-0.054842000
6	-2.078964000	-0.739784000	-0.125322000
6	-3.192610000	-1.483546000	-0.495144000
6	-3.122957000	-2.866588000	-0.462276000
6	-1.946338000	-3.475426000	-0.061403000
6	-0.880630000	-2.664167000	0.290090000
7	-0.942758000	-1.335341000	0.258136000
1	-4.106087000	-1.001127000	-0.804511000
1	-3.981363000	-3.459360000	-0.745784000
1	-1.850453000	-4.549890000	-0.018192000
1	0.060683000	-3.090122000	0.613370000
6	-2.071363000	0.752270000	-0.123413000
6	-3.181357000	1.508147000	-0.479426000
6	-3.097381000	2.890317000	-0.444432000
6	-0.849753000	2.663567000	0.284688000
7	-0.925547000	1.335458000	0.250572000
1	-4.103532000	1.035625000	-0.778060000
1	-3.952865000	3.492368000	-0.717011000
1	-1.803177000	4.559692000	-0.010497000
1	0.098816000	3.079361000	0.599908000
1	2.953998000	0.850687000	1.970816000
6	3.619026000	-0.002362000	-0.534306000
6	3.041455000	1.273860000	-1.086175000
6	3.044518000	-1.273889000	-1.099671000
6	5.142510000	-0.001331000	-0.492330000
1	3.429340000	1.399668000	-2.099557000
1	1.954861000	1.239509000	-1.135936000
1	3.351557000	2.138400000	-0.501473000
1	1.957758000	-1.242640000	-1.147407000
1	3.431339000	-1.386614000	-2.114995000
1	3.358323000	-2.144127000	-0.525551000
1	5.497162000	0.002871000	-1.524950000
1	5.530128000	0.885943000	0.004456000
1	5.531116000	-0.891989000	-0.002414000
b			

29	-0.136427000	0.125113000	-0.078793000
6	-2.135161000	-0.498182000	-0.250113000
6	2.422626000	-3.466900000	0.205392000
6	2.699559000	0.752120000	0.043329000
6	3.891544000	1.465156000	0.048786000
6	3.857597000	2.848094000	-0.012294000
6	2.634335000	3.491850000	-0.078820000
6	1.489558000	2.714787000	-0.081716000
7	1.517101000	1.384048000	-0.021809000
1	4.840386000	0.955475000	0.096227000
1	4.778829000	3.413696000	-0.009681000
1	2.560893000	4.567641000	-0.129402000
1	0.510425000	3.171367000	-0.136187000
6	2.660733000	-0.739472000	0.104552000
6	3.806614000	-1.516786000	0.212653000
6	3.682014000	-2.896236000	0.262290000
6	1.328096000	-2.624021000	0.100889000
7	1.447331000	-1.300663000	0.051332000
1	4.785384000	-1.066834000	0.260553000
1	4.563888000	-3.515886000	0.345775000
1	2.284355000	-4.536985000	0.241697000
1	0.318131000	-3.010927000	0.057563000
1	-2.304941000	0.394057000	0.402612000
6	-3.756672000	0.493253000	0.714717000
6	-3.881601000	1.856288000	0.088494000
1	-4.149194000	1.793824000	-0.964088000
1	-4.668763000	2.408149000	0.607886000
1	-2.960310000	2.435365000	0.190090000
6	-3.366543000	0.483869000	2.172905000
1	-4.229938000	0.778749000	2.773002000
1	-3.063510000	-0.514919000	2.487315000
1	-2.560331000	1.188306000	2.385949000
6	-4.852423000	-0.464768000	0.368262000
1	-4.735031000	-1.424752000	0.870883000
1	-5.791426000	-0.038150000	0.731525000
1	-4.958888000	-0.603017000	-0.707758000
17	-2.587278000	-0.342927000	-1.933455000
8	-2.234713000	-1.747498000	0.263198000
1	-3.034817000	-2.191685000	-0.058444000
	c		
29	-0.028259000	0.017583000	-0.156643000
6	-2.033803000	-0.499420000	-0.366203000
6	2.700739000	-3.452452000	0.044062000

6	2.781827000	0.777363000	0.078393000
6	3.935400000	1.547840000	0.150442000
6	3.830885000	2.928828000	0.140360000
6	2.578597000	3.512323000	0.056470000
6	1.475728000	2.679181000	-0.013922000
7	1.572064000	1.350886000	-0.001580000
1	4.907509000	1.084964000	0.208787000
1	4.720970000	3.539924000	0.194653000
1	2.452292000	4.584350000	0.042765000
1	0.474768000	3.084098000	-0.086484000
6	2.812339000	-0.715513000	0.076971000
6	3.989156000	-1.443542000	0.194659000
6	3.928633000	-2.828093000	0.177245000
6	1.571856000	-2.656484000	-0.064168000
7	1.629143000	-1.327987000	-0.047122000
1	4.942682000	-0.951070000	0.300634000
1	4.835183000	-3.410136000	0.267121000
1	2.612168000	-4.528241000	0.024245000
1	0.584511000	-3.088005000	-0.168982000
1	-2.210423000	0.041398000	0.633129000
6	-3.534948000	0.140271000	1.230374000
6	-3.327545000	1.587676000	1.577576000
1	-3.460993000	2.233392000	0.713582000
1	-4.078041000	1.857772000	2.326431000
1	-2.346076000	1.762310000	2.020512000
6	-3.193181000	-0.851226000	2.304360000
1	-3.987639000	-0.819173000	3.054420000
1	-3.149175000	-1.862566000	1.904043000
1	-2.252581000	-0.609937000	2.798994000
6	-4.767035000	-0.182219000	0.449258000
1	-4.723980000	-1.182932000	0.018148000
1	-5.607935000	-0.174590000	1.149350000
1	-4.975390000	0.550050000	-0.328195000
6	-2.696675000	0.151207000	-1.515796000
8	-3.225951000	-0.487474000	-2.389030000
8	-2.629728000	1.491324000	-1.507019000
1	-3.068557000	1.812184000	-2.308524000
8	-2.232051000	-1.842281000	-0.347664000
1	-2.868220000	-2.060985000	-1.051849000
	d		
29	0.167886000	-0.240298000	0.347230000
6	2.210514000	0.029190000	0.725736000
6	-2.814239000	-3.408182000	0.037469000

6	-2.530622000	0.814598000	-0.132837000
6	-3.603055000	1.665482000	-0.364789000
6	-3.385146000	3.034131000	-0.388455000
6	-2.106694000	3.520265000	-0.181770000
6	-1.086965000	2.608128000	0.039131000
7	-1.295294000	1.293833000	0.061374000
1	-4.596738000	1.280118000	-0.528287000
1	-4.209716000	3.710025000	-0.567213000
1	-1.894394000	4.578726000	-0.190346000
1	-0.068106000	2.936513000	0.203802000
6	-2.679140000	-0.670548000	-0.083677000
6	-3.910427000	-1.300595000	-0.214632000
6	-3.977036000	-2.682374000	-0.154189000
6	-1.625573000	-2.711080000	0.161733000
7	-1.556935000	-1.381899000	0.101970000
1	-4.812722000	-0.727872000	-0.356663000
1	-4.929635000	-3.183779000	-0.253193000
1	-2.820784000	-4.486194000	0.093580000
1	-0.690434000	-3.231833000	0.318603000
1	2.471853000	-0.542200000	-0.230933000
6	3.815084000	-0.547627000	-0.771126000
6	3.464519000	0.187090000	-2.030142000
1	3.439962000	1.264553000	-1.875579000
1	4.241423000	-0.028186000	-2.769489000
1	2.514909000	-0.150148000	-2.447399000
6	3.739555000	-2.050405000	-0.814272000
1	4.639765000	-2.420506000	-1.311571000
1	3.721534000	-2.466328000	0.193477000
1	2.874775000	-2.402503000	-1.375715000
6	4.961334000	-0.003200000	0.015198000
1	5.067280000	-0.503675000	0.977490000
1	5.871082000	-0.225203000	-0.551211000
1	4.908178000	1.077259000	0.140467000
8	2.648511000	-0.561097000	1.874562000
1	3.362474000	-0.056961000	2.287823000
6	2.405244000	1.442094000	0.594376000
7	2.507713000	2.584324000	0.486609000

e

29	-0.157240000	-0.120534000	-0.242163000
6	-2.127292000	0.485693000	-0.478113000
6	2.515599000	-3.560049000	-0.006488000
6	2.672470000	0.670672000	0.060818000
6	3.834711000	1.412968000	0.226524000
6	3.755276000	2.796517000	0.230726000
6	2.522735000	3.404995000	0.072295000
6	1.409632000	2.594514000	-0.082545000
7	1.484149000	1.267064000	-0.088618000
1	4.791350000	0.932425000	0.355845000
1	4.650037000	3.389494000	0.358845000
1	2.417856000	4.479508000	0.070569000
1	0.420036000	3.017017000	-0.201703000
6	2.665870000	-0.821823000	0.041805000
6	3.832376000	-1.572088000	0.124114000
6	3.755431000	-2.954261000	0.100532000
6	1.399504000	-2.746494000	-0.087594000
7	1.467750000	-1.416179000	-0.064072000
1	4.794411000	-1.091483000	0.200907000
1	4.656143000	-3.548733000	0.163199000
1	2.408599000	-4.633996000	-0.029550000
1	0.409286000	-3.172918000	-0.176766000
1	-2.411767000	-0.442523000	0.108019000
6	-3.777296000	-0.800965000	0.366396000
6	-3.522567000	-0.808892000	1.850149000
1	-3.585641000	0.191536000	2.272413000
1	-4.292124000	-1.425702000	2.320900000
1	-2.552838000	-1.246305000	2.095316000
6	-3.605446000	-2.131076000	-0.316290000
1	-4.388495000	-2.802229000	0.045941000
1	-3.711458000	-2.051740000	-1.396180000
1	-2.644897000	-2.590372000	-0.075527000
6	-4.956227000	0.004760000	-0.066061000
1	-5.146364000	-0.073531000	-1.133424000
1	-5.830074000	-0.396940000	0.455809000
1	-4.861951000	1.051267000	0.225183000
17	-2.345420000	1.941103000	0.491386000
6	-2.591682000	0.660682000	-1.891147000
1	-2.631647000	-0.299216000	-2.405118000
1	-3.557009000	1.164845000	-1.970190000
1	-1.863214000	1.279218000	-2.417203000

f

29	0.226259000	0.017269000	-0.175449000
6	2.189925000	0.634135000	-0.511435000
1	3.241481000	0.201519000	-0.221000000
6	-2.168926000	-3.661952000	0.088816000
6	-2.661316000	0.545117000	0.035671000
6	-3.888652000	1.191131000	0.097664000

6	-3.922286000	2.576649000	0.064162000
6	-2.735721000	3.280754000	-0.029988000
6	-1.549194000	2.565649000	-0.087389000
7	-1.518050000	1.235033000	-0.054514000
1	-4.810624000	0.636623000	0.169227000
1	-4.869472000	3.095647000	0.111213000
1	-2.719054000	4.359875000	-0.059183000
1	-0.588944000	3.061088000	-0.156613000
6	-2.533170000	-0.942212000	0.061793000
6	-3.637004000	-1.780610000	0.166838000
6	-3.452739000	-3.152230000	0.180360000
6	-1.121166000	-2.764554000	-0.010792000
7	-1.291486000	-1.443497000	-0.023536000
1	-4.633480000	-1.375430000	0.238051000
1	-4.304225000	-3.813322000	0.261142000
1	-1.977848000	-4.724404000	0.094031000
1	-0.100139000	-3.117781000	-0.083807000
6	3.272310000	-1.086220000	0.110147000
6	2.971818000	-1.957800000	-1.075798000
6	2.557334000	-1.453409000	1.381111000
6	4.767960000	-0.862144000	0.324117000
1	3.365504000	-2.950478000	-0.844001000
1	1.901631000	-2.039269000	-1.257584000
1	3.467279000	-1.608532000	-1.979084000
1	1.512079000	-1.699247000	1.204290000
1	3.058690000	-2.340792000	1.777443000
1	2.629287000	-0.666241000	2.125657000
1	5.200009000	-1.830073000	0.585064000
1	5.258351000	-0.506059000	-0.580688000
1	4.949648000	-0.168100000	1.141743000
6	2.240028000	1.811869000	0.372060000
8	1.692145000	2.858556000	0.127006000
8	2.919834000	1.615270000	1.525186000
1	2.851991000	2.433024000	2.038495000
6	2.310199000	0.916421000	-2.002132000
1	3.319793000	1.181071000	-2.323099000
1	1.948517000	0.095929000	-2.617410000
1	1.672537000	1.779202000	-2.198273000
		g	
29	0.278639000	0.325915000	-0.654839000
6	2.287653000	0.139343000	-1.047819000
6	-1.659902000	-3.607882000	0.191965000
6	-2.556027000	0.538844000	0.076964000

6	-3.817203000	1.084286000	0.326260000
6	-3.994987000	2.461396000	0.240951000
6	-2.910418000	3.265576000	-0.092906000
6	-1.687121000	2.647987000	-0.328377000
7	-1.509141000	1.323843000	-0.246005000
1	-4.661068000	0.446344000	0.578722000
1	-4.975191000	2.898988000	0.431053000
1	-3.003265000	4.347303000	-0.174605000
1	-0.807623000	3.234992000	-0.598495000
6	-2.292502000	-0.933380000	0.141906000
6	-3.270973000	-1.856881000	0.513493000
6	-2.946710000	-3.211087000	0.537498000
6	-0.739639000	-2.623743000	-0.162126000
7	-1.048225000	-1.323367000	-0.184417000
1	-4.275074000	-1.538152000	0.784543000
1	-3.698324000	-3.947114000	0.823628000
1	-1.365629000	-4.656254000	0.193026000
1	0.284600000	-2.882093000	-0.442794000
1	2.729848000	0.350022000	0.108218000
6	3.175902000	0.562358000	1.275938000
6	4.635279000	0.228837000	1.148482000
1	5.155980000	0.919811000	0.472179000
1	5.094543000	0.327624000	2.147085000
1	4.781498000	-0.808678000	0.815153000
6	2.338649000	-0.425703000	2.042778000
1	2.630667000	-0.366905000	3.104683000
1	1.266359000	-0.190359000	1.969650000
1	2.518869000	-1.454812000	1.700289000
6	2.817488000	2.012692000	1.448616000
1	3.073485000	2.303308000	2.481495000
1	3.385303000	2.659362000	0.766915000
1	1.738539000	2.174993000	1.309881000
6	3.138977000	0.996893000	-1.950487000
1	2.790302000	0.797614000	-2.978649000
1	2.974903000	2.064075000	-1.756844000
1	4.213704000	0.758752000	-1.922714000
6	2.595502000	-1.269493000	-1.092642000
7	2.754973000	-2.421989000	-1.061729000
		h	
29	-0.163315000	-0.104126000	-0.273097000
6	-2.182095000	0.432125000	-0.519087000
6	2.498106000	-3.553579000	-0.002103000
6	2.653544000	0.677369000	0.062723000

6	3.811253000	1.419416000	0.257191000
6	3.732576000	2.803022000	0.259270000
6	2.504659000	3.412248000	0.070687000
6	1.394977000	2.602801000	-0.110515000
7	1.468985000	1.274837000	-0.115108000
1	4.763632000	0.938044000	0.412252000
1	4.624151000	3.395494000	0.409937000
1	2.400367000	4.486795000	0.066690000
1	0.408631000	3.025504000	-0.252335000
6	2.648229000	-0.815299000	0.044647000
6	3.814426000	-1.564999000	0.133030000
6	3.737233000	-2.947403000	0.110489000
6	1.382000000	-2.740598000	-0.090409000
7	1.451128000	-1.410323000	-0.067475000
1	4.776285000	-1.084352000	0.212137000
1	4.637671000	-3.541796000	0.177727000
1	2.391518000	-4.627577000	-0.024633000
1	0.392149000	-3.166705000	-0.185648000
1	-2.282148000	-0.488939000	0.117431000
6	-3.697983000	-0.767581000	0.402733000
6	-3.450332000	-0.752376000	1.889162000
1	-3.552176000	0.247678000	2.304187000
1	-4.193470000	-1.396011000	2.365974000
1	-2.464726000	-1.149251000	2.143721000
6	-3.532870000	-2.106617000	-0.266813000
1	-4.413369000	-2.716511000	-0.054997000
1	-3.456856000	-1.985428000	-1.347230000
1	-2.658913000	-2.644135000	0.105395000
6	-4.906831000	-0.004802000	-0.039651000
1	-5.050674000	-0.062386000	-1.116791000
1	-5.776672000	-0.474187000	0.428459000
1	-4.882022000	1.033171000	0.289826000
17	-2.352347000	1.929849000	0.426435000
7	-2.640904000	0.454601000	-1.824720000
1	-1.927344000	0.532895000	-2.532174000
1	-3.405805000	1.093739000	-2.006497000
	i		
29	-0.053783000	0.307319000	0.271480000
6	-2.055543000	-0.298892000	0.513153000
6	2.319640000	-3.447223000	0.088417000
6	2.801954000	0.755206000	-0.033629000
6	4.021394000	1.402307000	-0.190349000
6	4.060188000	2.786109000	-0.215529000

6	2.880617000	3.497938000	-0.084166000
6	1.704509000	2.785134000	0.065933000
7	1.662153000	1.453457000	0.090393000
1	4.935661000	0.840639000	-0.294659000
1	5.003578000	3.299824000	-0.336955000
1	2.864180000	4.577223000	-0.097510000
1	0.756865000	3.295894000	0.171423000
6	2.687896000	-0.733891000	0.004131000
6	3.792830000	-1.571428000	-0.077934000
6	3.602392000	-2.943680000	-0.035045000
6	1.268251000	-2.547584000	0.167022000
7	1.451122000	-1.230274000	0.125426000
1	4.790913000	-1.174172000	-0.171045000
1	4.451880000	-3.609433000	-0.097074000
1	2.129375000	-4.509319000	0.125058000
1	0.243504000	-2.887205000	0.261975000
1	-2.386091000	0.735524000	0.169761000
6	-3.862956000	0.921271000	-0.104474000
6	-3.578543000	1.714288000	-1.348796000
1	-3.349691000	1.076490000	-2.196826000
1	-4.480163000	2.288596000	-1.582924000
1	-2.769171000	2.430731000	-1.199508000
6	-4.063330000	1.720673000	1.144672000
1	-5.074157000	2.135532000	1.122954000
1	-3.988290000	1.082502000	2.024810000
1	-3.359008000	2.548961000	1.220934000
6	-4.790207000	-0.241645000	-0.253718000
1	-4.769787000	-0.880703000	0.629817000
1	-5.806806000	0.154200000	-0.341352000
1	-4.585156000	-0.826256000	-1.148490000
6	-2.102033000	-1.330160000	-0.529748000
8	-2.182448000	-2.512362000	-0.296959000
8	-1.988668000	-0.850190000	-1.786224000
1	-1.998460000	-1.610260000	-2.385170000
7	-2.431791000	-0.737761000	1.771414000
1	-1.846884000	-0.483452000	2.549037000
1	-2.726376000	-1.705643000	1.798014000
	j		
29	0.191531000	-0.210551000	0.373406000
6	2.262027000	0.032878000	0.759875000
6	-2.738945000	-3.433994000	0.008401000
6	-2.520089000	0.795096000	-0.116299000
6	-3.609508000	1.630683000	-0.320982000

6	-3.411953000	3.002641000	-0.345579000
6	-2.136424000	3.506230000	-0.167133000
6	-1.098431000	2.609285000	0.032040000
7	-1.288105000	1.291828000	0.056767000
1	-4.601513000	1.231985000	-0.460504000
1	-4.250110000	3.666876000	-0.503707000
1	-1.938965000	4.567515000	-0.180099000
1	-0.080512000	2.950521000	0.174030000
6	-2.645696000	-0.692540000	-0.078273000
6	-3.863136000	-1.341463000	-0.245255000
6	-3.908926000	-2.724468000	-0.201088000
6	-1.565389000	-2.719039000	0.166587000
7	-1.516652000	-1.388048000	0.125366000
1	-4.770269000	-0.782147000	-0.407960000
1	-4.850566000	-3.239824000	-0.329063000
1	-2.728877000	-4.512644000	0.050084000
1	-0.625155000	-3.227568000	0.333854000
1	2.395799000	-0.579370000	-0.185867000
6	3.756113000	-0.529466000	-0.790167000
6	3.250379000	0.050693000	-2.078515000
1	3.117634000	1.129400000	-2.007000000
1	3.998377000	-0.141633000	-2.852698000
1	2.318848000	-0.416842000	-2.401471000
6	3.801902000	-2.026721000	-0.681726000
1	4.711567000	-2.382626000	-1.170698000
1	3.854710000	-2.324718000	0.367055000
1	2.948235000	-2.503467000	-1.163480000
6	4.919921000	0.169169000	-0.166951000
1	5.109899000	-0.200898000	0.840180000
1	5.801672000	-0.071329000	-0.768800000
1	4.803157000	1.251477000	-0.164065000
6	2.340360000	1.438952000	0.527594000
7	2.376487000	2.576079000	0.342669000
7	2.805667000	-0.480657000	1.933304000
1	2.205353000	-1.049153000	2.505906000
1	3.391296000	0.138387000	2.473600000
P			
a			
29	-0.622314000	0.082285000	-0.422219000
6	-2.886551000	0.119350000	-1.102687000
1	-3.531680000	0.179860000	-1.978576000
1	-2.292154000	-0.799945000	-1.257699000
6	2.169574000	3.436219000	0.044844000
6	2.095274000	-0.802426000	0.045653000
6	3.215249000	-1.597778000	0.253150000
6	3.082361000	-2.976568000	0.238280000
6	1.835600000	-3.534826000	0.017719000
6	0.766360000	-2.678776000	-0.179204000
7	0.890792000	-1.353144000	-0.165746000
1	4.184391000	-1.159349000	0.427647000
1	3.947005000	-3.605234000	0.398705000
1	1.687510000	-4.603949000	-0.002226000
1	-0.229027000	-3.066222000	-0.354684000
6	2.166517000	0.691392000	0.049827000
6	3.362421000	1.374297000	0.231869000
6	3.362284000	2.759497000	0.229119000
6	1.018912000	2.688448000	-0.132855000
7	1.015613000	1.356640000	-0.131031000
1	4.289893000	0.843410000	0.372661000
1	4.287304000	3.301059000	0.369168000
1	2.123955000	4.514731000	0.036018000
1	0.062071000	3.171260000	-0.282541000
1	-2.276414000	1.041786000	-1.146620000
6	-3.672807000	0.048147000	0.208265000
6	-4.535378000	1.301806000	0.344505000
6	-2.690669000	-0.029645000	1.378836000
6	-4.556326000	-1.198203000	0.195811000
1	-5.107525000	1.273196000	1.271868000
1	-3.921567000	2.204772000	0.355996000
1	-5.240359000	1.381115000	-0.483598000
1	-2.047325000	0.858526000	1.420784000
1	-3.215811000	-0.077504000	2.332728000
1	-2.066711000	-0.929838000	1.318715000
1	-5.128332000	-1.270901000	1.120855000
1	-5.262181000	-1.166866000	-0.634738000
1	-3.957693000	-2.106246000	0.098549000
b			
29	-0.137418000	-0.342787000	0.368091000
6	-2.930698000	-0.996470000	-0.452619000
1	-3.606910000	-1.831816000	-0.622470000
6	1.574478000	3.695276000	0.208859000
6	2.740974000	-0.364872000	-0.115020000
6	4.054732000	-0.781463000	-0.293650000
6	4.333262000	-2.136653000	-0.360721000
6	3.298020000	-3.047722000	-0.244136000
6	2.016917000	-2.555613000	-0.063363000

7	1.745853000	-1.254271000	-0.003833000
1	4.859715000	-0.068677000	-0.371545000
1	5.351143000	-2.473892000	-0.497746000
1	3.471949000	-4.112371000	-0.286073000
1	1.173442000	-3.225712000	0.043685000
6	2.366826000	1.079432000	-0.021729000
6	3.298907000	2.094638000	-0.197195000
6	2.897840000	3.415082000	-0.080760000
6	0.701849000	2.631685000	0.360399000
7	1.082431000	1.360862000	0.245600000
1	4.327816000	1.869324000	-0.426553000
1	3.614616000	4.213072000	-0.214918000
1	1.218366000	4.708965000	0.313780000
1	-0.343390000	2.800431000	0.582570000
6	-3.678072000	0.323605000	-0.319939000
6	-4.364499000	0.551576000	-1.675127000
6	-2.713851000	1.472775000	-0.029604000
6	-4.737325000	0.215349000	0.774908000
1	-4.958720000	1.462936000	-1.624751000
1	-3.636807000	0.656849000	-2.477391000
1	-5.038683000	-0.271105000	-1.919034000
1	-1.932730000	1.534014000	-0.790393000
1	-3.263066000	2.414114000	-0.036959000
1	-2.256940000	1.364515000	0.957000000
1	-5.348237000	1.117177000	0.774731000
1	-5.399947000	-0.634256000	0.601185000
1	-4.291231000	0.109041000	1.762289000
17	-2.104271000	-1.466451000	1.166507000
8	-1.926264000	-0.913202000	-1.393749000
1	-1.726699000	-1.782390000	-1.756867000
		c	
29	0.087222000	-0.183362000	0.871283000
6	-3.026875000	0.638599000	-0.034935000
1	-2.180803000	1.341400000	-0.067755000
6	2.136515000	3.617057000	0.052802000
6	2.803914000	-0.561628000	-0.132505000
6	3.970236000	-1.145908000	-0.610159000
6	4.081582000	-2.526686000	-0.618981000
6	3.028247000	-3.293649000	-0.152990000
6	1.896380000	-2.638364000	0.301128000
7	1.786100000	-1.312018000	0.310111000
1	4.785009000	-0.542943000	-0.977560000
1	4.983441000	-2.994881000	-0.987758000

1	3.074225000	-4.372161000	-0.140098000
1	1.044487000	-3.192449000	0.673043000
6	2.612370000	0.919165000	-0.084561000
6	3.619975000	1.809464000	-0.434817000
6	3.377924000	3.171242000	-0.366056000
6	1.185385000	2.670033000	0.389578000
7	1.413007000	1.359500000	0.322765000
1	4.587710000	1.455358000	-0.751653000
1	4.154174000	3.873944000	-0.634768000
1	1.905209000	4.669194000	0.122997000
1	0.202425000	2.969592000	0.728612000
6	-2.961014000	-0.245439000	-1.312030000
6	-3.217622000	0.679224000	-2.504679000
6	-4.034525000	-1.331850000	-1.247756000
6	-1.581073000	-0.882909000	-1.470402000
1	-3.144484000	0.105053000	-3.428086000
1	-4.206724000	1.126365000	-2.451393000
1	-2.477684000	1.481207000	-2.544657000
1	-5.015640000	-0.897137000	-1.065606000
1	-4.066465000	-1.871453000	-2.193910000
1	-3.826367000	-2.059790000	-0.460447000
1	-1.541259000	-1.414637000	-2.421014000
1	-0.788675000	-0.126776000	-1.494316000
1	-1.366658000	-1.613845000	-0.687781000
6	-2.823488000	-0.186368000	1.219587000
8	-1.731686000	-0.556015000	1.624671000
8	-3.927827000	-0.474912000	1.870361000
1	-3.710670000	-1.004094000	2.653867000
8	-4.255170000	1.302153000	-0.036824000
1	-4.424557000	1.731480000	0.805943000
		d	
29	0.366167000	-0.096189000	-0.296656000
6	-4.190762000	-0.056382000	-0.844065000
1	-4.464259000	-0.895726000	-1.491190000
6	2.802461000	3.528075000	0.194847000
6	3.200278000	-0.683635000	0.065924000
6	4.403466000	-1.365557000	0.194417000
6	4.407626000	-2.749437000	0.136241000
6	3.212794000	-3.422505000	-0.048884000
6	2.055036000	-2.673453000	-0.169688000
7	2.047376000	-1.343130000	-0.114347000
1	5.330703000	-0.834312000	0.337104000
1	5.336902000	-3.292937000	0.234640000

1	3.170816000	-4.499992000	-0.099945000
1	1.096122000	-3.152284000	-0.316244000
6	3.112999000	0.805968000	0.118814000
6	4.226342000	1.612938000	0.314451000
6	4.066912000	2.988498000	0.352405000
6	1.741972000	2.658644000	0.004818000
7	1.891943000	1.336503000	-0.032245000
1	5.208473000	1.185697000	0.438013000
1	4.924364000	3.629024000	0.504429000
1	2.634006000	4.594126000	0.218038000
1	0.733843000	3.030128000	-0.122568000
6	-4.892654000	-0.237713000	0.518160000
6	-6.397442000	-0.249305000	0.249629000
6	-4.527609000	0.919205000	1.446329000
6	-4.459961000	-1.571950000	1.123879000
1	-6.931679000	-0.381142000	1.189948000
1	-6.723010000	0.685339000	-0.202325000
1	-6.673782000	-1.073074000	-0.411154000
1	-4.812286000	1.875339000	1.010532000
1	-5.052613000	0.803980000	2.394025000
1	-3.456836000	0.937590000	1.660156000
1	-5.002314000	-1.740158000	2.053476000
1	-4.681938000	-2.405459000	0.454662000
1	-3.394068000	-1.587451000	1.357999000
6	-2.712668000	-0.117502000	-0.675475000
7	-1.576647000	-0.135140000	-0.548393000
8	-4.529794000	1.180014000	-1.408325000
1	-4.454379000	1.147693000	-2.365873000
e			
29	-0.044498000	-0.490909000	0.219245000
6	-3.558095000	-0.963170000	0.094156000
1	-4.195971000	-1.191534000	0.945843000
6	1.340559000	3.667761000	0.025283000
6	2.847067000	-0.291263000	-0.083530000
6	4.192595000	-0.611004000	-0.212364000
6	4.572937000	-1.943051000	-0.235994000
6	3.605059000	-2.926299000	-0.131226000
6	2.284652000	-2.529427000	-0.005875000
7	1.917314000	-1.250536000	0.016900000
1	4.943282000	0.158437000	-0.293646000
1	5.616779000	-2.205969000	-0.335315000
1	3.858663000	-3.975530000	-0.145004000
1	1.489478000	-3.258514000	0.079334000

6	2.359081000	1.120971000	-0.049918000
6	3.222036000	2.205594000	-0.143935000
6	2.706685000	3.490475000	-0.105504000
6	0.544032000	2.539608000	0.112659000
7	1.036030000	1.302871000	0.076609000
1	4.285460000	2.061295000	-0.245527000
1	3.368871000	4.341939000	-0.176845000
1	0.896611000	4.651145000	0.060218000
1	-0.530176000	2.623183000	0.216272000
6	-3.451443000	0.561129000	-0.049661000
6	-4.888522000	1.076609000	-0.240587000
6	-2.616285000	0.980230000	-1.258635000
6	-2.897548000	1.188105000	1.231855000
1	-4.877439000	2.165898000	-0.265613000
1	-5.327733000	0.730872000	-1.174300000
1	-5.533007000	0.768263000	0.583684000
1	-3.064733000	0.650808000	-2.194907000
1	-2.547391000	2.068010000	-1.295553000
1	-1.595809000	0.587089000	-1.224194000
1	-2.986430000	2.273498000	1.177746000
1	-3.454512000	0.851910000	2.107835000
1	-1.846872000	0.943437000	1.397569000
17	-1.956432000	-1.735376000	0.657163000
6	-3.983532000	-1.742465000	-1.129718000
1	-4.958892000	-1.391491000	-1.463999000
1	-3.270450000	-1.623143000	-1.943116000
1	-4.069017000	-2.801010000	-0.896948000
f			
29	-0.019426000	0.052944000	0.873475000
6	3.186562000	0.808596000	-0.143988000
1	4.203924000	1.199621000	-0.111733000
6	-2.642961000	3.415069000	-0.183920000
6	-2.639634000	-0.818454000	-0.103530000
6	-3.688699000	-1.610506000	-0.553353000
6	-3.579064000	-2.988950000	-0.472145000
6	-2.426782000	-3.546250000	0.054040000
6	-1.421867000	-2.690780000	0.472531000
7	-1.523271000	-1.366081000	0.394497000
1	-4.579809000	-1.169090000	-0.970149000
1	-4.387152000	-3.617959000	-0.818477000
1	-2.301496000	-4.615184000	0.138732000
1	-0.500179000	-3.076935000	0.888054000
6	-2.687353000	0.673502000	-0.148967000

6	-3.830855000	1.369706000	-0.520359000
6	-3.806333000	2.754400000	-0.538301000
6	-1.546854000	2.652468000	0.180259000
7	-1.566266000	1.321232000	0.197861000
1	-4.736970000	0.848060000	-0.783827000
1	-4.689887000	3.308266000	-0.823200000
1	-2.580455000	4.492809000	-0.182501000
1	-0.615277000	3.121109000	0.470257000
6	3.190802000	-0.338748000	-1.206603000
6	3.518316000	0.279967000	-2.567481000
6	1.847235000	-1.066376000	-1.298065000
6	4.278737000	-1.352895000	-0.845238000
1	3.658599000	-0.510914000	-3.304161000
1	2.716128000	0.925007000	-2.925065000
1	4.438795000	0.864315000	-2.523219000
1	1.020962000	-0.391905000	-1.537062000
1	1.893724000	-1.804298000	-2.099642000
1	1.613491000	-1.607067000	-0.378121000
1	4.346054000	-2.116337000	-1.620073000
1	5.253535000	-0.871429000	-0.758651000
1	4.068361000	-1.858923000	0.098380000
6	2.909951000	0.262758000	1.228332000
8	1.802544000	0.007274000	1.681393000
8	3.988847000	0.066137000	1.959811000
1	3.731034000	-0.293444000	2.822914000
6	2.227609000	1.960563000	-0.451249000
1	2.587436000	2.522308000	-1.309526000
1	1.220802000	1.610535000	-0.683802000
1	2.164529000	2.648315000	0.392196000
g			
29	0.370215000	0.291388000	0.054018000
6	-4.140631000	0.976125000	0.192932000
1	-4.346501000	1.812261000	-0.482043000
6	2.386119000	-3.611731000	0.182784000
6	3.280747000	0.517906000	-0.057791000
6	4.564326000	1.042717000	-0.135116000
6	4.731199000	2.415609000	-0.212345000
6	3.615255000	3.233893000	-0.210987000
6	2.369031000	2.636282000	-0.132095000
7	2.205088000	1.317202000	-0.057708000
1	5.429006000	0.398725000	-0.136998000
1	5.724398000	2.838047000	-0.273059000
1	3.699958000	4.308482000	-0.269726000

1	1.466074000	3.232073000	-0.128160000
6	3.017832000	-0.949257000	0.027557000
6	4.038540000	-1.891081000	0.050221000
6	3.716912000	-3.236107000	0.128679000
6	1.426322000	-2.614513000	0.157261000
7	1.731888000	-1.321186000	0.082221000
1	5.073255000	-1.590995000	0.008644000
1	4.500831000	-3.980222000	0.147382000
1	2.091756000	-4.648546000	0.244112000
1	0.372427000	-2.855893000	0.198379000
6	-4.878965000	-0.274473000	-0.373581000
6	-6.383569000	-0.005939000	-0.331082000
6	-4.550362000	-1.523461000	0.443143000
6	-4.446088000	-0.479134000	-1.826241000
1	-6.909282000	-0.813553000	-0.839917000
1	-6.761832000	0.039708000	0.689166000
1	-6.635262000	0.927667000	-0.837699000
1	-4.882652000	-1.433548000	1.476934000
1	-5.058171000	-2.384920000	0.009800000
1	-3.479020000	-1.734920000	0.440115000
1	-4.974589000	-1.329757000	-2.255419000
1	-4.675631000	0.398408000	-2.433130000
1	-3.376153000	-0.680845000	-1.904166000
6	-4.516405000	1.389484000	1.622322000
1	-3.939707000	2.257405000	1.935255000
1	-5.569726000	1.653545000	1.657810000
1	-4.331185000	0.582204000	2.327659000
6	-2.693346000	0.771191000	0.131031000
7	-1.562081000	0.599608000	0.106684000
h			
29	0.287517000	-0.357987000	-0.492130000
6	-4.221474000	-0.989511000	0.200979000
1	-3.712917000	-1.690671000	0.850475000
6	2.148576000	3.626117000	-0.291104000
6	3.156471000	-0.451778000	0.224118000
6	4.415387000	-0.915281000	0.585541000
6	4.622376000	-2.279678000	0.707137000
6	3.572080000	-3.148103000	0.466429000
6	2.346081000	-2.607297000	0.115148000
7	2.145073000	-1.298116000	-0.000552000
1	5.228496000	-0.232255000	0.773143000
1	5.596174000	-2.656826000	0.986696000
1	3.692305000	-4.217975000	0.547061000

1	1.490304000	-3.239409000	-0.083606000
6	2.849509000	1.000226000	0.059674000
6	3.814851000	1.988893000	0.207884000
6	3.458250000	3.315216000	0.031054000
6	1.245756000	2.584694000	-0.422814000
7	1.584221000	1.309562000	-0.250993000
1	4.834575000	1.737164000	0.451815000
1	4.198915000	4.094939000	0.141219000
1	1.828839000	4.646296000	-0.441176000
1	0.211145000	2.772530000	-0.678992000
6	-4.391632000	0.420038000	0.646390000
6	-5.610420000	0.353358000	1.602287000
6	-4.677992000	1.355982000	-0.524925000
6	-3.168700000	0.888228000	1.436878000
1	-5.783438000	1.354720000	1.994208000
1	-6.509608000	0.023173000	1.083445000
1	-5.424602000	-0.311687000	2.445947000
1	-5.634864000	1.143991000	-1.007058000
1	-4.737814000	2.378506000	-0.155473000
1	-3.876076000	1.299204000	-1.262100000
1	-3.406717000	1.832663000	1.924858000
1	-2.897408000	0.166596000	2.208622000
1	-2.314079000	1.024609000	0.779546000
17	-1.799645000	-0.996491000	-0.975306000
7	-4.848691000	-1.484777000	-0.810137000
1	-4.741300000	-2.457023000	-1.062591000
1	-5.304888000	-0.879889000	-1.480372000
	i		
29	0.097329000	-0.280213000	0.915976000
6	-2.994291000	0.583296000	0.063251000
1	-2.124233000	1.244921000	0.128007000
6	1.981628000	3.628167000	0.213169000
6	2.804690000	-0.509894000	-0.160663000
6	3.984752000	-1.028937000	-0.678322000
6	4.146561000	-2.402942000	-0.748967000
6	3.129104000	-3.228245000	-0.303457000
6	1.981105000	-2.636026000	0.194736000
7	1.822615000	-1.316385000	0.263330000
1	4.772276000	-0.380967000	-1.028026000
1	5.059749000	-2.820674000	-1.149154000
1	3.215018000	-4.303810000	-0.338658000
1	1.155888000	-3.236686000	0.554148000
6	2.557925000	0.959080000	-0.046857000
6	3.517903000	1.902243000	-0.392617000
6	3.225027000	3.249456000	-0.262011000
6	1.079711000	2.631131000	0.540974000
7	1.356042000	1.334471000	0.414276000
1	4.487305000	1.600023000	-0.754803000
1	3.963767000	3.992777000	-0.527197000
1	1.711686000	4.666562000	0.332668000
1	0.097615000	2.877416000	0.922820000
6	-2.934561000	-0.090683000	-1.343380000
6	-2.957783000	1.027808000	-2.386881000
6	-4.131893000	-1.018823000	-1.554125000
6	-1.637232000	-0.884513000	-1.510367000
1	-2.908616000	0.595465000	-3.386351000
1	-3.863430000	1.622844000	-2.305282000
1	-2.099608000	1.691974000	-2.263518000
1	-5.075031000	-0.475629000	-1.497260000
1	-4.075875000	-1.460865000	-2.548755000
1	-4.155563000	-1.834054000	-0.829734000
1	-1.595198000	-1.299860000	-2.517147000
1	-0.760887000	-0.236428000	-1.397748000
1	-1.557287000	-1.720313000	-0.812029000
6	-2.793131000	-0.437139000	1.169997000
8	-1.705719000	-0.783729000	1.610523000
8	-3.906155000	-0.944783000	1.658546000
1	-3.689980000	-1.585376000	2.353592000
7	-4.187792000	1.382227000	0.217081000
1	-4.160531000	1.946037000	1.056136000
1	-5.017762000	0.804848000	0.262764000
	j		
29	0.377111000	-0.305113000	-0.053801000
6	-4.159110000	-0.991101000	-0.169073000
1	-4.377227000	-1.809754000	0.520494000
6	2.360164000	3.616119000	-0.164029000
6	3.288316000	-0.507500000	0.053036000
6	4.576330000	-1.022218000	0.124330000
6	4.754702000	-2.393965000	0.195718000
6	3.645508000	-3.221379000	0.194649000
6	2.394286000	-2.633723000	0.121465000
7	2.219192000	-1.315692000	0.052529000
1	5.435749000	-0.371235000	0.125876000
1	5.751517000	-2.808450000	0.251787000
1	3.739197000	-4.295440000	0.249285000
1	1.496348000	-3.237028000	0.118027000

6	3.013407000	0.957920000	-0.024583000
6	4.026404000	1.908122000	-0.040786000
6	3.693932000	3.250956000	-0.111329000
6	1.408489000	2.611019000	-0.145133000
7	1.724490000	1.319741000	-0.077883000
1	5.063503000	1.616342000	0.000290000
1	4.471788000	4.001517000	-0.124746000
1	2.057451000	4.650881000	-0.219046000
1	0.352696000	2.844193000	-0.185096000
6	-4.901250000	0.270134000	0.346001000
6	-6.386239000	-0.076102000	0.445541000
6	-4.694004000	1.432588000	-0.623784000
6	-4.363970000	0.637070000	1.729030000
1	-6.942611000	0.800316000	0.777174000

1	-6.781076000	-0.396469000	-0.515979000
1	-6.553157000	-0.873706000	1.171435000
1	-5.152735000	1.233372000	-1.592513000
1	-5.162006000	2.331133000	-0.222729000
1	-3.633221000	1.650703000	-0.772006000
1	-4.948816000	1.457864000	2.142626000
1	-4.439709000	-0.205313000	2.419368000
1	-3.322187000	0.960133000	1.691028000
6	-2.688820000	-0.780472000	-0.095039000
7	-1.555632000	-0.617926000	-0.097844000
7	-4.571104000	-1.387152000	-1.496628000
1	-4.243584000	-2.309673000	-1.747296000
1	-4.275631000	-0.728719000	-2.205045000
